# The *Kalanchoë* genome provides insights into convergent evolution and building blocks of crassulacean acid metabolism

**DOI:** 10.1038/s41467-017-01491-7

**Published:** 2017-12-01

**Authors:** Xiaohan Yang, Rongbin Hu, Hengfu Yin, Jerry Jenkins, Shengqiang Shu, Haibao Tang, Degao Liu, Deborah A. Weighill, Won Cheol Yim, Jungmin Ha, Karolina Heyduk, David M. Goodstein, Hao-Bo Guo, Robert C. Moseley, Elisabeth Fitzek, Sara Jawdy, Zhihao Zhang, Meng Xie, James Hartwell, Jane Grimwood, Paul E. Abraham, Ritesh Mewalal, Juan D. Beltrán, Susanna F. Boxall, Louisa V. Dever, Kaitlin J. Palla, Rebecca Albion, Travis Garcia, Jesse A. Mayer, Sung Don Lim, Ching Man Wai, Paul Peluso, Robert Van Buren, Henrique Cestari De Paoli, Anne M. Borland, Hong Guo, Jin-Gui Chen, Wellington Muchero, Yanbin Yin, Daniel A. Jacobson, Timothy J. Tschaplinski, Robert L. Hettich, Ray Ming, Klaus Winter, James H. Leebens-Mack, J. Andrew C. Smith, John C. Cushman, Jeremy Schmutz, Gerald A. Tuskan

**Affiliations:** 1 Biosciences Division, Oak Ridge National Laboratory, Oak Ridge, TN 37831 USA; 2 The Bredesen Center for Interdisciplinary Research and Graduate Education, University of Tennessee, Knoxville, TN 37996 USA; 3 HudsonAlpha Institute for Biotechnology, 601 Genome Way, Huntsville, AL 35801 USA; 4 US Department of Energy Joint Genome Institute, 2800 Mitchell Drive, Walnut Creek, CA 94598 USA; 5 Center for Genomics and Biotechnology, Fujian Provincial Key Laboratory of Haixia Applied Plant Systems Biology, Fujian Agriculture and Forestry University, Fuzhou, Fujian 350002 China; 6 Department of Biochemistry and Molecular Biology, University of Nevada, Reno, NV 89557 USA; 7 Department of Plant Biology, University of Georgia, Athens, GA 30602 USA; 8 Department of Biochemistry & Cellular and Molecular Biology, University of Tennessee, Knoxville, TN 37996 USA; 9 Department of Biological Sciences, Northern Illinois University, DeKalb, IL 60115 USA; 10 Department of Plant Sciences, Institute of Integrative Biology, University of Liverpool, Liverpool, L69 7ZB UK; 11 Chemical Sciences Division, Oak Ridge National Laboratory, Oak Ridge, TN 37831 USA; 12 Department of Plant Sciences, University of Oxford, Oxford, OX1 3RB UK; 13 Department of Plant Biology, University of Illinois at Urbana-Champaign, Urbana, IL 61801 USA; 14 Pacific Biosciences, Inc., 940 Hamilton Avenue, Menlo Park, CA 94025 USA; 15 Department of Horticulture, Michigan State University, East Lansing, MI 48824 USA; 16 Department of Plant Sciences, University of Tennessee, Knoxville, TN 37996 USA; 17 School of Natural and Environmental Science, Newcastle University, Newcastle upon Tyne, NE1 7RU UK; 18 Smithsonian Tropical Research Institute, Apartado, Balboa, Ancón 0843-03092 Republic of Panama

## Abstract

Crassulacean acid metabolism (CAM) is a water-use efficient adaptation of photosynthesis that has evolved independently many times in diverse lineages of flowering plants. We hypothesize that convergent evolution of protein sequence and temporal gene expression underpins the independent emergences of CAM from C_3_ photosynthesis. To test this hypothesis, we generate a de novo genome assembly and genome-wide transcript expression data for *Kalanchoë fedtschenkoi*, an obligate CAM species within the core eudicots with a relatively small genome (~260 Mb). Our comparative analyses identify signatures of convergence in protein sequence and re-scheduling of diel transcript expression of genes involved in nocturnal CO_2_ fixation, stomatal movement, heat tolerance, circadian clock, and carbohydrate metabolism in *K. fedtschenkoi* and other CAM species in comparison with non-CAM species. These findings provide new insights into molecular convergence and building blocks of CAM and will facilitate CAM-into-C_3_ photosynthesis engineering to enhance water-use efficiency in crops.

## Abstract (web summary)

Crassulacean acid metabolism (CAM) is a metabolic adaptation of photosynthesis that enhances water use efficiency. Here, via genomic analysis of *Kalanchoë*, the authors provide evidence for convergent evolution of protein sequence and temporal gene expression underpinning the multiple independent emergences of CAM.

## Introduction

Crassulacean acid metabolism (CAM) is a metabolic adaptation of photosynthetic CO_2_ fixation that enhances plant water-use efficiency (WUE) and associated drought avoidance/tolerance by reducing transpirational water loss through stomatal closure during the day, when temperatures are high, and stomatal opening during the night, when temperatures are lower^1^. In the face of the rapidly increasing human population and global warming predicted over the next century, the outstanding WUE of CAM plants highlights the potential of the CAM pathway for sustainable food and biomass production on semi-arid, abandoned, or marginal agricultural lands^2–4^.

CAM photosynthesis can be divided into two major phases: (1) nocturnal uptake of atmospheric CO_2_ through open stomata and primary fixation of CO_2_ by phosphoenolpyruvate carboxylase (PEPC) to oxaloacetate (OAA) and its subsequent conversion to malic acid by malate dehydrogenase; and (2) daytime decarboxylation of malate and CO_2_ refixation via C_3_ photosynthesis, mediated by ribulose-1,5-bisphosphate carboxylase/oxygenase (RuBisCO)^5,6^. Malic acid is stored in the vacuole of photosynthetically active cells reaching a peak at dawn and can be used as a reference point to divide the two phases. CAM is found in over 400 genera across 36 families of vascular plants^4^ and is thought to have evolved multiple times independently from diverse ancestral C_3_ photosynthesis lineages^7^. The core biochemical characteristics of the CAM cycle are similar in all the plant lineages in which CAM has evolved, with some variation in the enzymes that catalyze malate decarboxylation during the day, and in the storage carbohydrates that provide substrates for malic acid synthesis at night^8,9^.

We hypothesize that convergent evolution in protein sequence and/or temporal diel gene expression underpins the multiple and independent emergences of CAM from C_3_ photosynthesis. Convergent evolution is generally defined as the appearance of similar phenotypes in distinct evolutionary lineages^10^. Although phenotypic convergence is widely recognized, its evolutionary mechanism has been extensively debated. Morris^11^ argues that the evolutionary course is not random but selection-constrained, along certain pathways, to arrive at the same solution or outcome. Recently, comparative genomics analysis began to provide new insight into the molecular mechanism of convergent evolution. For example, Foote et al.^12^ performed comparative genomic analyses of three species of marine mammals (the killer whale, walrus, and manatee) that share independently evolved phenotypic adaptations to a marine existence, and identified convergent amino-acid substitutions in genes evolving under positive selection and putatively associated with a marine phenotype. Also, Hu et al.^13^ compared the genomes of the bamboo-eating giant and red pandas, two obligate bamboo-feeders that independently possess adaptive pseudothumbs, and identified 70 adaptively convergent genes (i.e., under positive selection in these two species), of which nine genes, featuring nonrandom convergent amino-acid substitution between giant and red pandas, are closely related to limb development and essential nutrient utilization. These two examples indicate that specific amino-acid replacements at a small number of key sites can result in highly predictable convergent outcomes, supporting the constrained selection theory of Morris^11^. However, such predictable protein sequence convergence was not found in the convergence of hemoglobin function in high-altitude-dwelling birds, indicating that possible adaptive solutions are perhaps contingent upon prior evolutionary history^14^. This finding supports the contingent adaptation theory^15^ that evolution is contingent upon history and consequently replaying life’s tape will give different outcomes. In addition to protein sequence convergence, convergent changes in gene expression were found to be associated with convergent evolution of vocal learning in the brains of humans and song-learning birds^16^. Therefore, convergent changes in both protein sequence and gene expression are important aspects of the molecular basis of convergent evolution.

We sought to investigate whether changes in protein sequence and/or gene expression contribute to the evolutionary convergence of CAM through genome-wide screening for signatures of convergent changes in protein sequences and diel mRNA expression patterns that meet the following criteria: the signatures are (1) isomorphic in the CAM genomes of distant groups, such as eudicots and monocots, which diverged ~135 million years ago^17^, and (2) dimorphic in related C_3_ photosynthesis genomes. Recently, the genome sequences of two monocot CAM species, *Ananas comosus* (L.) Merr. (pineapple)^18^, and *Phalaenopsis equestris* (Schauer) Rchb.f. (moth orchid)^19^, were published. Here we present the genome sequence of *Kalanchoë fedtschenkoi* Raym.-Hamet & H. Perrier, which is an emerging molecular genetic model for obligate CAM species in the eudicots^4,6,20^. Our analyses reveal the genomic signatures of convergence shared between eudicot (represented by *Kalanchoë*) and monocot (represented by pineapple and orchid) CAM species.

## Results

### *Kalanchoë* genome assembly and annotation

The diploid *K. fedtschenkoi* (2n = 2x = 34 chromosomes; Supplementary Fig. 1) genome size was estimated to be ~260 Mb (Supplementary Table 1). The *K. fedtschenkoi* genome was assembled from ~70× paired-end reads and ~37× mate-pair reads generated using an Illumina MiSeq platform (Supplementary Table 2 and Supplementary Fig. 2). The genome assembly consisted of 1324 scaffolds with a total length of 256 Mb and scaffold N50 of 2.45 Mb (Supplementary Table 3), in which we predicted and annotated 30,964 protein-coding genes (Supplementary Table 4).

### The phylogenetic placement of *Kalanchoë*

*Kalanchoë* is the first eudicot CAM lineage with a genome sequence to date and serves as an important reference for understanding the evolution of CAM. In addition, *K. fedtschenkoi* is the first sequenced species in the distinct eudicot lineage, Saxifragales. Although the monophyly of this morphologically diverse order is well supported by molecular data, its phylogenetic placement has been less clear^21,22^. The recent consensus view, based mainly on analyses of plastid DNA sequences, has placed the Saxifragales as a sister group to the rosids, and together they comprise the large clade of superrosids^23,24^. However, there have been indications of conflict between trees based on plastid genomes and nuclear genomes for this clade^19,24^. Additionally, the major lineages of core eudicots are thought to have diversified rapidly following their first appearance, making resolution of the relationships among these clades particularly challenging^17,25^ and implicating incomplete lineage sorting (ILS) as a potentially important process that would result in discordance among gene histories^26^.

**Fig. 1:**
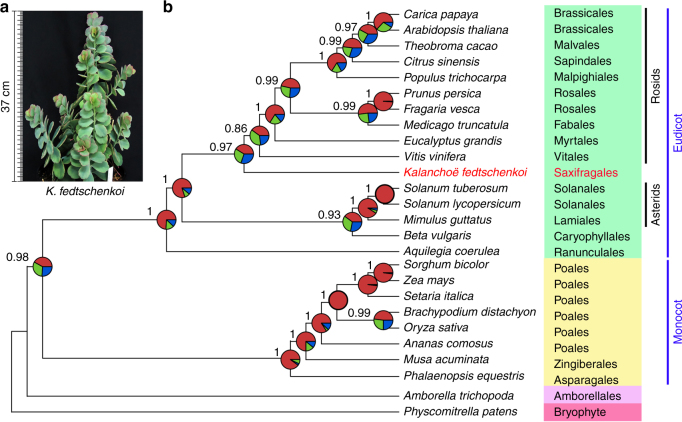
A species tree reconstructed from 210 single-copy genes using a summary method. **a** Diploid plant of *Kalanchoë fedtschenkoi*. **b** Individual maximum-likelihood gene trees were reconstructed from the CDS alignments for each of the 210 single-copy-gene ortholog groups using RAxML^78^, and the species tree was summarized from the gene trees using ASTRAL-II^79^. Pie graphs on nodes represent the proportion of gene trees that support the various quartets at every node, with red for the main topology shown in this tree, blue for the first alternative, and green for the second alternative, respectively. Quartet frequencies displayed in pie graphs and the posterior-probability at each node are calculated by ASTRAL-II^79^

We performed phylogenetic analyses with 210 single-copy nuclear genes from 26 sequenced plant genomes using multiple phylogenetic inference strategies. The resulting species trees are congruent with each other except for the placement of *K. fedtschenkoi*, which was placed either as sister to the rosids in a phylogenetic tree reconstructed using a quartet-based coalescent species tree method (Fig. 1) or as sister to all other core eudicots as revealed by alternative phylogenetic trees reconstructed from (1) concatenated protein sequence alignment without gene partition using maximum-likelihood (Supplementary Fig. 3), (2) a partitioned analysis of multi-gene alignment using maximum-likelihood and Bayesian methods (Supplementary Fig. 4), and (3) analysis of individual gene trees using fully Bayesian multispecies coalescent method (Supplementary Fig. 5). Despite substantial discordance among estimated nuclear gene trees, the coalescence-based tree was consistent with the results of the plastome-based analyses, placing *Kalanchoë* as sister to the rosids (Fig. 1). Coalescent species tree estimation can account for gene tree discordance due to ILS^27^. At the same time, alternative placements of *Kalanchoë* as sister to the asterids, or as sister to all other core eudicots were observed in many gene trees (Fig. 1 and Supplementary Fig. 5). Gene tree discordance due to rapid diversification early in eudicot history has also been characterized by others^24^. Regardless of the optimal placement of the Saxifragales, including *Kalanchoë*, individual gene trees will often have alternative histories due to ILS in the face of rapid species diversification.

### *Kalanchoë* genome duplication

**Fig. 2:**
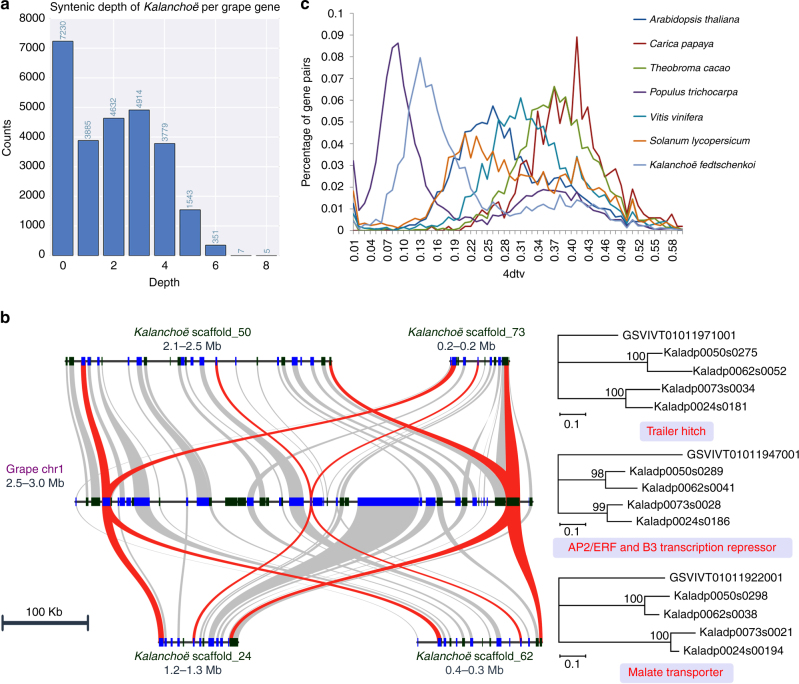
Genome duplication in *Kalanchoë fedtschenkoi*. **a** Syntenic depth of the *K. fedtschenkoi* genome for each grape gene. Syntenic depth refers to the number of times a genomic region is covered by synteny blocks against another genome. **b** Typical micro-colinearity patterns between genomic regions from grape and *K. fedtschenkoi*. Rectangles show predicted gene models with colors showing relative orientations (blue: same strand, black: opposite strand). Matching gene pairs are displayed as connecting shades. Three orthologous gene groups that were maximally retained as four copies in *K. fedtschenkoi* were highlighted with phylogenetic trees on the right suggesting two rounds of genome duplications in the *Kalanchoë* lineage. **c** Four-fold transversion substitution rate (4dtv) in *K. fedtschenkoi* and six other eudicot plant species

The grape genome has no additional genome duplication after the ancestral gamma hexaploidization^28,29^ and is the best available reference for studying ancestral eudicot genome duplication events. Syntenic depth analyses^30,31^ showed that there are multiple *K. fedtschenkoi* blocks covering each grape gene (Fig. 2a and Supplementary Fig. 6). Specifically, 65% of the grape genome had from one to four syntenic blocks in *K. fedtschenkoi*. In contrast, a sudden drop in syntenic depth occurred after a depth of 4× (Fig. 2a), indicating that each grape genome region has up to four *K. fedtschenkoi* blocks and thus providing strong evidence for two distinct whole-genome duplications (WGDs) events in *K. fedtschenkoi*. The microsynteny patterns further support two WGDs on the lineages leading to *K. fedtschenkoi*. Specifically, the microsynteny pattern reflects a 1:4 gene copy ratio between the grape genome and the diploid *K. fedtschenkoi* genome (Fig. 2b).

From the *Kalanchoë* point of view, we found that 49% of the *Kalanchoë* genome was covered by one grape-*Kalanchoë* block, 7% covered in two grape-*Kalanchoë* blocks, and 1% covered in three grape-*Kalanchoë* blocks (Supplementary Fig. 7). This suggests that we could often find one best grape-*Kalanchoë* block out of the three gamma triplicated regions in grape. This fits the scenario that the gamma WGD predated the divergence and there has been no WGD in the grape lineage since grape-*Kalanchoë* diverged. Alternatively, if the divergence predated the gamma WGD, then from the *Kalanchoë* point of view we should instead see three matching grape regions. Hence, the grape-*Kalanchoë* genome comparisons strongly supported the gamma WGD as a shared event, and further supported the phylogenetic position of *Kalanchoë* in Fig. 1.

Despite two apparent WGDs in the *K. fedtschenkoi* lineage, synonymous substitutions per synonymous site (Ks) between duplicate gene pairs showed only one prominent peak ~0.35 (Supplementary Fig. 8). The unimodal distribution of Ks suggests the two WGD events occurring close in time. Similarly, two distinct peaks appear in the distribution of the four-fold transversion substitution rate (4dtv) values between the *K. fedtschenkoi* gene pairs (Fig. 2c). Grape-*Kalanchoë* gene pairs show a prominent peak around Ks = 1.5 (Supplementary Fig. 8), indicating that the WGDs in the *K. fedtschenkoi* lineage occurred well after its divergence from grape early in the history of the rosid lineage.

### Gene co-expression modules and clusters in *Kalanchoë*

**Fig. 3:**
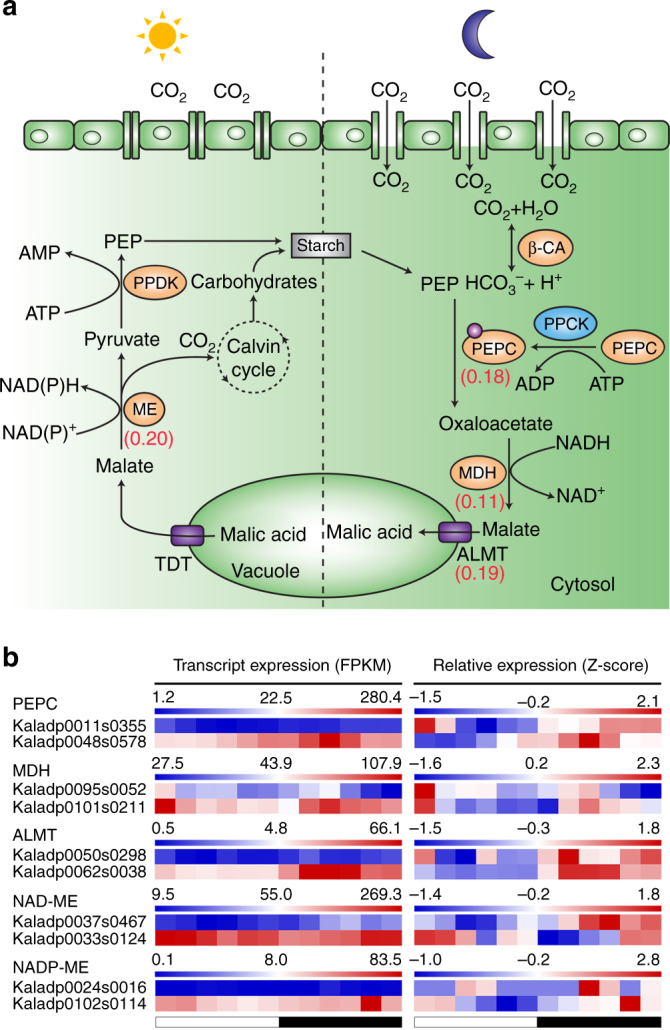
An overview of CAM pathway in *Kalanchoë fedtschenkoi*. **a** The CAM pathway map in *K. fedtschenkoi*. Orange colors indicate the key enzymes involved in the CAM pathway. The numbers in parenthesis are the four-fold transversion substitution rate (4dtv) values. **b** Diel expression profiles of duplicated genes in CAM-related gene families. ALMT tonoplast aluminum-activated malate transporter, β-CA β type carbonic anhydrase, ME malic enzyme, MDH malate dehydrogenase, PEP phosphoenolpyruvate, PEPC PEP carboxylase, PPCK PEPC kinase, PPDK pyruvate phosphate dikinase, TDT tonoplast dicarboxylate transporter. White and black bars indicate daytime (12-h) and nighttime (12-h), respectively

To elucidate gene function in *K. fedtschenkoi*, we performed a weighted correlation network analysis of transcript expression in 16 samples including 12 mature leaf samples collected every 2 h over a 24-h period and four non-leaf samples collected 4 h after the beginning of the light period, including shoot tip (leaf pair 1 plus the apical meristem), stem (between leaf pair 3 and leaf pair 8), root, and flower. Our analysis identified 25 co-expression modules, among which one module (MEblack containing 782 genes) was significantly (Student’s *t*-test, *P < *0.001) associated with the leaf samples collected during the dark period (Supplementary Fig. 9), with an increase in transcript abundance at night (Supplementary Fig. 10). Several biological processes (e.g., carboxylic acid biosynthesis, terpene biosynthesis, and lipid metabolism) were over-represented (hypergeometric enrichment test, *P < *0.05) (Supplementary Data 1), and several key genes encoding proteins involved in nocturnal CAM carboxylation and vacuolar uptake of malate such as Kaladp0018s0289 (*β-CA*), Kaladp0048s0578 (*PEPC2*), Kaladp0037s0517 (*PPCK*), Kaladp0022s0111 (*MDH*), and Kaladp0062s0038 (*ALMT6*) were present in this module (Fig. 3a, Supplementary Note 1 and Supplementary Table 5). These results suggest that genes in the co-expression module MEblack play important roles in the nighttime processes that define CAM. One alternate module (MEblue containing 1911 genes) was significantly correlated with the leaf samples collected during the day (Supplementary Fig. 9), with an increase in transcript abundance during the light period (Supplementary Fig. 10). Several biological processes (e.g., starch biosynthesis, coenzyme biosynthetic process) were over-represented (hypergeometric enrichment test, *P* < 0.05) in this module (Supplementary Data 1). One gene in the CAM decarboxylation process, Kaladp0010s0106 (*PPDK-RP*), belongs to this module (Supplementary Table 6).

We also performed cluster analysis on the CAM leaf time-course expression data for the transcripts that showed significantly (ANOVA of glm models where H_0_ = a flat line, *P < *0.05) time-structured diel expression patterns as determined by a polynomial regression. Clustering of transcripts with time-structured expression identified 11 clusters (Supplementary Fig. 11 and Supplementary Table 7). Networks constructed for each cluster implicated highly connected hub genes and their direct or indirect interactions with CAM-related genes (Supplementary Data 2). For example, cluster 7, which contains *PEPC1* (Kaladp0095s0055) and *PPCK2* (Kaladp0604s0001), has a zinc-finger protein *CONSTANS-like* gene as a central hub (Supplementary Data 2). *CONSTANS-like* genes are part of the circadian clock regulatory network^32^. Similarly, multiple *REVEILLE* transcripts, which encode transcription factors for genes with evening elements in their promoters^33^, are hubs in cluster 4 that contains *NADP-ME* genes (Kaladp0092s0166) (Supplementary Data 2).

### Overview of genes that have undergone convergent evolution

**Fig. 4:**
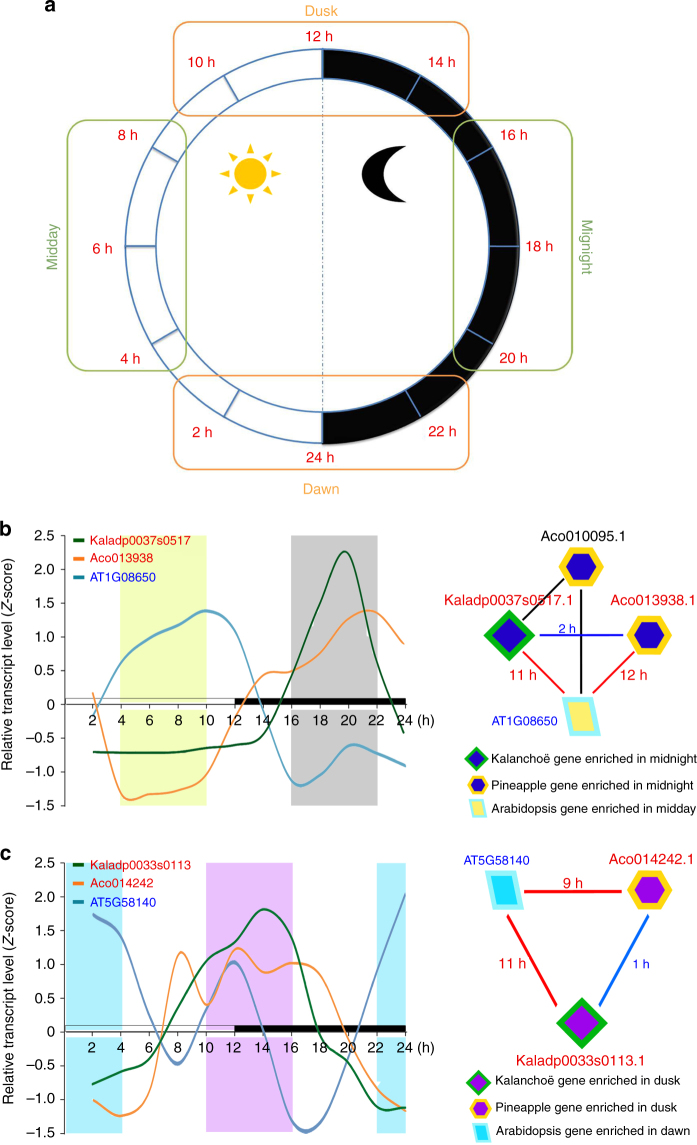
Examples of convergent change in diel transcript expression pattern in CAM species. **a** The four time-windows for comparative analysis of temporal changes in transcript expression, which were represented by 12 time points: 2, 4, …, 24 h after the beginning of the light period. **b** and **c** Comparison of diel transcript expression pattern of phosphoenolpyruvate carboxylase kinase 1 (*PPCK1*) and phototropin 2 (*PHOT2*), respectively, between CAM species (*Kalanchoë fedtschenkoi* and pineapple) and C_3_ species (*Arabidopsis*). Left panels show the diel transcript expression profiles. Right panels show enrichment triangle networks, in which a *K. fedtschenkoi* gene and a pineapple ortholog had significantly enriched expression in the same time-window, whereas an *Arabidopsis* ortholog had significantly enriched expression in the opposite time-window. The numbers are the time shifts in diel transcript expression pattern between genes connected by each edge. White and black bars indicate daytime (12-h) and nighttime (12-h), respectively. X-axis represents the time after the beginning of the light period

To determine the possibility that the diel reprogramming of metabolism that distinguishes CAM from C_3_ photosynthesis was achieved, at least in part, by convergent shifts in diel patterns of gene expression, we performed comparative analysis of diel transcript abundance patterns in CAM and C_3_ photosynthesis species. Specifically, we compared the diel expression patterns of 9733 ortholog groups of genes from *K. fedtschenkoi* (eudicot, CAM photosynthesis), *A. comosus* (monocot, CAM photosynthesis), and *Arabidopsis thaliana* (eudicot, C_3_ photosynthesis), with transcript abundances >0.01 FPKM in mature leaf samples collected at six or more diel time points. Sampling time points included dawn (22, 24, and 2 h from the start of the light period), midday (4, 6, and 8 h from the start of the light period), dusk (10, 12, and 14 h from the start of the light period), and midnight (16, 18, and 20 h from the start of the light period) (Fig. 4a). A gene from *K. fedtschenkoi* was defined as having undergone convergent evolution of gene expression if it met all of the following criteria: (1) its diel transcript expression pattern was highly correlated (Spearman’s rank correlation coefficient, *r* > 0.8) with those of at least one of the orthologs in *A. comosus*, but not highly correlated (*r* < 0.5) with those of any of the orthologs in *A. thaliana*; (2) it displayed a significant difference (false discovery rate <0.01) in transcript abundance either between midday and midnight (e.g., Fig. 4b), or between dawn and dusk (e.g., Fig. 4c); and (3) the time shift between *K. fedtschenkoi* and *A. comosus* transcript time-courses was less than or equal to 3 h, whereas the time shifts between CAM species (*K. fedtschenkoi* and *A. comosus*) transcripts and their *A. thaliana* ortholog transcript were equal to or greater than 6 h. Based on these criteria, 54 *K. fedtschenkoi* genes were identified as candidates for involvement in the convergent shift in diel gene expression patterns specific to the two CAM species relative to *A. thaliana* (Supplementary Note 2, Supplementary Data 3 and Supplementary Table 8).

To identify genes that had likely undergone convergent evolution in protein sequence in the CAM species, we reconstructed gene tribes based on protein sequences from the species listed in Supplementary Fig. 4. We then created phylogenetic trees for the genes from all tribes that include at least one gene from each of the 13 studied species (Supplementary Table 9). A *K. fedtschenkoi* gene was defined as having undergone convergent evolution in protein sequence if it met all of the following criteria: (1) the *K. fedtschenkoi* gene is clustered with gene(s) from at least one of the two monocot CAM species (*A. comosus* and *P. equestris*) in a phylogenetic clade containing no genes from C_3_ or C_4_ photosynthesis species; (2) convergent amino-acid changes were detected between the *K. fedtschenkoi* gene with gene(s) from at least one of the two monocot CAM species; and (3) the *K. fedtschenkoi* gene shared at least one amino-acid mutation with its ortholog in at least one of the two monocot CAM species, as compared with C_3_ and C_4_ photosynthesis species. A total of four *K. fedtschenkoi* genes showing convergent changes in protein sequences were identified (Supplementary Figs. 12–15 and Supplementary Table 10).

We also performed genome-wide positive selection analysis in each of the three CAM species (i.e., *A. comosus*, *P. equestris*, and *K. fedtschenkoi*) in comparison with 21 non-CAM species (Supplementary Method 1) and identified two genes that were under positive selection in the dicot CAM species *K. fedtschenkoi* and one of the monocot CAM species (Supplementary Figs. 16–17).

### Convergent evolution of genes involved in CO_2_ fixation

**Fig. 5:**
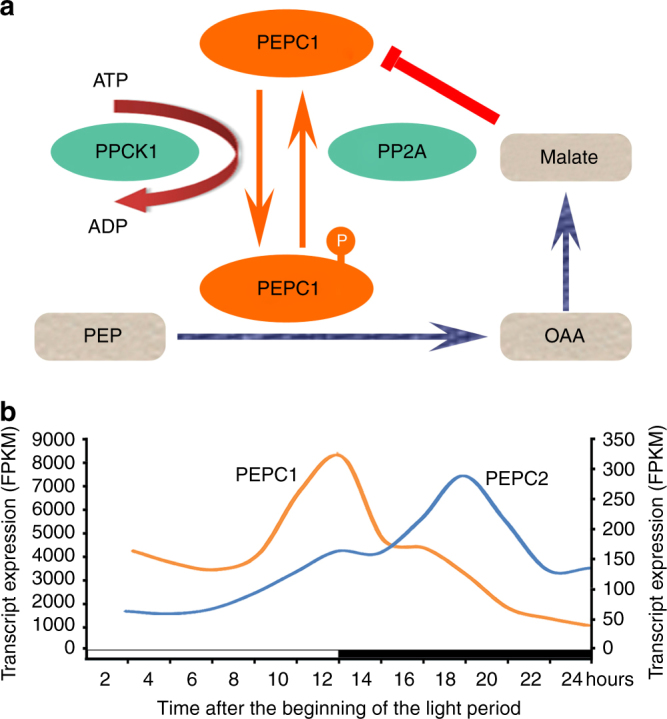
Two phosphoenolpyruvate carboxylase (PEPC) genes with relative high transcript abundance in *Kalanchoë fedtschenkoi*. **a** Regulation of PEPC1 activity. **b** Diel expression profiles of *PEPC1* (Kaladp0095s0055.1) and *PEPC2* (Kaladp0048s0578.1) transcripts in *K. fedtschenkoi*, shown in the left and right *Y*-axis, respectively. OAA Oxaloacetate, PEP phospho*enol*pyruvate, PEPC PEP carboxylase, PPCK PEPC kinase, PP2A protein phosphatase 2 A. White and black bars indicate daytime (12-h) and nighttime (12-h), respectively

PEPC is a key enzyme for nocturnal CO_2_ fixation and PPCK is a pivotal protein kinase that regulates PEPC in response to the circadian clock in CAM plants^4,6,34^. PPCK phosphorylates PEPC in the dark (Fig. 5a) and thereby reduces malate inhibition of PEPC activity, promoting nocturnal CO_2_ uptake^35,36^. Multiple PPCK genes were identified in the *K. fedtschenkoi* genome, among which two genes (Kaladp0037s0517 and Kaladp0604s0001) showed higher transcript abundance than the others in CAM leaves (Supplementary Table 5). The diel expression patterns of the most abundant PPCK transcripts in *K. fedtschenkoi* (Kaladp0037s0517.1) and *A. comosus* (Aco013938.1) were highly correlated, with only a 1.5-hour time shift between them, whereas both showed an ~11-hour time shift relative to their best matched ortholog in *Arabidopsis* (AT1G08650) (Fig. 4b and Supplementary Table 8). Peak PPCK transcript abundance was shifted from daytime in C_3_ photosynthesis species (*Arabidopsis*) to nighttime in the two CAM species (Fig. 4b), which suggests convergence and is consistent with PPCK activation of PEPC-mediated nocturnal CO_2_ fixation. Among the PEPC genes identified in *K. fedtschenkoi*, Kaladp0095s0055 and Kaladp0048s0578 showed higher transcript abundance than the others (Supplementary Table 5). Kaladp0095s0055 (named *PEPC1* herein) was an abundant transcript throughout both the light and the dark period, with its peak transcript level phased to dusk. The second most abundant PEPC transcript (Kaladp0048s0578, named *PEPC2* herein) showed a much higher transcript level during the dark period than during the light period (Fig. 5b). We found that a duplicated pair of *K. fedtschenkoi PEPC2* genes (Kaladp0048s0578 and Kaladp0011s0355) clustered together with a PEPC gene (PEQU_07008) from *P. equestris* (Supplementary Fig. 12). PEQU_07008 was recently reported as the CAM-type PEPC in *P. equestris*, and, like Kaladp0048s0578, this orchid *PEPC* gene also showed higher transcript abundance during the dark period than during the light period^37^.

**Fig. 6:**
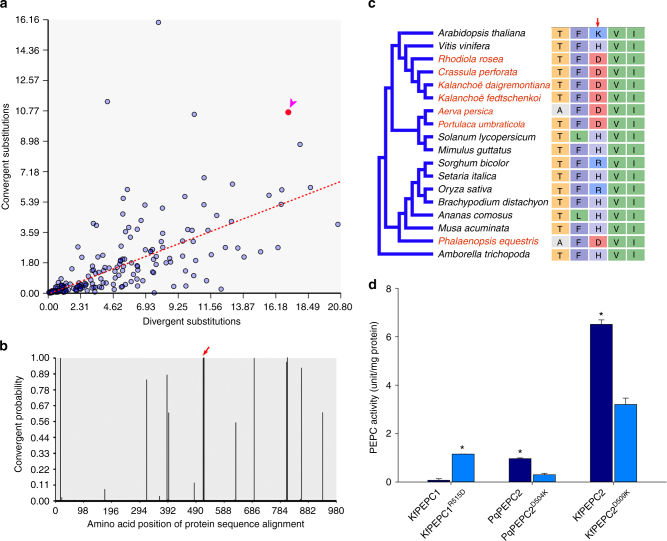
A convergent change in phosphoenolpyruvate carboxylase (PEPC) protein sequences in CAM species. **a** convergent- vs. divergent-substitutions in PEPC2 protein sequences between species listed in Supplementary Table 9. The arrow head indicates the comparison of *K. fedtschenkoi* vs. *P. equestris*. **b** Probability of convergent changes in PEPC2 protein sequence between *K. fedtschenkoi* and orchid. Red arrow indicates the protein sequence alignment site of convergent change (highlighted in red font at the alignment in panel c). **c** A convergent amino-acid change (from R/K/H to D) in PEPC2 shared by diverse species (highlighted in red font) at the alignment position indicated by the red arrow. **d** *In vitro* activity of PEPC isoforms in the absence of phosphorylation by PPCK. KfPEPC1: Kaladp0095s0055; KfPEPC1^R515D^: KfPEPC1 with mutation at residue 515 from arginine (R) to aspartic acid (D); KfPEPC2: Kaladp0048s0578.1; KfPEPC2^D509K^: KfPEPC2 with mutation at residue 509 from D to lysine (K); PqPEPC2: *P. equestris* PEPC gene PEQU07008; PqPEPC2^D504K^: PqPEPC2 with mutation at residue 504 from D to K. “*” indicates significant difference between wild-type and mutant of PEPC1 or PEPC2 (Student’s *t-*test; *P < *0.01). The error bars indicate standard deviation (SD) calculated from three replicates

**Fig. 7:**
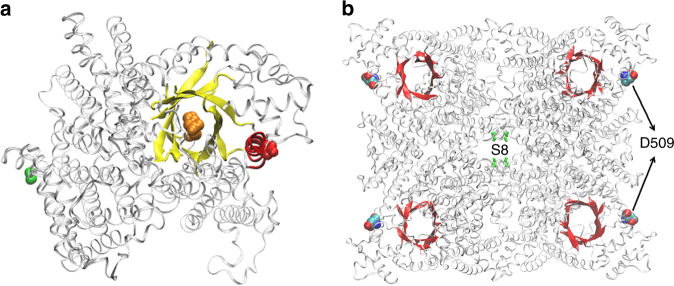
Protein structure model of phosphoenolpyruvate carboxylase 2 (PEPC2) in *Kalanchoë fedtschenkoi*. **a** PEPC2 (Kaladp0048s0578.1) structural model with a glucose-6-phosphate (G6P) substrate (orange spheres) bound at the β-barrel active site (yellow). D509 (red spheres) is located at an α-helix (red) in adjacent to the β-barrel and far from the hallmark serine residue (S8, green spheres) that is the phosphorylation target of PPCK1. **b** PEPC tetramer structure. The phosphorylation site (S8, green) is located at the interphase of the tetramer and D509 (spheres) is located at the peripheral of the tetramer. The β-barrel active site is shown in red, and no G6P activator may be required for activation of the PEPC activity following the competitive activating model of PEPC

Convergent changes in PEPC2 protein sequence were found between *K. fedtschenkoi* and *P. equestris* (Fig. 6a, b). Specifically, multiple protein sequence alignment revealed that an aspartic acid residue (D509) in Kaladp0048s0578 is conserved in PEQU_07008 and Kaladp0011s0355 (a duplicated copy of Kaladp0048s0578), but there was an arginine (R), lysine (K), or histidine (H) in the corresponding sites of the PEPC protein sequences of other tested species (Fig. 6c and Supplementary Fig. 12). The structural model of the Kaladp0048s0578 protein indicates that this single amino-acid substitution (from a basic amino-acid R/K/H to an acidic amino-acid D) is located in an α-helix adjacent to the active site in a β-barrel (Fig. 7a). We hypothesize that an activator binds to the active site of one subunit of the tetrameric complex of PEPC2, leading to allosteric conformational changes that subsequently activate another subunit of the tetramer (Fig. 7b). This model was supported by a recent crystallography structure of the *Flavaria trinervia* (a C_4_ photosynthesis plant) PEPC with an activator glucose-6-phosphate (G6P) bound at the β-barrel active center^38^. Based on this model, because D509 of PEPC2 (Kaladp0048s0578) is also negatively charged as G6P, the observed substitution may play a similar role as the activator by triggering allosteric conformational changes that lead to activation of the other subunits of PEPC tetramer. Nimmo^39^ reported that PEPC is subject to posttranslational regulation in the dark via phosphorylation by PPCK. *In vitro* analysis of the activities of different heterologously expressed PEPC isoforms showed that without phosphorylation by PPCK, PEPC1 from *K. fedtschenkoi* had a much lower activity than PEPC2 from either *K. fedtschenkoi* or *P. equestris* (Fig. 6d). Further, the R515D mutation significantly (Student’s *t*-test, *P < *0.01) increased the activity of *K. fedtschenkoi* PEPC1, whereas the D509K and D504K mutations significantly (Student’s *t*-test, *P < *0.01) reduced the activities of *K. fedtschenkoi* PEPC2 and *P. equestris* PEPC2, respectively (Fig. 6d). These results indicate that a single amino-acid mutation could significantly modify PEPC activity.

Our evolutionary analyses did not detect convergent evolution in either protein sequence or diel transcription patterns for the various decarboxylation genes that are expressed in *Kalanchoë* and *A. comosus*. In *Kalanchoë*, NAD(P)-ME genes were highly expressed, whereas the expression of the PEPCK gene was very low (Supplementary Fig. 18), consistent with the known high extractable activities of NAD-ME and NADP-ME in CAM leaves of *Kalanchoë* ^40,41^. By contrast, in *A. comosus* the transcript abundance of PEPCK was much higher than that of malic enzyme (ME) (Supplementary Fig. 18), supporting the model that malate decarboxylation in *Kalanchoë* is mediated by ME, which was recently substantiated using a transgenic RNAi approach^20,40^, whereas in pineapple a combination of MDH, working in the OAA-forming direction, coupled with PEPCK, converting OAA to PEP and CO_2_, are the candidate decarboxylation enzymes^18^, consistent with previous enzyme activity studies^8^.

### Convergent evolution of genes involved in stomatal movement

A unique feature of CAM physiology is the inverted light/dark pattern of stomatal movement relative to C_3_ photosynthesis, with stomata opening during the night in CAM and during the day in C_3_ photosynthesis plants^6^. Blue light is a key environmental signal that controls stomatal opening and phototropin 2 (PHOT2; AT5G58140), a blue light photoreceptor, mediates blue light regulation of stomatal opening in *Arabidopsis* ^42^. Twenty genes that could potentially be involved in stomatal movement in *K. fedtschenkoi* were predicted based on homology to *Arabidopsis* genes involved in the regulation of stomatal movement (Supplementary Table 11). One of these genes, Kaladp0033s0113, which encodes PHOT2, showed only a 1-h time shift in transcript abundance pattern relative to its *A. comosus* ortholog (Aco014242) (Supplementary Table 8), possibly indicating a convergent change in the diel pattern of its transcript abundance pattern in the two CAM species. In support of a convergent evolution hypothesis, the transcript abundance patterns of the two *PHOT2* genes in the CAM species showed 11- (*Kalanchoë*) and 9- (pineapple) hour phase shifts, respectively, relative to that of the *PHOT2* gene (AT5G58140) in the C_3_ photosynthesis species *Arabidopsis* (Fig. 4c). The timing of peak transcript abundance shifted from dawn in *Arabidopsis* to dusk in the two CAM species (Fig. 4c). This convergent change in diel transcript abundance pattern suggests that PHOT2 might contribute to the inverted day/night pattern of stomatal closure and opening in CAM species such that PHOT2 might function as a switch mediating the blue-light signal to open the stomata at dusk and the stomata could then remain open during the dark period.

### Convergent evolution of genes involved in heat tolerance

**Fig. 8:**
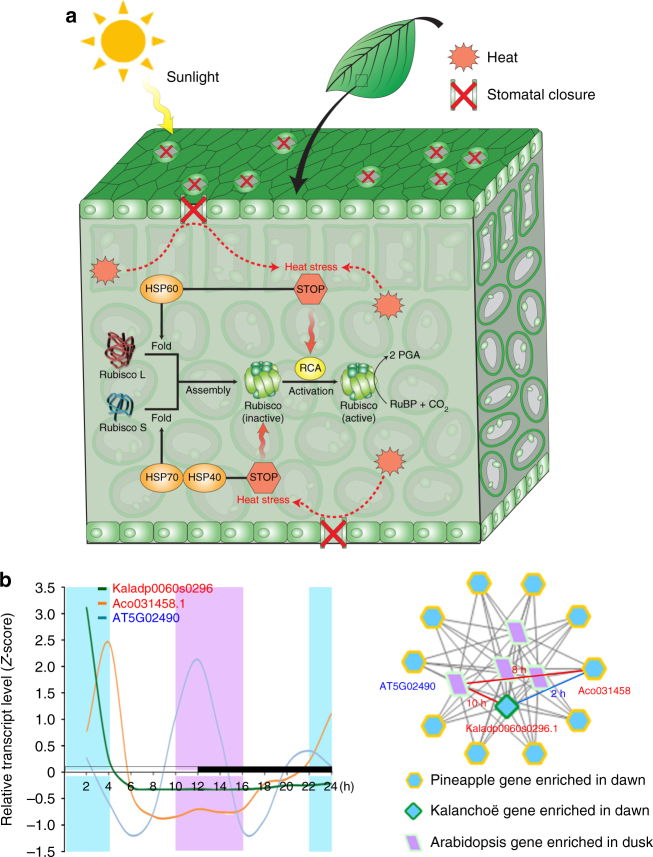
Convergent changes in diel transcript expression of heat-shock proteins (HSPs) in CAM species in comparison with C_3_ species. **a** Schematic representation of the possible roles of HSP40, HSP60, and HSP70 in leaf heat tolerance. **b** Comparison of diel transcript expression pattern of HSP70 between CAM species (*Kalanchoë fedtschenkoi* and pineapple) and C_3_ species (*Arabidopsis*). Left panel shows the diel transcript expression patterns. Right panel shows enrichment triangle network, in which a *K. fedtschenkoi* gene and a pineapple ortholog had significantly enriched expression in the same time-window, whereas an *Arabidopsis* ortholog had significantly enriched expression in the opposite time-window. The numbers are the time shifts in diel transcript expression pattern between genes connected by each edge. White and black bars indicate daytime (12-h) and nighttime (12-h), respectively. *X*-axis represents the time after the beginning of the light period. RuBisCO: Ribulose-1,5-bisphosphate carboxylase/oxygenase; RCA: rubisco activase; RuBP: ribulose-1,5-bisphosphate; PGA: 3-phosphoglycerate

The stomata of mature CAM leaves of *K. fedtschenkoi* close for the majority of the light period^40^, which may exacerbate the internal heat load on the leaves^43^. Photosynthesis is sensitive to heat stress and can be inhibited long before other symptoms of heat stress are detected^44^. Numerous studies have shown that the inhibition of photosynthesis by moderate heat stress is a consequence of RuBisCO deactivation, caused, in part, by the thermal instability of RuBisCO activase^45^. Heat-shock proteins can play a critical role in the stabilization of proteins under heat stress conditions^46^. Wang et al.^47^ reported that HSP40 (SlCDJ2) contributed to the maintenance of CO_2_ assimilation capacity mainly by protecting RuBisCO activity under heat stress and that HSP70 (cpHsp70) acted as a binding partner for SlCDJ2 in tomato. HSP70 can also function as nano-compartments in which single RbcL/RbcS subunits can fold in isolation, unimpaired by aggregation^48^, as illustrated in Fig. 8a. Among the HSP70 genes predicted in *K. fedtschenkoi*, Kaladp0060s0296 displayed peak transcript abundance in the morning, with only a 1-h shift in diel transcript abundance pattern relative to its *A. comosus* ortholog Aco031458, whereas these two HSP70 genes in the CAM species showed ~10-h shifts in diel transcript abundance pattern relative to their best-matched *A. thaliana* ortholog, AT5G02490 (Fig. 8b and Supplementary Table 8), suggesting that HSP70 has undergone convergent changes in diel transcript expression patterns during the evolution of CAM.

### Convergent evolution of genes in the circadian clock

**Fig. 9:**
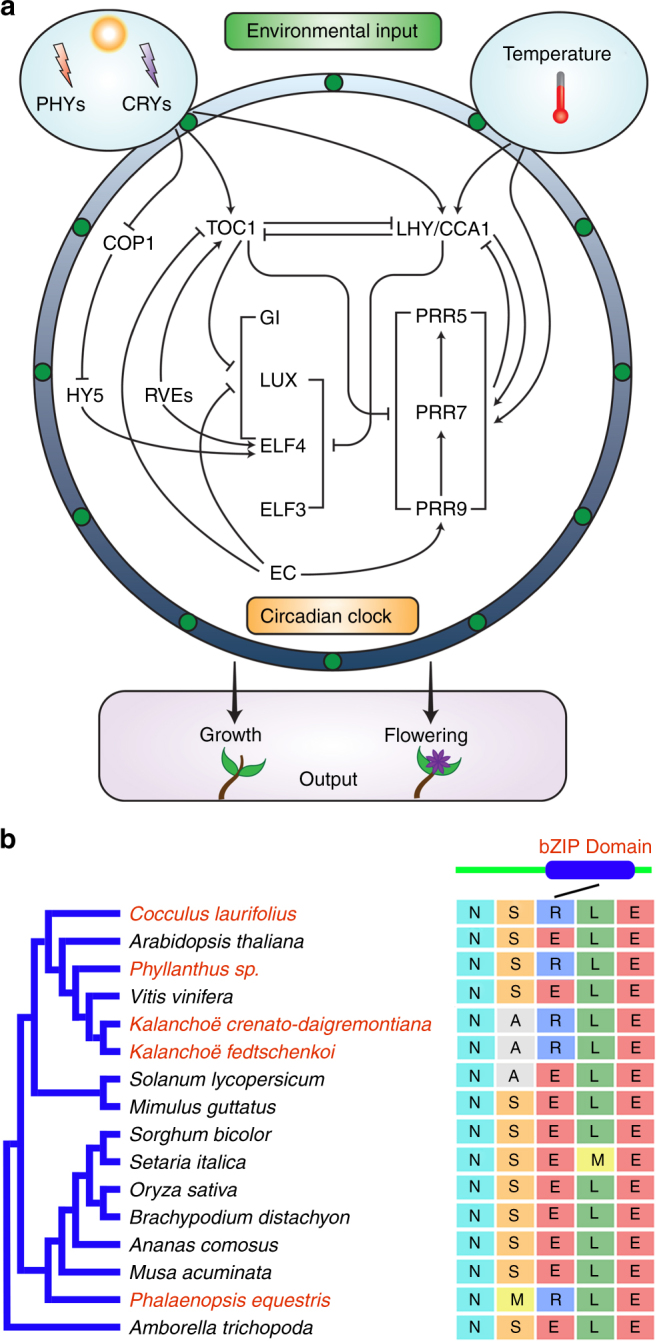
A convergent change in elongated hypocotyl 5 (HY5) protein sequences in CAM species. **a** An overview of the signaling pathway involved in circadian rhythm in plants. **b** Convergent change in HY5 protein sequences in diverse species (highlighted in red font). The black line indicates the protein sequence alignment position (located within the bZIP domain) where the mutation (E-to-R) occurred. CCA1 circadian clock associated 1, COP1 constitutive photomorphogenic 1, CRY cryptochrome, EC evening complex, ELF 3/4 early flowering 3/4, GI gigantea, LHY late elongated hypocotyl, LUX lux arrhythmo, PRR5/7/9 pinoresinol reductase 5/7/9, PHYs phytochromes, RVEs reveilles, TOC1 timing of cab expression 1

Key physiological and biochemical features of CAM including net CO_2_ exchange and PEPC phosphorylation are well established as outputs of the circadian clock, displaying robust oscillation under free-running constant conditions^20,40^. Thus, the circadian clock could be a key regulator of the diel reprogramming of metabolism and stomatal function that defines CAM. The molecular basis of circadian rhythms has been studied extensively in non-CAM species^33^. Based on homology to *Arabidopsis* genes that have been shown to play important roles as molecular components of the circadian clock, 35 *K. fedtschenkoi* genes were predicted to be involved in circadian rhythms (Supplementary Table 12). None of these *K. fedtschenkoi* genes are among the list of genes showing convergent changes in diel expression pattern (Supplementary Data 3), suggesting that CAM evolution did not involve major changes in the diel expression pattern of these known circadian rhythm genes shared between *Arabidopsis* and *K. fedtschenkoi*. However, we cannot rule out the possibility of convergent evolution in unknown circadian rhythm genes between these two species. Also, it is possible that genes that are not involved in circadian rhythms in *Arabidopsis* could have taken on this function in *K. fedtschenkoi*. On the other hand, Kaladp0060s0460, which encodes ELONGATED HYPOCOTYL5 (HY5), showed a convergent change in protein sequences between *K. fedtschenkoi* and *P. equestris* (Supplementary Table 10). HY5 is a bZIP family transcription factor in the blue light signaling pathway that acts as an input to entrain the circadian clock^33^ (Fig. 9a). A single amino-acid mutation (E-to-R) occurred in the C-terminal bZIP domains of the proteins encoded by Kaladp0060s0460 and its *P. equestris* ortholog PEQU_13446 as compared with HY5 from C_3_ or C_4_ photosynthesis species (Fig. 9b and Supplementary Fig. 14). The bZIP domain determines the DNA-binding ability of HY5 as a transcription factor^49^, mediating the interaction between HY5 and G-BOX BINDING FACTOR 1^50^. HY5 has been shown to move from shoot to root to coordinate aboveground plant carbon uptake in the leaf and belowground nitrogen acquisition in the root^51^. Therefore, the potential roles of HY5, Kaladp0060s0460, in circadian rhythmicity and shoot-to-root communication in *K. fedtschenkoi* needs to be investigated using experimental approaches such as loss-of-function mutagenesis^52^.

### Convergent evolution of genes in carbohydrate metabolism

Nocturnal production of phosphoenolpyruvate (PEP) as a substrate for dark CO_2_ uptake represents a substantial sink for carbohydrates in CAM plants, which has to be balanced with the provision of carbohydrates for growth and maintenance^53^. Carbohydrate active enzymes (CAZymes) play critical roles in regulating carbohydrate synthesis, metabolism, and transport in living organisms. There are six CAZyme classes: glycoside hydrolases (GHs), glycosyltransferases (GTs), polysaccharide lyases, carbohydrate esterases, auxiliary activities, and carbohydrate-binding modules. Each of these classes contains from a dozen to over one hundred different protein families based on sequence similarity^54^. The six classes of CAZymes have different functions. For example, GH enzymes catalyze the hydrolysis of glycosidic bonds, while GT enzymes catalyze the formation of glycosidic bonds. Using CAZyme domain-specific hidden Markov models, defined in the dbCAN database^55^, we identified 100 CAZyme families, including 1093 genes in the *K. fedtschenkoi* genome, comparable to the total number (1149) of CAZyme genes in *A. thaliana* (Supplementary Data 4 and 5). Among these CAZyme genes, four ortholog groups (ORTHOMCL68, ORTHOMCL93, ORTHOMCL207, and ORTHOMCL9830) of genes (e.g., Kaladp0550s0020, Kaladp0011s0363, Kaladp0037s0421, Kaladp0055s0317, respectively) belonging to the CAZyme families GH100, GT20, GT2, and GT5, respectively, displayed convergent changes in their patterns of diel transcript abundance in two CAM species (*K. fedtschenkoi* and *A. comosus*) compared with the C_3_ photosynthesis species (*A. thaliana*) (Supplementary Data 3). Specifically, the *K. fedtschenkoi* CAZyme genes with convergent changes in diel transcript abundance pattern (e.g., Kaladp0550s0020 [GH100], Kaladp0011s0363 [GH20], Kaladp0037s0421 [GT2], and Kaladp0055s0317 [GT5]) showed higher transcript abundance in the dark and early light period (Supplementary Fig. 19). In particular, two genes (Kaladp0011s0363 and Kaladp0055s0317) were predicted to be involved in starch and sucrose metabolism (Supplementary Fig. 20). Kaladp0011s0363 encodes a probable trehalose phosphate synthase. Trehalose 6-phosphate is an important sugar signaling metabolite and is thought to link starch degradation to demand for sucrose and growth^56^. Kaladp0550s0020 encodes an alkaline-neutral invertase that catalyzes the hydrolysis of sucrose to glucose and fructose. This invertase has also been implicated in metabolic signaling processes as an important regulator of plant growth and development^57^. Taken together, these data suggest that the evolution of CAM from C_3_ photosynthesis requires re-scheduling of the transcription of metabolic and signaling genes that regulate the partitioning of carbohydrates between reserves that provide substrates for CAM and carbohydrates required for growth.

In addition to the above convergent changes in expression pattern of four CAZyme genes, we also identified convergent changes in protein sequences of another two CAZyme genes (Kaladp0016s0058 [GT29] and Kaladp0067s0114 [GH35]) that were under positive selection (CodeML implemented in PosiGene^58^, *P < *0.05) in the dicot CAM species *K. fedtschenkoi* and one of the two monocot CAM species (*A. comosus* and *P. equestris*) (Supplementary Figs. 16–17). Kaladp0016s0058 encodes a putative sialyltransferase-like protein. Two single amino-acid mutations were found in Kaladp0016s0058 and its *A. comosus* ortholog Aco018360, as compared with the orthologous protein sequences of non-CAM species (Supplementary Fig. 16). These two mutations are close to each other (i.e., within a four-amino-acid distance), suggesting the possibility that the two mutations affect the same functional domain. Kaladp0067s0114 encodes a beta-galactosidase protein that hydrolyses the glycosidic bond between two or more carbohydrates. Two single amino-acid mutations were identified in Kaladp0067s0114 and its *P. equestris* ortholog PEQU_04899, as compared with the orthologous protein sequences of non-CAM species (Supplementary Fig. 17). These two mutations are close to each other (i.e., within an 11-amino-acid distance) in the middle of galactose-binding domain (Supplementary Fig. 17), which can bind to specific ligands and carbohydrate substrates for enzymatic catalytic reactions^59^. The relevance of these convergent changes in protein sequence to CAM evolution needs further investigation.

## Discussion

The CAM pathway has been found in 36 families of vascular plants^4^, among which Crassulaceae plays a unique role in CAM research because the pathway was first discovered in this succulent plant family and was thus named^60^. Within Crassulaceae, the genus *Kalanchoë* has been the most widely used for CAM research. As a model species for research into the molecular biology and functional genomics of CAM, *K. fedtschenkoi* stands out due to its relatively small genome, low repetitive sequence content, and efficient stable transformation protocols^20^. The genome sequence presented in this study renders *K. fedtschenkoi* as a new model for plant evolutionary and comparative genomics research, both for CAM photosynthesis and beyond. Although this study focused on genome-wide analysis of convergent evolution in CAM plants, the *K. fedtschenkoi* genome data can be used to facilitate CAM research related to: (1) generating loss-of-function mutants for functional characterization of CAM-related genes using genome-editing technology; (2) deciphering the regulation of CAM genes through identification of transcription factors and promoters of their target genes; (3) analyzing CAM gene expression by serving as a template for mapping of RNA sequencing reads and protein mass spectrometry data; and (4) identifying DNA polymorphisms related to genetic diversity of plants in the genus *Kalanchoë*.

Our genome-wide comparison of CAM species and non-CAM species revealed two types of convergent changes that could be informative with respect to the evolution of CAM: protein sequence convergence and convergent changes in the diel re-scheduling of transcript abundance. In the present study, a total of 60 genes exhibited convergent evolution in divergent eudicot and monocot CAM lineages. Specifically, we identified protein sequence convergence in six genes involved in nocturnal CO_2_ fixation, circadian rhythm, carbohydrate metabolism, and so on (Supplementary Table 10 and Supplementary Figs. 16–17). Also, we identified convergent diel expression changes in 54 genes that are involved in stomatal movement, heat stress response, carbohydrate metabolism, and so on (Supplementary Data 3). These results provide strong support for our hypothesis that convergent evolution in protein sequence or gene temporal expression underpins the multiple and independent emergences of CAM from C_3_ photosynthesis. New systems biology tools and genome-editing technologies^52,61^ offer great potential for plant functional genomics research based on loss- or gain-of-function mutants to characterize the role of the genes predicted here to have undergone convergent evolution.

Convergent gene function can arise by (1) a mutation or mutations in the same gene or genes that result in homoplasy in organisms or (2) independent causal mutation or mutations in different genes in each lineage^10,62^. We identified four genes that showed convergent changes in protein sequences, none of which were shared by the three CAM species *A. comosus*, *K. fedtschenkoi*, and *P. equestris* (Supplementary Table 10 and Supplementary Figs. 12–15), suggesting that CAM convergences result mainly from the second scenario. Alternatively, *K. fedtschenkoi* shares the convergent mutation in the PEPC2 protein sequence with *P. equestris* (Fig. 6), whereas it shares the convergent change in the pattern of diel transcript abundance of *PPCK1* with *A. comosus* (Fig. 4b). These results suggest that two alternative modes of convergent evolution could have occurred in pathways for nocturnal CO_2_ fixation. First, PPCK shifted from light period to dark period to promote the activation of PEPC1 (the most abundant isoform), as exemplified by *K. fedtschenkoi* and *A. comosus*. Second, a single amino-acid mutation from R/K/H to D to maintain the active state of PEPC2, without the need for phosphorylation, then occurred, as in *K. fedtschenkoi* and *P. equestris*.

According to the constrained selection theory of Morris^11^, we expected to see convergent changes in protein sequences in all the three CAM species. However, in this study, single-site mutations were found in only two of the three CAM species. Our additional positive selection analysis revealed that *Kalanchoë* did share convergent sequence mutation with the other two CAM species, but at alternate sites (Supplementary Figs. 16–17). This is consistent with a recent report showing that single amino-acid mutations were not shared by all the bird species that displayed convergent evolution of hemoglobin function as an adaptation to high-altitude environments^14^. Alternatively, our results, to some extent, support the contingent adaptation theory of Gould^15^. The relevance of these predicted convergent changes to CAM needs to be investigated using experimental approaches, such as transferring the convergent CAM genes to C_3_ photosynthesis species to test the effect of these genes on C_3_-to-CAM photosynthesis transition.

In this study, we did not identify any gene that exhibited both convergent changes in transcript abundance patterns (Supplementary Data 3) and convergent changes in protein sequence (Supplementary Table 10 and Supplementary Figs. 16–17), suggesting that convergent evolution of a gene in CAM species was achieved through either protein sequence convergence or rewiring of gene expression. Indeed, we have not seen any reports showing that both protein convergence and convergent gene expression change occurred in the same gene in any type of organisms to date. Thus, we can hypothesize that convergent evolution follows the “law of parsimony” that emphasizes the fewest possible assumptions for explaining a thing or event^63^. An implication of this hypothesis is that reuse of the key genes via altered diel expression patterns would be the shortest path for C_3_-to-CAM photosynthesis evolution; and on the other hand, mutations in some key sites of protein sequences, while keeping the temporal gene expression pattern unchanged, would be the shortest path for evolving new protein function required by CAM. Although our data fit this hypothesis, additional screens for genes that have convergent changes in both protein sequence and expression pattern in the future are merited.

Increasing human population and changes in global temperature and precipitation are creating major challenges for the sustainable supply of food, fiber, and fuel in the years to come. As a proven mechanism for increasing WUE in plants, CAM offers great potential for meeting these challenges. Engineering of CAM-into-C_3_ photosynthesis plants could be a viable strategy to improve WUE in non-CAM crops for food and biomass production^4,6^. The genes predicted here to have undergone convergent evolution during the emergence of CAM are crucial candidates for CAM-into-C_3_ photosynthesis engineering. Our results suggest that CAM-into-C_3_ photosynthesis engineering requires rewiring of the diel transcript abundance patterns for most of the candidate genes in the target C_3_ photosynthesis species, along with amino-acid mutations in the protein sequences of several other candidate genes. Specifically, CAM-into-C_3_ photosynthesis engineering efforts should be focused on changing the temporal patterns of transcript expression of endogenous genes in the target C_3_ photosynthesis species corresponding to the *K. fedtschenkoi* genes listed in Supplementary Data 3. CRISPR/Cas9-based knock-in approach^52^ can be used to replace the original endogenous promoters of the target genes with temporal promoters that confer temporal expression patterns similar to those of their orthologous genes in the CAM species. For example, dark-inducible promoters such as Din10^64^ can be used to drive the expression of carboxylation gene modules during the nighttime and light-inducible promoters, such as GT1-GATA-NOS101^65^, can be used to drive the expression of decarboxylation gene modules during the daytime. To make the protein sequence changes needed for CAM-into-C_3_ photosynthesis engineering, transferring the *K. fedtschenkoi* genes listed in Supplementary Table 10 to target C_3_ photosynthesis species via the *Agrobacterium*-mediated transformation could provide a relatively straightforward path to an efficient engineered CAM pathway. Alternatively, one could mutate the amino acids shown in Supplementary Figs. 12–15 using a knock-in strategy with emerging genome-editing technology^52^.

In summary, this study provides an important model genome for studying plant comparative, functional, and evolutionary genomics, as well as significant advances in our understanding of CAM evolution. Our findings hold tremendous potential to accelerate the genetic improvement of crops for enhanced drought avoidance and sustainable production of food and bioenergy on marginal lands.

## Methods

### Plant material

*Kalanchoë fedtschenkoi* ‘M2’ plants were purchased from Mass Spectrum Botanicals (Tampa, FL, USA) (Supplementary Method 2).

### Estimation of DNA content

The DNA contents of young leaf tissue samples were analyzed using flow cytometry analysis service provided by Plant Cytometry Services (The Netherlands). The internal standard was *Vinca minor* (DNA = 1.51 pg/2 C = 1477 Mbp/2 C).

### Chromosome counting

Images were collected using an Olympus FluoView FV1000 confocal microscope (Center Valley, PA, USA) with a 60× objective. Images were sharpened using Adobe Photoshop and chromosomes were counted using ImageJ software (Supplementary Method 3).

### Illumina sequencing of genome

The genomic DNA libraries of *K. fedtschenkoi* were sequenced on a MiSeq instrument (Illumina, CA, USA) using MiSeq Reagent Kit v3 (600-cycle) (Illumina, CA, USA) (Supplementary Method 4).

### Transcriptome sequencing

In order to capture mRNA abundance changes responsive to diel conditions, samples were collected in triplicate from mature *K. fedtschenkoi* leaves (i.e., the fifth and sixth mature leaf pairs counting from the top) every 2 h over a 24 h time course under 12 h light/12 h dark photoperiod. Additional tissues were sampled in triplicate including roots, flowers, shoot tips plus young leaves, and stems at one time point, 4 h after the beginning of the light period (Supplementary Method 5).

### Genome assembly and improvement

The *K. fedtschenkoi* genome was initially assembled using platanus^66^ from 70X Illumina paired-end reads (2 × 300 bp reads; unamplified 540 bp whole-genome shotgun fragment library), and three mate-libraries (3 kb, 14X; 6 kb, 12X; 11 kb, 11X). Further genome scaffolding was performed using MeDuSa^67^ sequentially with the genome assemblies of *K. laxiflora* v1.1 (Phytozome), *Vitis vinifera* Genoscope.12X (Phytozome), and *Solanum tuberosum* v3.4 (Phytozome).

### Protein-coding gene annotation

The genome annotation for *K. fedtschenkoi* was performed using homology-based predictors facilitated with transcript assemblies (Supplementary Method 6).

### Construction of orthologous groups

The protein sequences of 26 plant species were selected for ortholog group construction (Supplementary Method 7).

### Construction of species phylogeny

The phylogeny of plant species was constructed from the protein sequences of 210 single-copy genes identified through analysis of orthologous groups (see “Construction of orthologous groups” section). The details for species phylogeny construction are described in Supplementary Method 8.

### Construction of protein tribes and phylogenetic analysis

The protein sequences used for ortholog analysis (see “Construction of orthologous groups”) were also clustered into tribes using TRIBE-MCL^68^, with a BLASTp E-value cutoff of 1e-5 and an inflation value of 5.0. Phylogenetic analysis of the protein tribes is described in Supplementary Method 9.

### Analysis of convergence in protein sequences in CAM species

The phylogenetic trees of protein tribes (see aforementioned “Construction of protein tribes and phylogenetic analysis”) were examined to identify the “CAM-convergence” clade, which was defined to contain genes from *K. fedtschenkoi* (dicot) and at least one of the two monocot CAM species (*A. comosus* and *P. equestris*) without any genes from C_3_ or C_4_ species. The rationale for defining the “CAM-convergence” clade is that the dicot CAM species *K. fedtschenkoi* should be separated from the monocot CAM species if there is no convergence between *Kalanchoë* and the monocot CAM species (Supplementary Method 10).

### Gene Ontology analysis and pathway annotation

Whole-genome gene ontology (GO) term annotation was performed using BLAST2GO^69,70^ with a BLASTP E-value hit filter of 1 × 10^−6^, an annotation cutoff value of 55, and GO weight of 5. The enrichment of GO biological process and pathway annotation are described in Supplementary Method 11.

### Analysis of carbohydrate active enzymes

The protein sequences were searched against the dbCAN database^55^ using HMMER3 (http://hmmer.org/). The HMMER search outputs were parsed to keep significant hits with E-value <1e-23 (calculated by HMMER) and coverage >0.2 (calculated on the HMM, which is equal to (end position - start position)/total length of HMM), as suggested by a large scale benchmark analysis^71^.

### Estimation of transcript abundance in *Kalanchoë*

The RNA-seq data in fastq format were mapped to the *K. fedtschenkoi* genome using TopHat2^72^. Transcript abundance in FPKM (Fragments Per Kilobase of transcript per Million mapped reads) was estimated using Cufflinks^73^. All mapped read counts of the transcripts were counted by using htseq-count, a subprogram of HTseq^74^.

### Co-expression network analysis in *Kalanchoë*

The expression data of 16 samples in triplicates were used for co-expression network analysis, which included time-course data (12 time points: 2, 4, 6, …, 24 h after the beginning of the light period) from mature leaf and one time point data (4 h after the beginning of the light period) from roots, flowers, stems, and shoot tips plus young leaves collected in triplicate from the *K. fedtschenkoi* plants grown under 12 h light/12 h dark photoperiod. The details for co-expression network analysis are described in Supplementary Method 12.

### Cluster analysis of gene expression in *Kalanchoë*

Count values for each RNA-seq library were used to calculate polynomial regressions across time (Supplementary Method 13).

### Comparative analysis of gene expression

The diurnal expression data with 4-h intervals for *Arabidopsis thaliana* were obtained from Mockler et al.^75^ and adjusted to 2-h interval time series by interpolation using the SRS1 cubic spline function (http://www.srs1software.com/). The diurnal expression data with 2-h intervals for *K. fedtschenkoi* was generated in this study. The diurnal expression data with 2-h intervals for *Ananas comosus* was obtained from Ming et al.^4^. The gene expression data were normalized by Z-score transformation. The hierarchical clustering of gene expression was performed for genes in each ortholog group using the Bioinformatics Toolbox in Matlab (Mathworks, Inc.) based on Spearman correlation (Supplementary Method 14).

### Genome synteny analysis

Pairwise genome alignments were performed between grape genome (Genoscope.12X; https://phytozome.jgi.doe.gov) and *K. fedtschenkoi* (Supplementary Method 15).

### Protein 3D structural simulation

The protein structural models were built using the iterative threading assembly refinement (I-TASSER, V4.3) structural modeling toolkit^76,77^.

### Gas chromatography-mass spectrometry metabolite profiling

For the major metabolites of *K. fedtschenkoi*, a total of 36 leaf samples (the 5th and 6th fully expanded leaf pairs counting from the top) were collected with three biological replicates sampled every 2-h for a 24-h diurnal cycle. Additionally, three biological replicate samples of stems, roots, shoot tips plus young leaves, and flowers were also collected (Supplementary Method 16).

### In vitro protein expression and analysis of enzyme activity

The PEPC proteins were expressed in bacterial BL21strains (Novagen BL21 (DE3) pLysS Singles), and purified via Glutathione Sepharose 4B beads (GE Healthcare Life Sciences, Pittsburgh, PA, USA). The protein quality was checked via western blot using anti-PEPC antibody (Agrisera, Sweden) and the PEPC activity was determined (Supplementary Method 17).

### Data availability

The Department of Energy (DOE) will provide public access to these results of federally sponsored research in accordance with the DOE Public Access Plan (http://energy.gov/downloads/doe-public-access-plan). The *K. fedtschenkoi* genome sequence and annotation are deposited in Phytozome (https://phytozome.jgi.doe.gov). The *K. fedtschenkoi* genome sequence is also deposited at NCBI GenBank (https://www.ncbi.nlm.nih.gov/genbank/) under the accession code NQLW00000000. The genome sequencing reads are deposited in NCBI Sequence Read Archive (SRA) (https://www.ncbi.nlm.nih.gov/sra) with the BioSample accession SAMN07509503, which is the combination of the five individual BioSamples: SAMN07453935, SAMN07453936, SAMN07453937, SAMN07453938, and SAMN07453939. The RNA-Seq reads are deposited in NCBI SRA with the BioSample accession codes SAMN07453940 - SAMN07453987. The metabolite data is deposited at MetaboLights (http://www.ebi.ac.uk/metabolights/) under the accession code MTBLS519.

## Electronic supplementary material

Supplementary Information

Description of Additional Supplementary Files

Supplementary Data 1

Supplementary Data 2

Supplementary Data 3

Supplementary Data 4

Supplementary Data 5
