# Supplementary material for: The Kalanchoë genome provides insights into convergent evolution and building blocks of crassulacean acid metabolism
Source: Nat Commun. 2017 Dec 1;8:1899. doi: 10.1038/s41467-017-01491-7 (PMC5711932; doi:10.1038/s41467-017-01491-7)
Supplement: Supplementary file 1 — Supplementary Information [file 41467_2017_1491_MOESM1_ESM.pdf]

Supplementary Figures

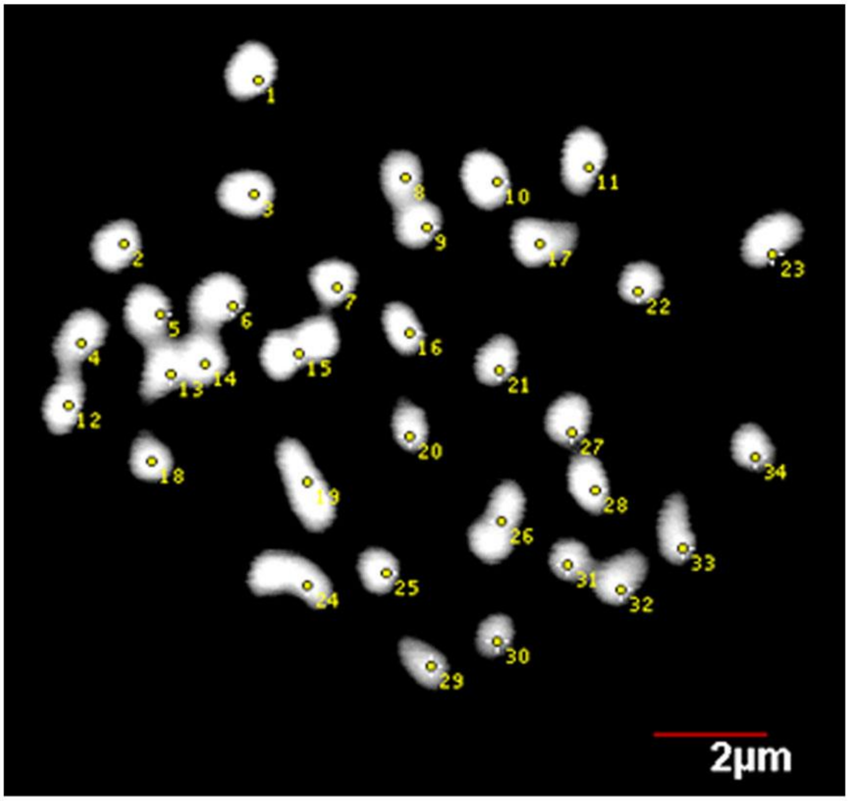

Supplementary Figure 1. Chromosome count of *Kalanchoë fedtschenkoi*.

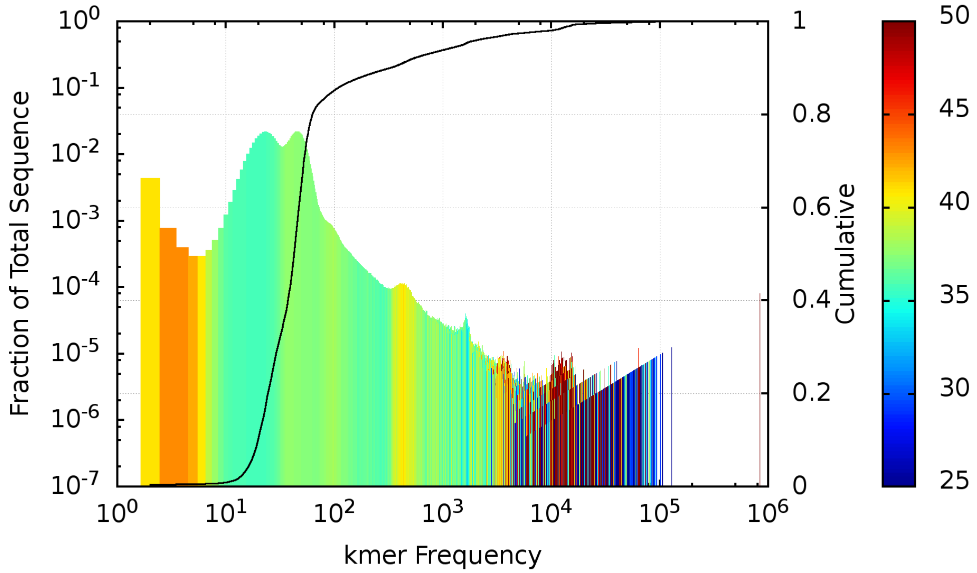

Supplementary Figure 2. Plot of the fraction of total sequence against 24-mer frequency for *Kalanchoë fedtschenkoi*. Each of the individual 24mer frequency bins are colored by the average GC for k-mers in that bin. The solid black line is the cumulative fraction of total sequence for the particular library on the secondary y-axis.

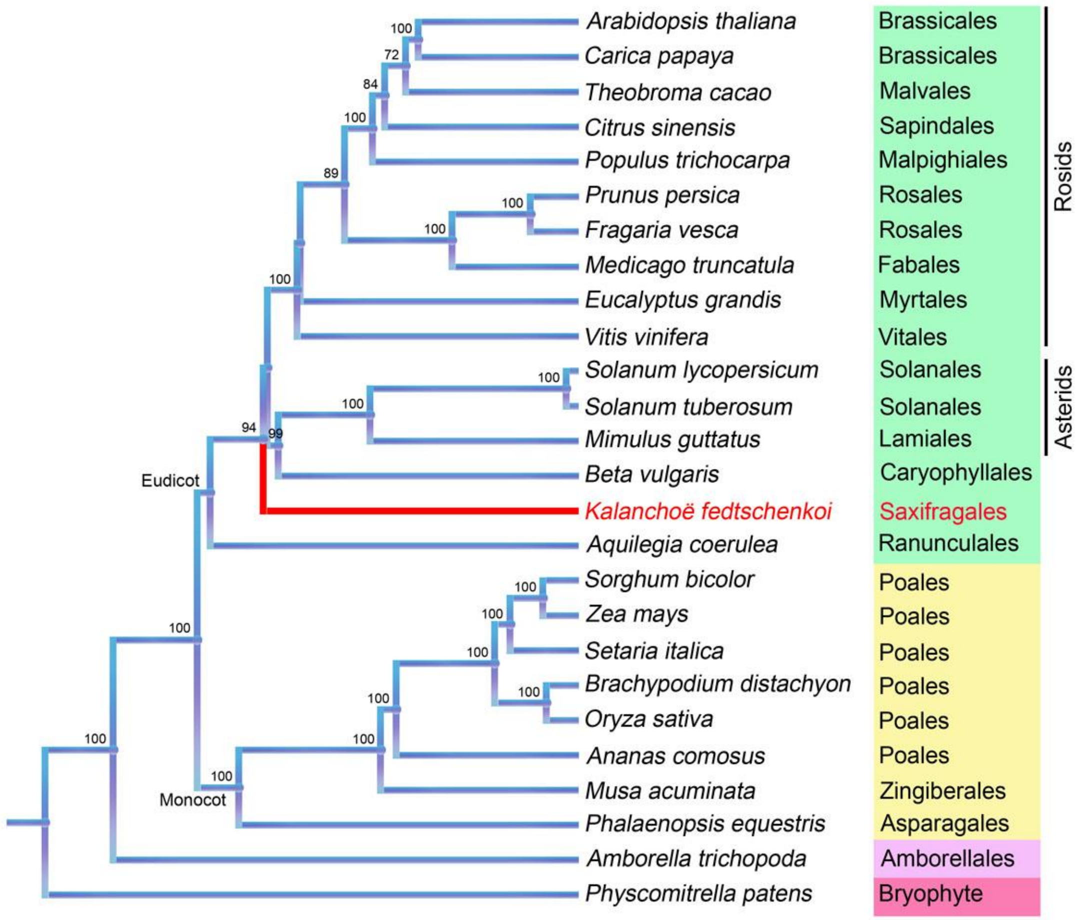

Supplementary Figure 3. A phylogenetic tree created from concatenated protein sequence alignment of 210 single-copy nuclear genes using maximum-likelihood method [1]. The numbers at each node are percent bootstrap support values from the maximum likelihood analysis.

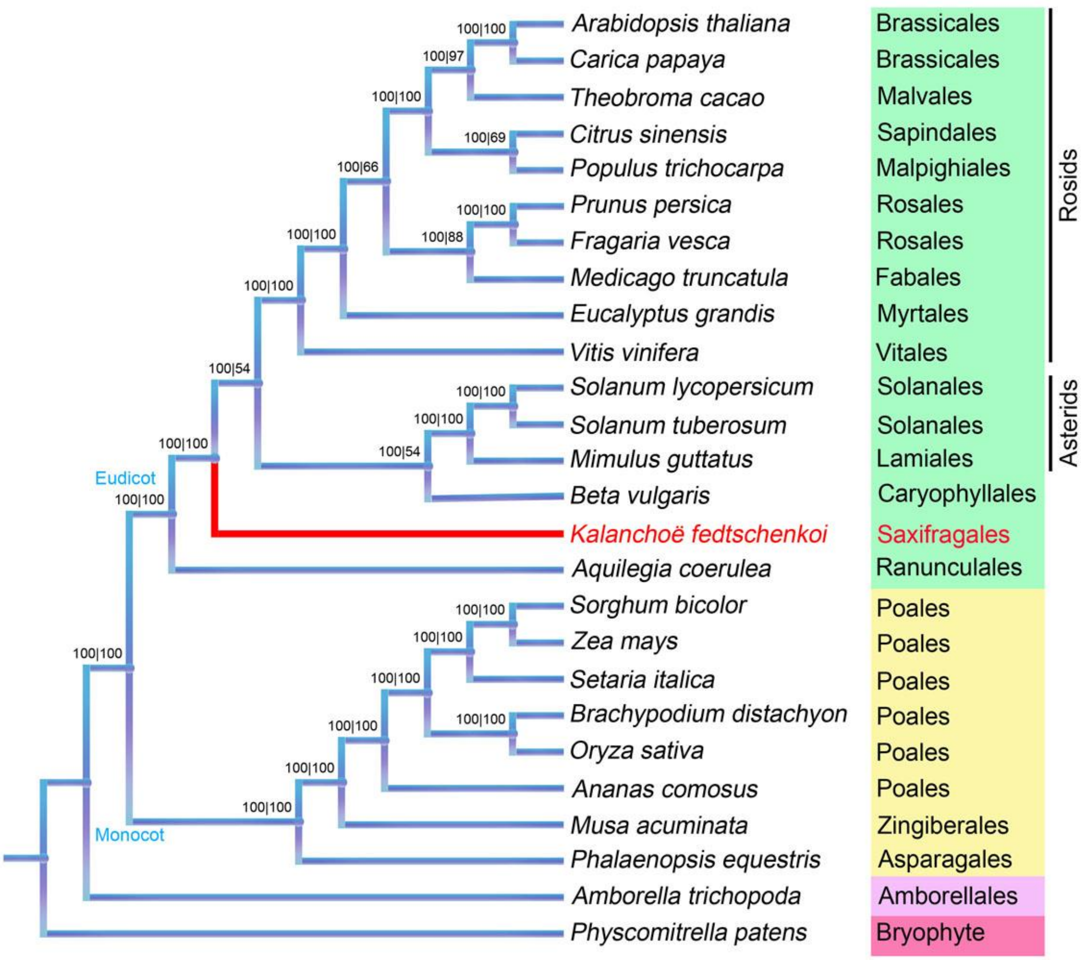

Supplementary Figure 4. Phylogenetic tree created from partitioned analysis of concatenated protein sequence alignment of 210 single-copy nuclear genes. The numbers on the left side of "|" are the percent probability from Bayesian inference using MrBayes [2]. The numbers on the right side of "|" are the bootstrap support values from maximum likelihood inference using IQ-TREE [1].

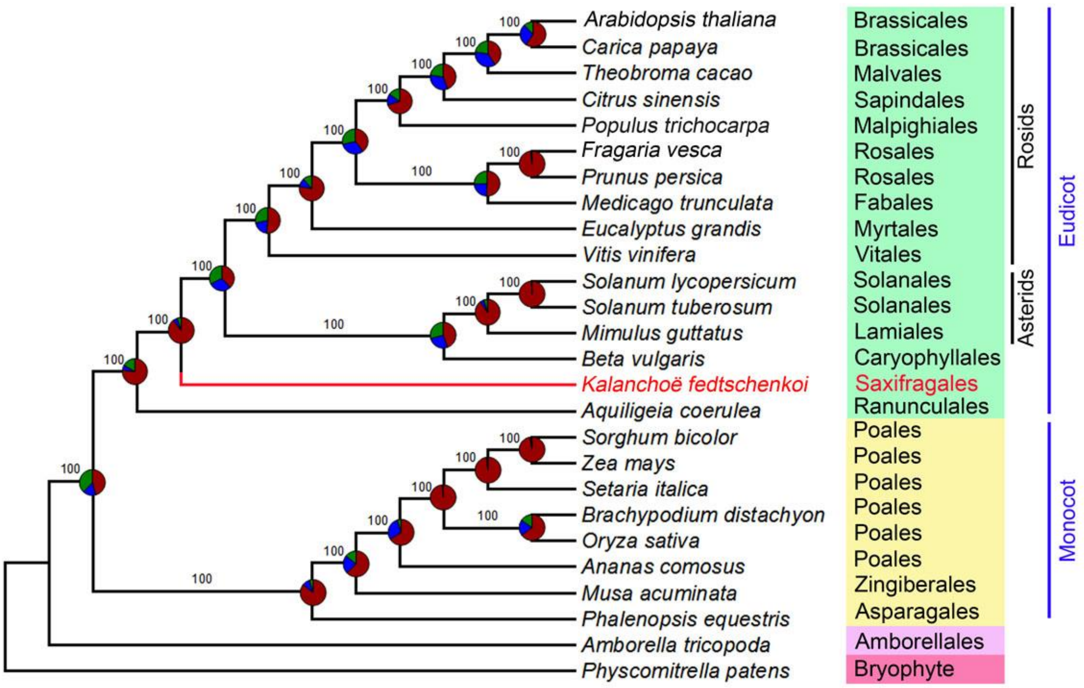

Supplementary Figure 5. A species tree reconstructed from 210 single copy genes using a fully Bayesian multispecies coalescent method. The coding-sequence (CDS) alignments for each of the 210 single-copy-gene ortholog groups were used to reconstruct the multispecies coalescent tree using starBEAST2 [3]. Pie graphs on nodes represent the proportion of gene trees that support the various quartets at every node, with red for the main topology shown in this tree, blue for the first alternative and green for the second alternative, respectively. Quartet frequencies displayed in pie graphs and the numbers representing percent posterior-probability at each node were calculated by ASTRAL-II [4].

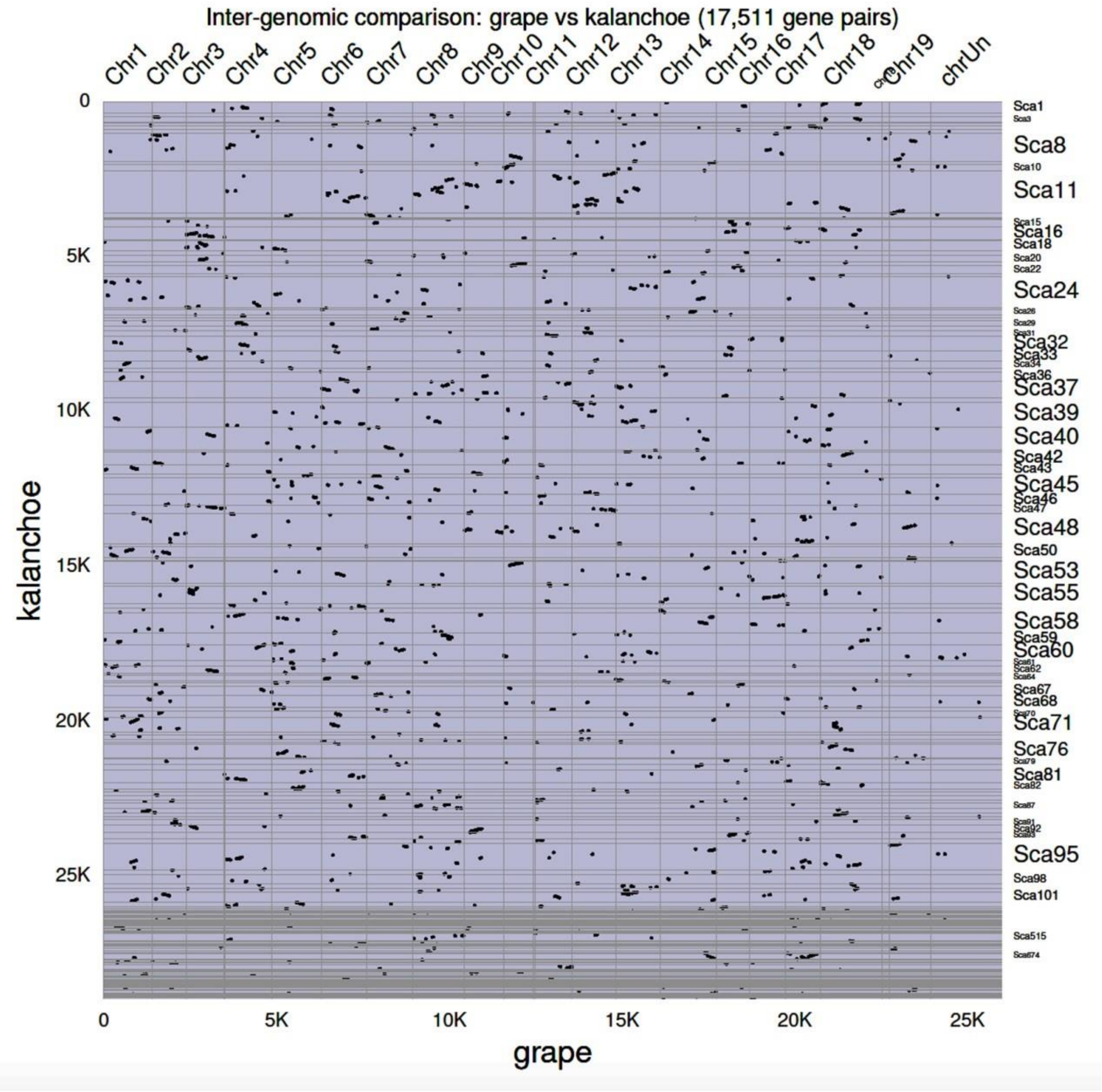

Supplementary Figure 6. Syntenic dot plot between the grape genome and the *Kalanchoë fedtschenkoi* genome. Each dot represents a homologous gene pair retained in a synteny block with at least four gene pairs per block.

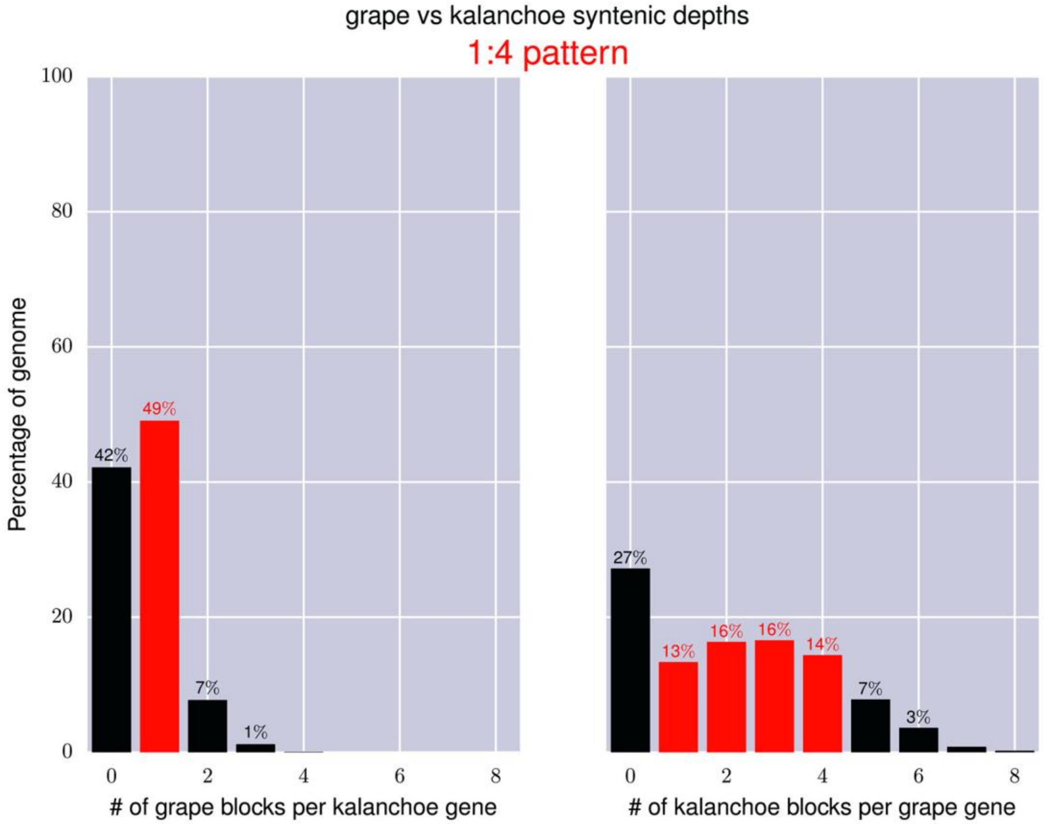

Supplementary Figure 7. Syntenic depths in grape vs. *Kalanchoë* genome comparison. The mode of each count distribution corresponds to the synteny patterns. From *Kalanchoë* point of view (left), we observed predominantly one single grape-*Kalanchoë* synteny block, suggesting no duplications in the grape lineage since the divergence; from grape point of view (right), we observed up to four grape-*Kalanchoë* synteny blocks, suggesting two whole-genome duplications (WGDs) in the *Kalanchoë* lineage since their divergence. We only included reciprocal best hits in order to remove orthologs due to shared WGDs.

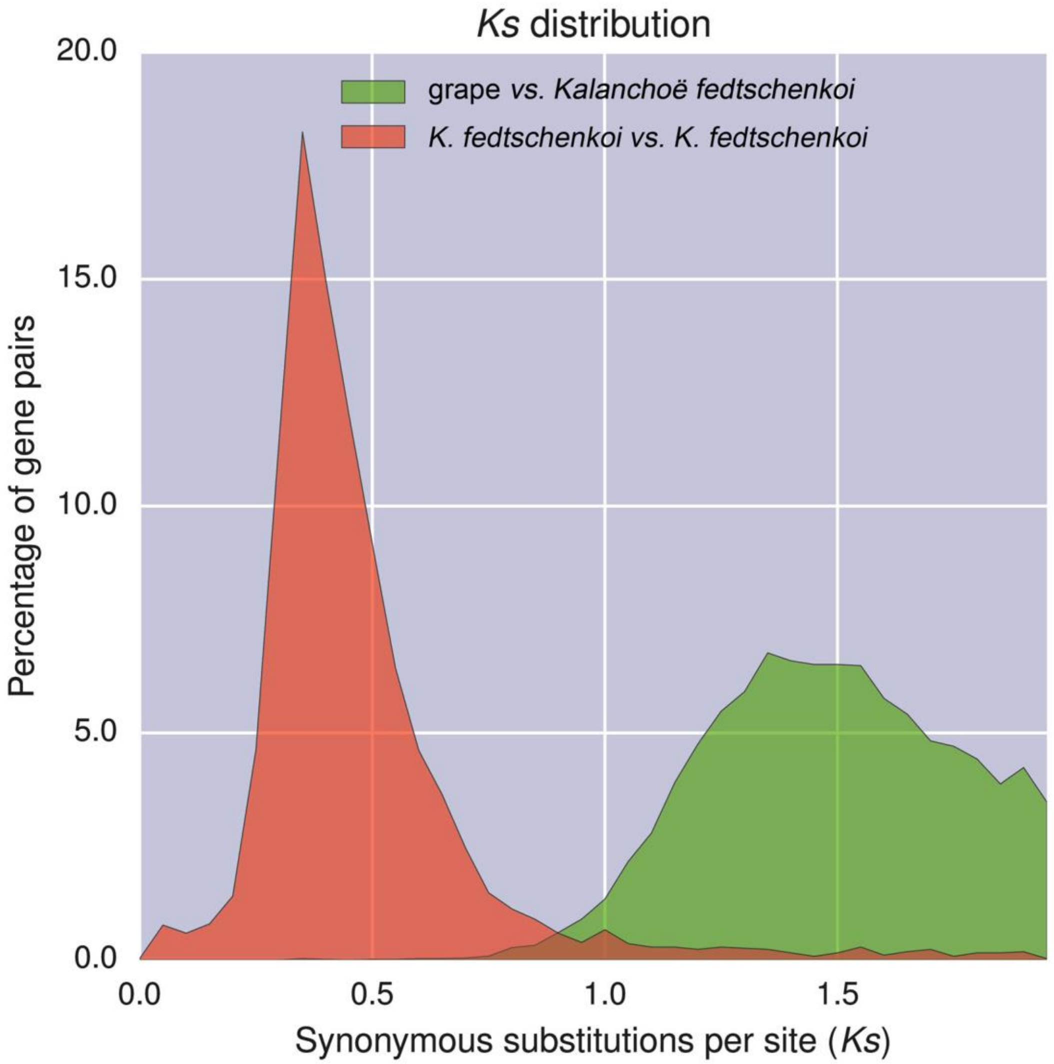

Supplementary Figure 8. Synonymous substitutions per synonymous site (*Ks*) distribution for syntenic gene pairs. *Ks* values were calculated per syntenic gene pair in grape-*Kalanchoë fedtschenkoi* and *K. fedtschenkoi*-*K. fedtschenkoi* comparisons.

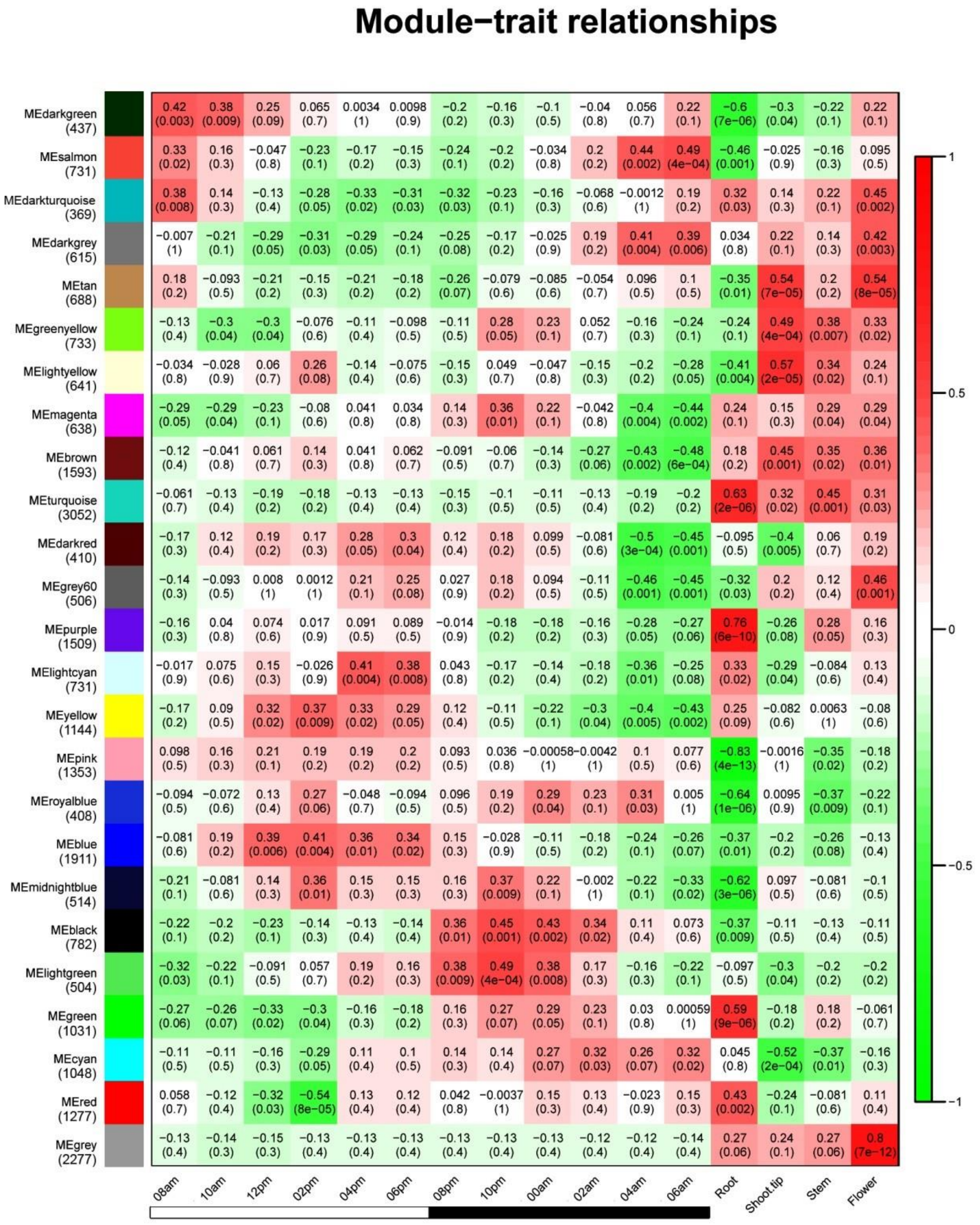

Supplementary Figure 9. The relationship between co-expression module and trait in *Kalanchoë fedtschenkoi*. The color blocks on the left side are the co-expression modules (named as MEdarkgreen, MEsalmon, etc.) constructed from RNA-seq data. The numbers underneath the module names are the number of genes in each module. The number represents the correlation between co-expression module and trait. The numbers in parentheses represent the *p*-value (Student's t-test) of the correlation. White and black bars indicate daytime (12-hour) and nighttime (12-hour), respectively.

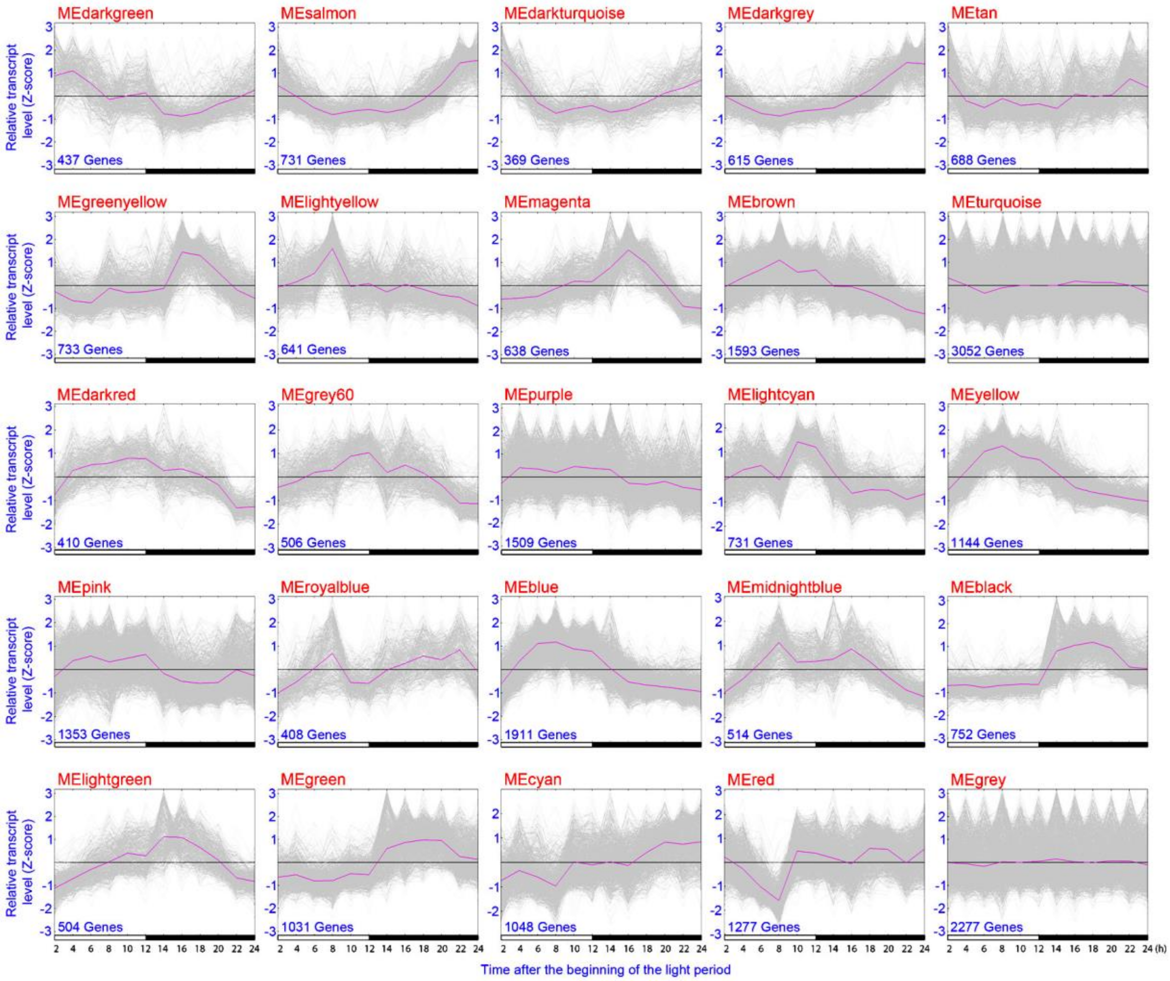

Supplementary Figure 10. Transcript changes of genes in each co-expression modules (Supplementary Fig. 9)**.** The grey lines in each module indicate expression pattern of individual gene in the modules. The purple line represents the median pattern of expression and total gene number was shown in individual module figures. White and black bars indicate daytime (12-hour) and nighttime (12-hour), respectively.

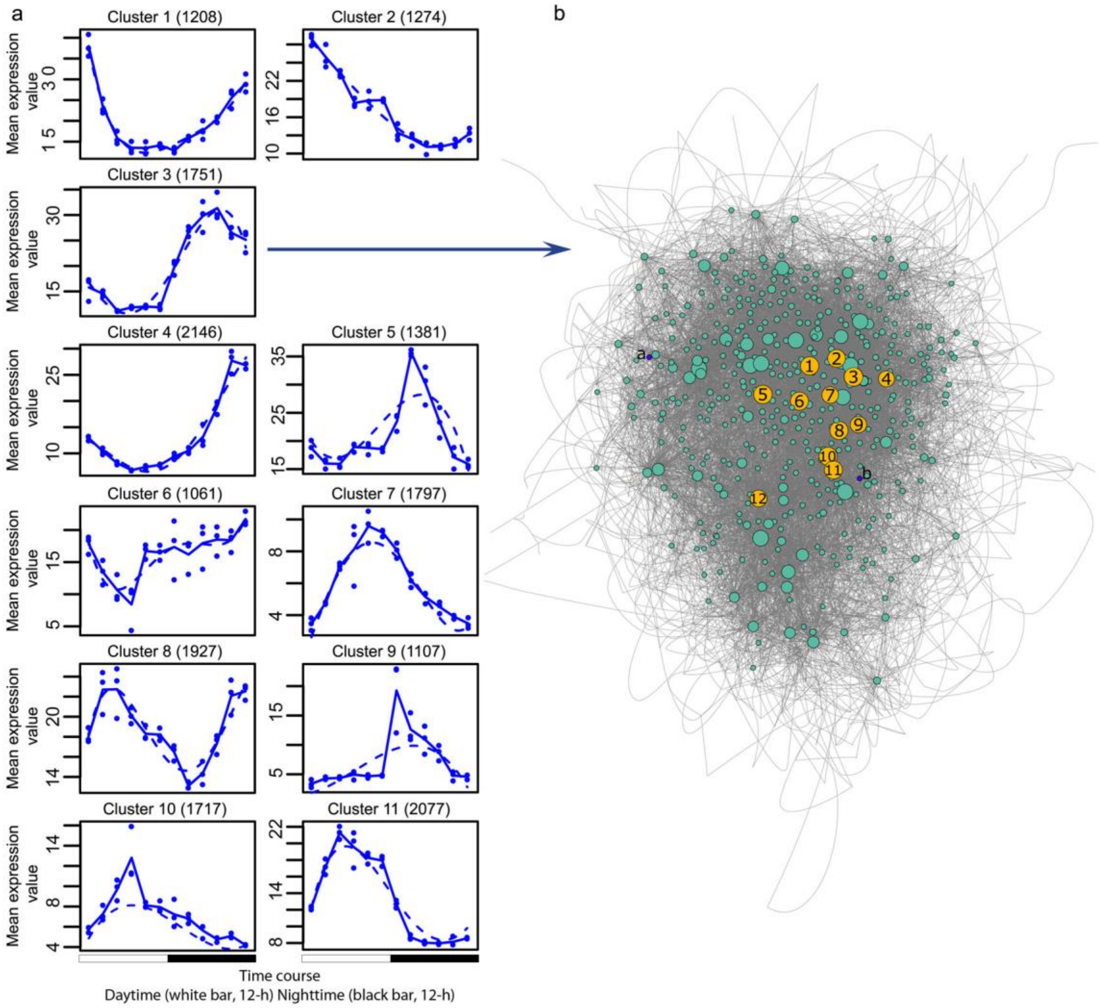

Supplementary Figure 11. Cluster analysis of diel expression of *Kalanchoë fedtschenkoi* genes that showed significant changes in transcript abundance over a 24-h period. (**A**) Diel transcript expression profile of the 11 gene clusters constructed, using maSigPro [5, 6], from the transcripts that showed significantly (ANOVA of glm models where H_0 = a flat line, P<0.05) "non-flat" diel expression patterns as determined by a polynomial regression. Dots are the median expression value for all genes in the cluster at a given time-point, the solid line is the median fit line across all time-points, and the dashed line is the cluster's polynomial fit. (**B**) A network constructed for Cluster 3 using ARACNE [7]. Node sizes are scaled by the number of directed connections, or edges. The top 1% of each network's nodes are highlighted and numbered, with annotations found in Supplementary Data 2. Number labels are not indicative of connectivity of the node. Nodes with less than 10 directed edges are not pictured. Blue colored nodes have putative CAM function, and are also annotated in Supplementary Data 2.

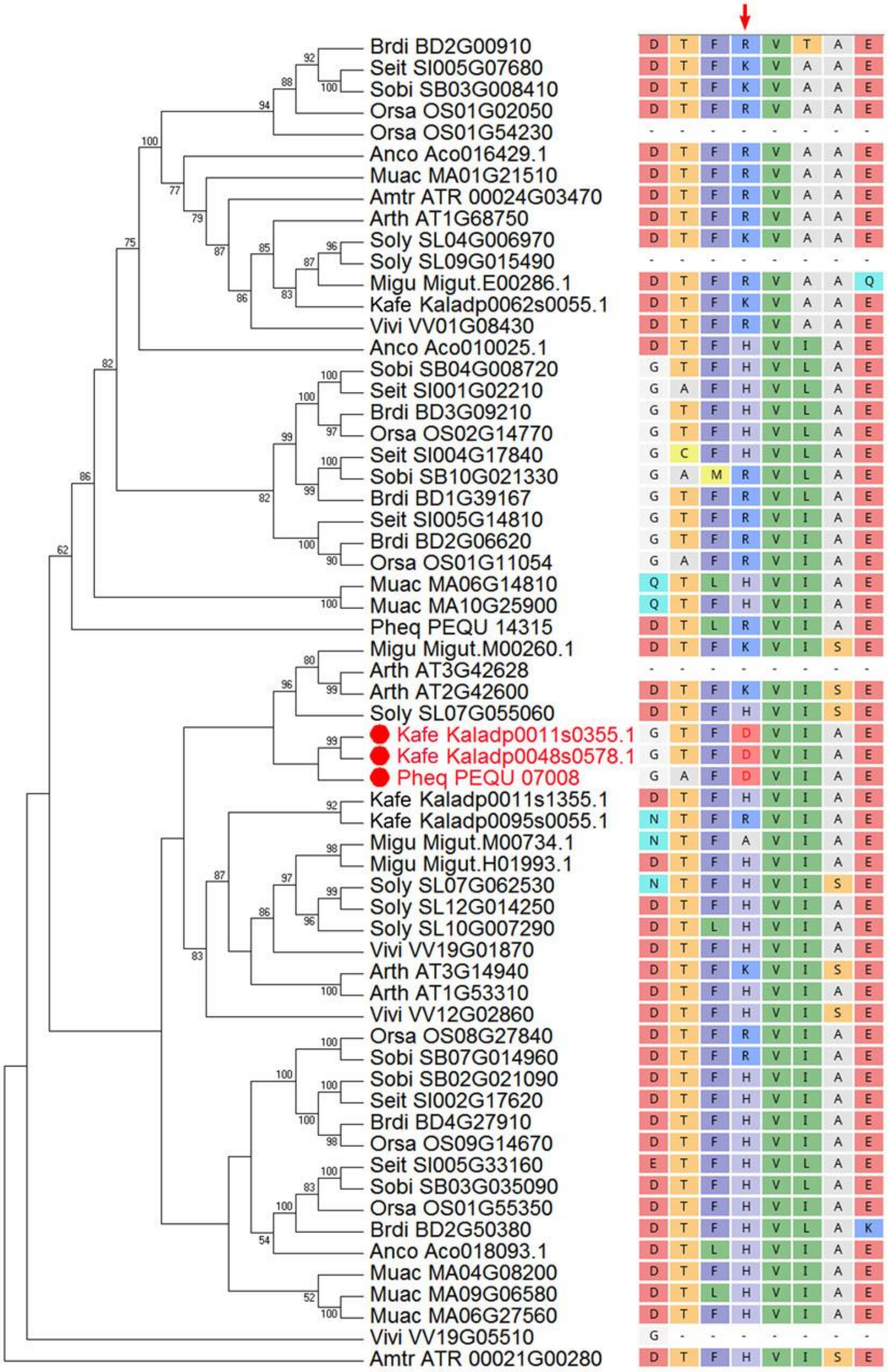

Supplementary Figure 12. A maximum-likelihood phylogeny of tribe I50_ F000807 that contains the gene encoding phospho*enol*pyruvate carboxylase (PEPC). The taxon names in the phylogenetic tree are listed as species abbreviation (the first four letters, see Supplementary Table 9) followed by gene/transcript name. Red dots highlight the genes showing convergent evolution in protein sequence. The red arrow indicates the protein sequence alignment position where the mutation (H/K/R-to-D) occurred.

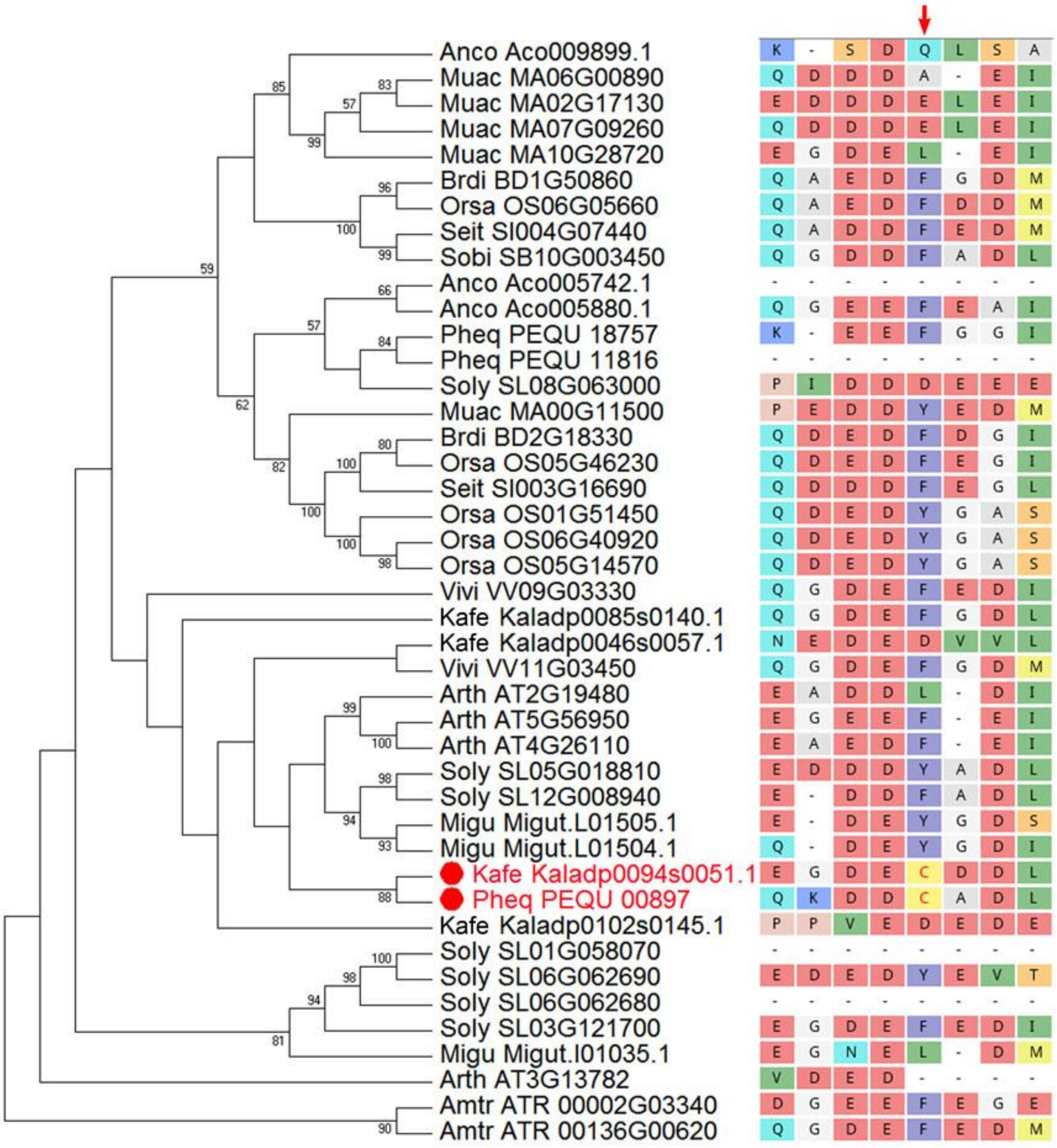

Supplementary Figure 13. A maximum-likelihood phylogeny of tribe I50_F001102 that contains the gene encoding nucleosome assembly protein 1 4-like (NAP1L4). The taxon names in the phylogenetic tree are listed as species abbreviation (the first four letters, see Supplementary Table 9) followed by gene/transcript name. Red dots highlight the genes showing convergent evolution in protein sequence. The red arrow indicates the protein sequence alignment position where the mutation (F/Y-to-C) occurred.

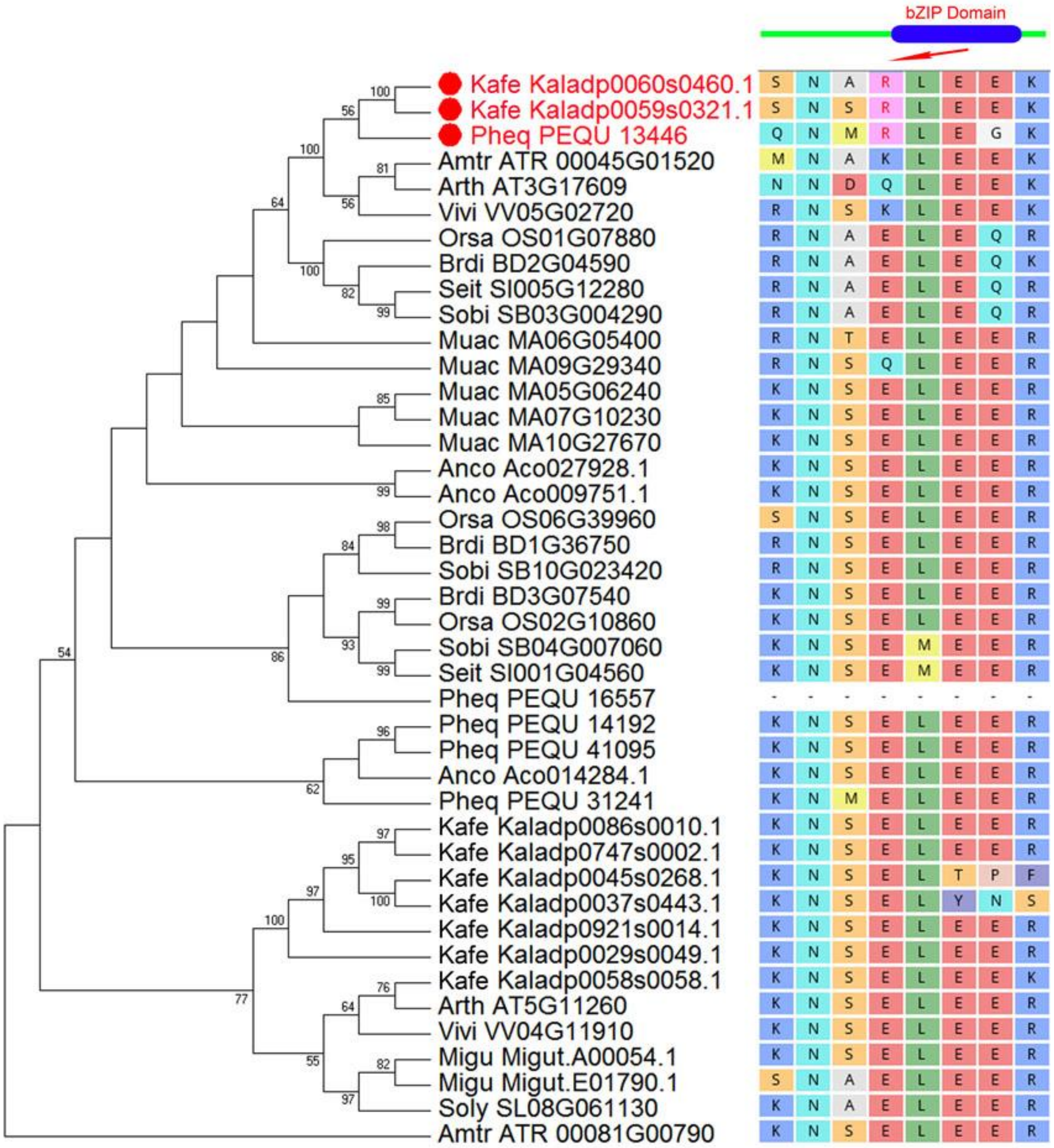

Supplementary Figure 14. A maximum-likelihood phylogeny of tribe I50_F001653 that contains the gene encoding transcription factor hy5-like protein (HY5). The taxon names in the phylogenetic tree are listed as species abbreviation (the first four letters, see Supplementary Table 9) followed by gene/transcript name. Red dots highlight the genes showing convergent evolution in protein sequence. The red arrow indicates the protein sequence alignment position (located within the bZIP domain) where the mutation (E/K/Q-to-R) occurred.

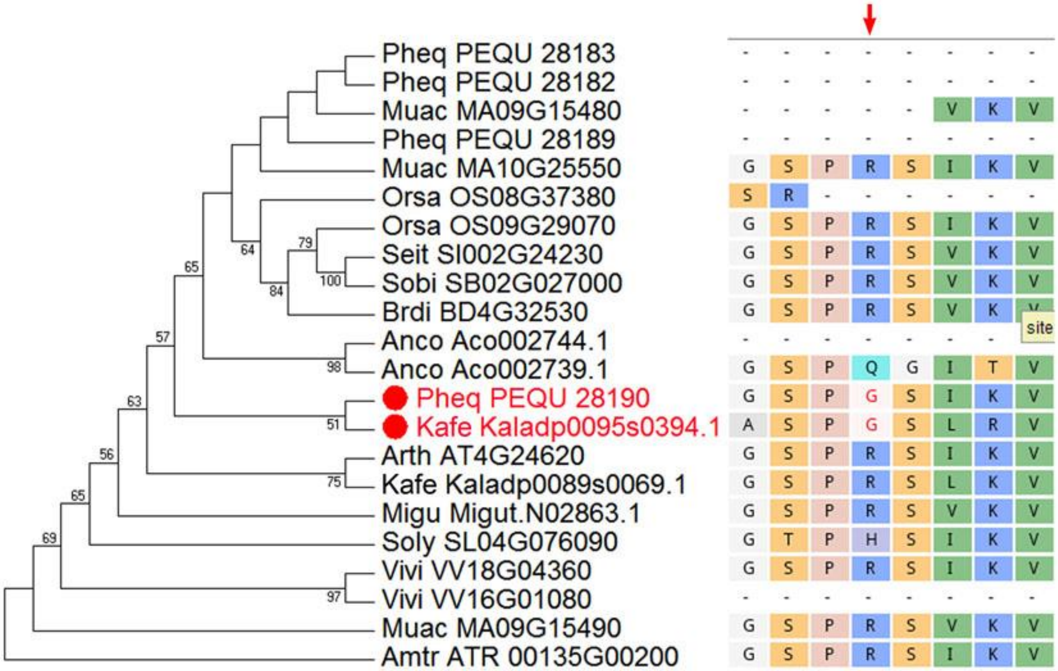

Supplementary Figure 15. A maximum-likelihood phylogeny of tribe I50_F003508 that contains the gene encoding a putative chloroplast-localized glucose-6-phosphate isomerase (GPI). The taxon names in the phylogenetic tree are listed as species abbreviation (the first four letters, see Supplementary Table 9) followed by gene/transcript name. Red dots highlight the genes showing convergent evolution in protein sequence. The red arrow indicates the protein sequence alignment where the mutation (H/Q/R-to-G) occurred.

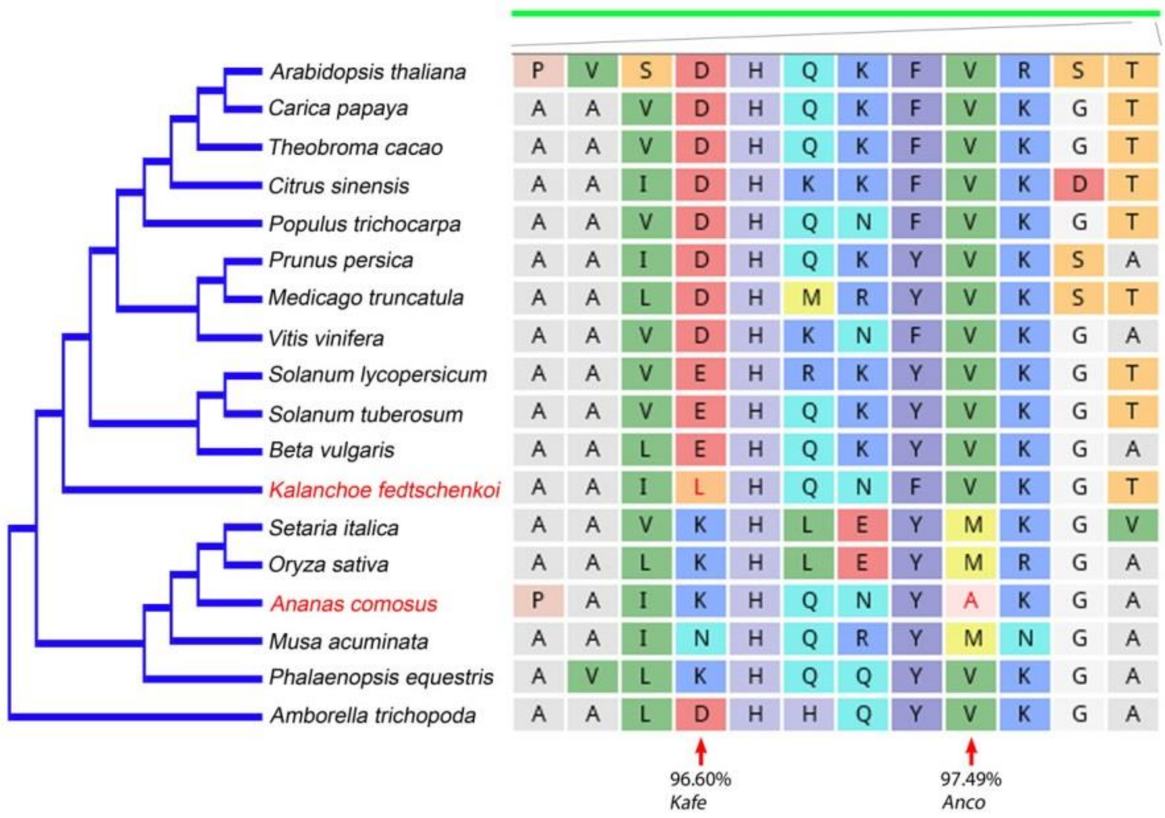

Supplementary Figure 16. A gene encoding a putative sialyltransferase-like protein (SIA2) under positive selection in *Kalanchoë fedtschenkoi* and *Ananas comosus*. Specifically**,** two genes, Kaladp0016s0058 in *K. fedtschenkoi* and Aco018360.1 in *A. comosus*, were indentified to be under positive selection (CodeML in implemented in PosiGene [8], *P*<0.05) in comparison with their orthologs in the non-CAM species listed in this figure. The CAM species that under positive selection were highlighted in red. The arrows indicate the specific mutation positions in individual CAM species and the percentage indicates the mutation probability in that position as compared to non-CAM species. "Kafe" is the abbreviation for *Kalanchoë fedtschenkoi* and "Anco" is the abbreviation for *Ananas comosus*.

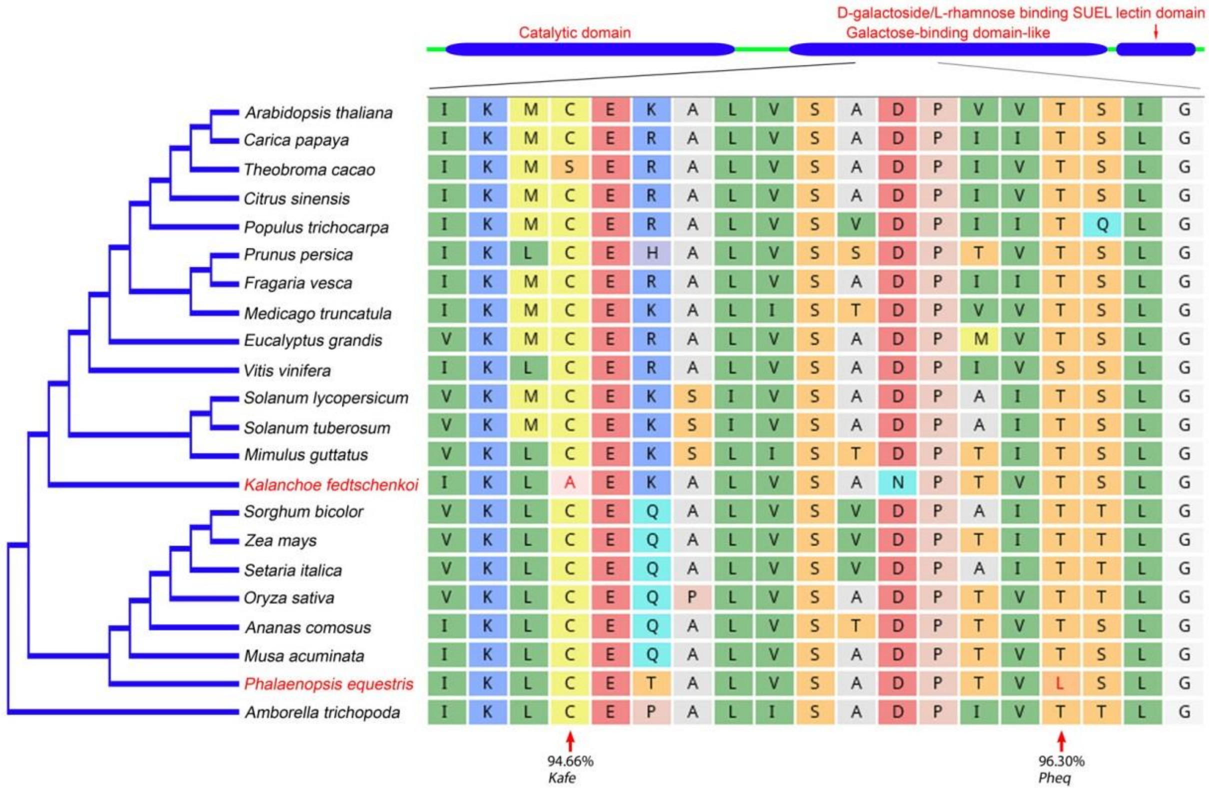

Supplementary Figure 17. A gene encoding a beta-galactosidase protein (BGAL3) under positive selection in *Kalanchoë fedtschenkoi* and *Phalaenopsis equestris*. Specifically**,** two genes, Kaladp0067s0114 in *K. fedtschenkoi* and PEQU_04899 in *P. equestris,* were identified to be under positive selection (CodeML in implemented in PosiGene [8], P<0.05) in comparison with their orthologs in the non-CAM species listed in this figure. The CAM species that under positive selection were highlighted in red. The arrows indicate the specific mutation positions in individual CAM species and the percentage indicates the mutation probability in that position as compared to non-CAM species. "Kafe" is the abbreviation for *Kalanchoë fedtschenkoi* and "Pheq" is the abbreviation for *Phalaenopsis equestris*.

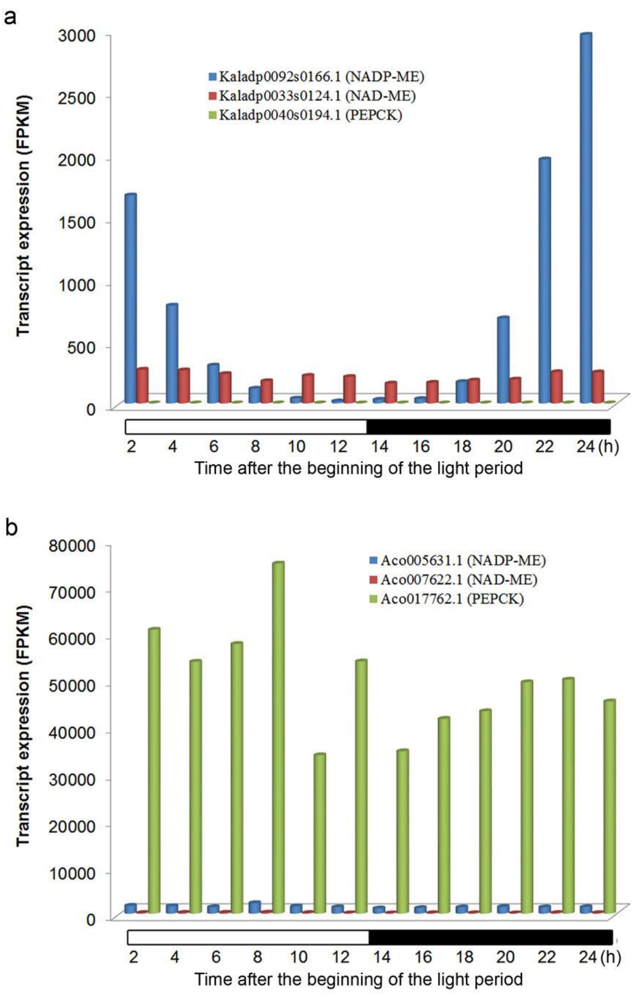

Supplementary Figure 18. Diel expression of genes involved in the decarboxylation process in *Kalanchoë fedtschenkoi* and pineapple. (a) Diel transcript expression of decarboxylation genes in *K. fedtschenkoi*. (**b**) Diel transcript expression of decarboxylation genes in pineapple. NAD-ME, NAD-malic enzyme; NADP-ME, NADP-malic enzyme; PEPCK, phospho*enol*pyruvate carboxykinase. White and black bars indicate daytime (12-hour) and nighttime (12-hour), respectively.

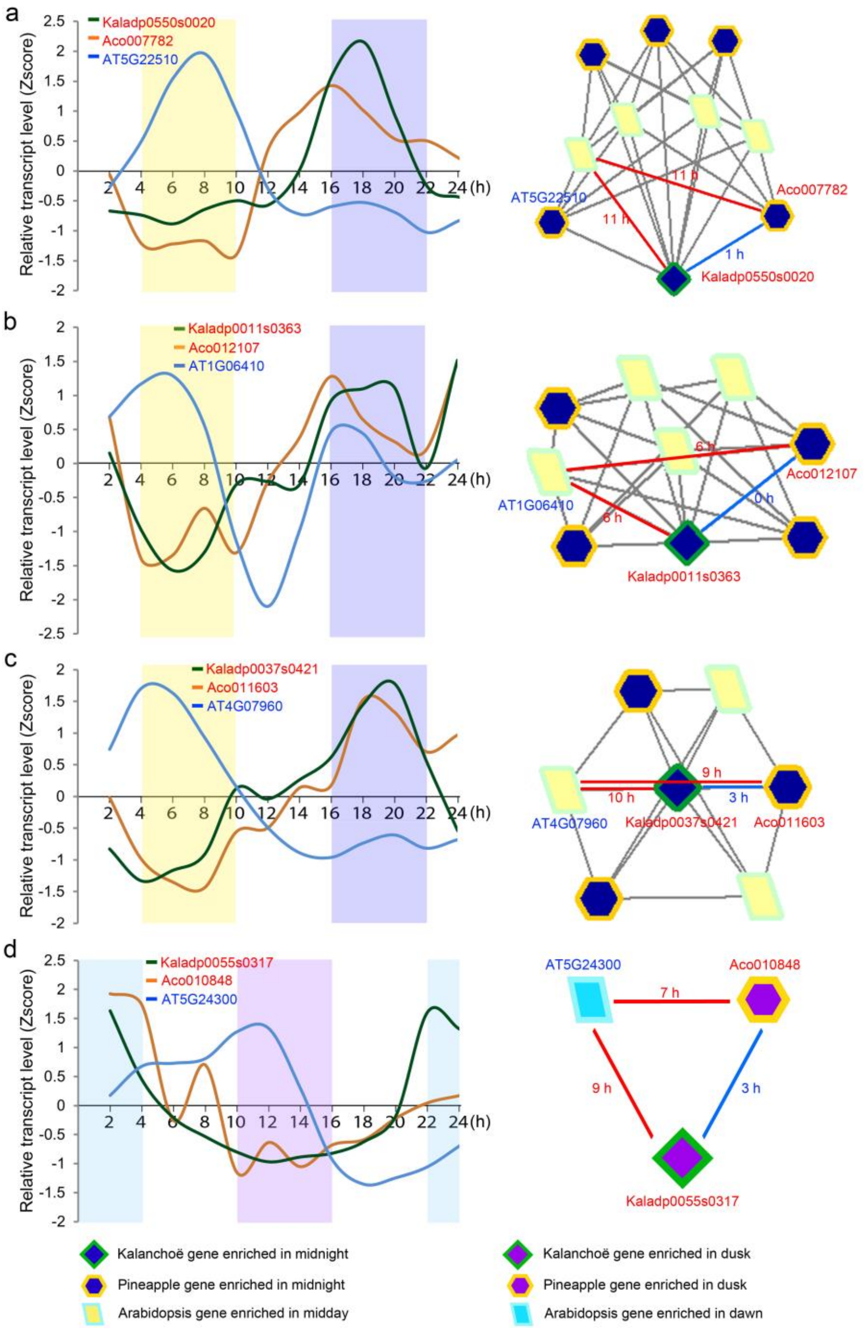

Supplementary Figure 19. Enrichment of triangle networks of genes relevant to CAZyme in *Kalanchoë fedtschenkoi*, pineapple and *Arabidopsis*. (**a**) Network of orthogroup (ORTHOMCL68) containing GH100 genes (CINV1). (**b**) Network of orthogroup (ORTHOMCL93) containing GT20 genes (TPS). (**c**) Network of orthogroup (ORTHOMCL207) containing GT2 genes (CSLC12). (**d**) Network of orthogroup (ORTHOMCL9830) containing GT5 genes (Starch synthase). White and black bars indicate daytime (12-hour) and nighttime (12-hour), respectively. X-axis represents the time after the beginning of the light period.

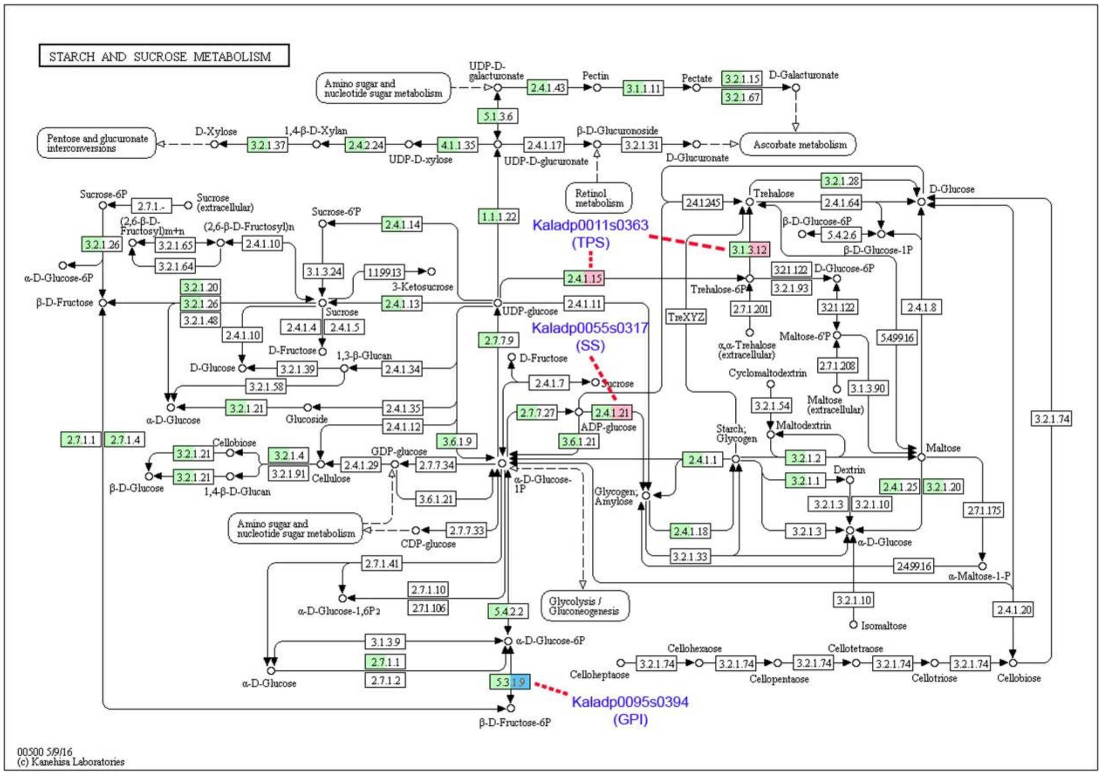

Supplementary Figure 20. An overview KEGG map of starch and sucrose metabolism. Green color indicates the genes identified in *Kalanchoë fedtschenkoi.* Pink color highlights the *K. fedtschenkoi* genes (Kaladp0011s0363 and Kaladp0055s0317) showing convergent evolution with *Ananas comosus* (pineapple) in diel expression pattern. Blue color highlights the *K. fedtschenkoi* gene (Kaladp0095s0394) showing convergent evolution in protein sequence with *Phalaenopsis equestris* (orchid). GPI: glucose-6-phosphate isomerase chloroplastic-like; SS: starch synthase chloroplastic amyloplastic-like; TPS: trehalose phosphate synthase.

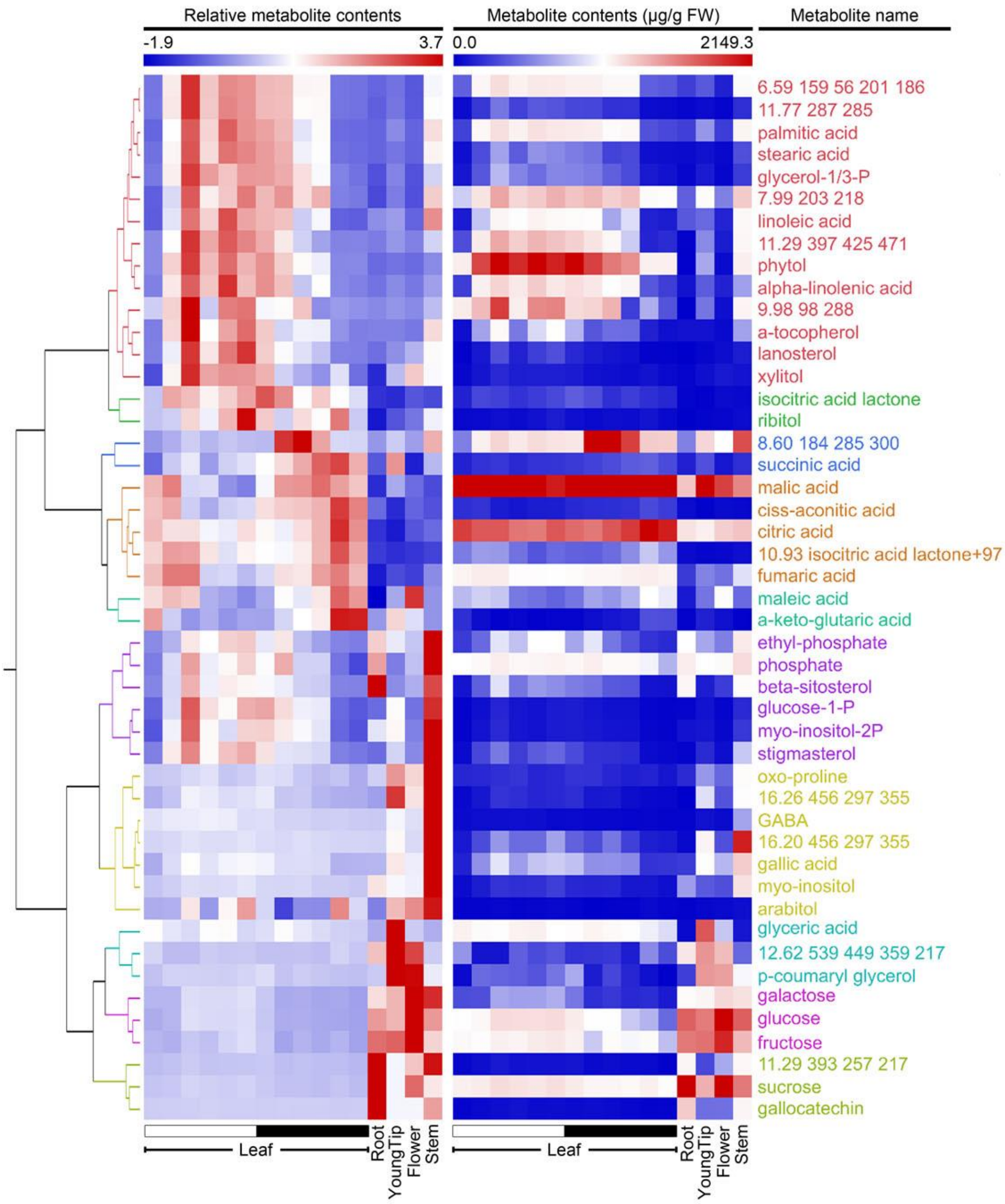

Supplementary Figure 21. A hierarchical clustering (Fast Ward method) heatmap highlights 47 quantified metabolite contents in *Kalanchoë fedtschenkoi*. The black and white bars indicate nighttime (12-hour) and daytime (12-hour), respectively. The numbers (e.g., "11.77 287 285", "9.98 98 288") on the right side indicate retention times of metabolites with unknown identity.

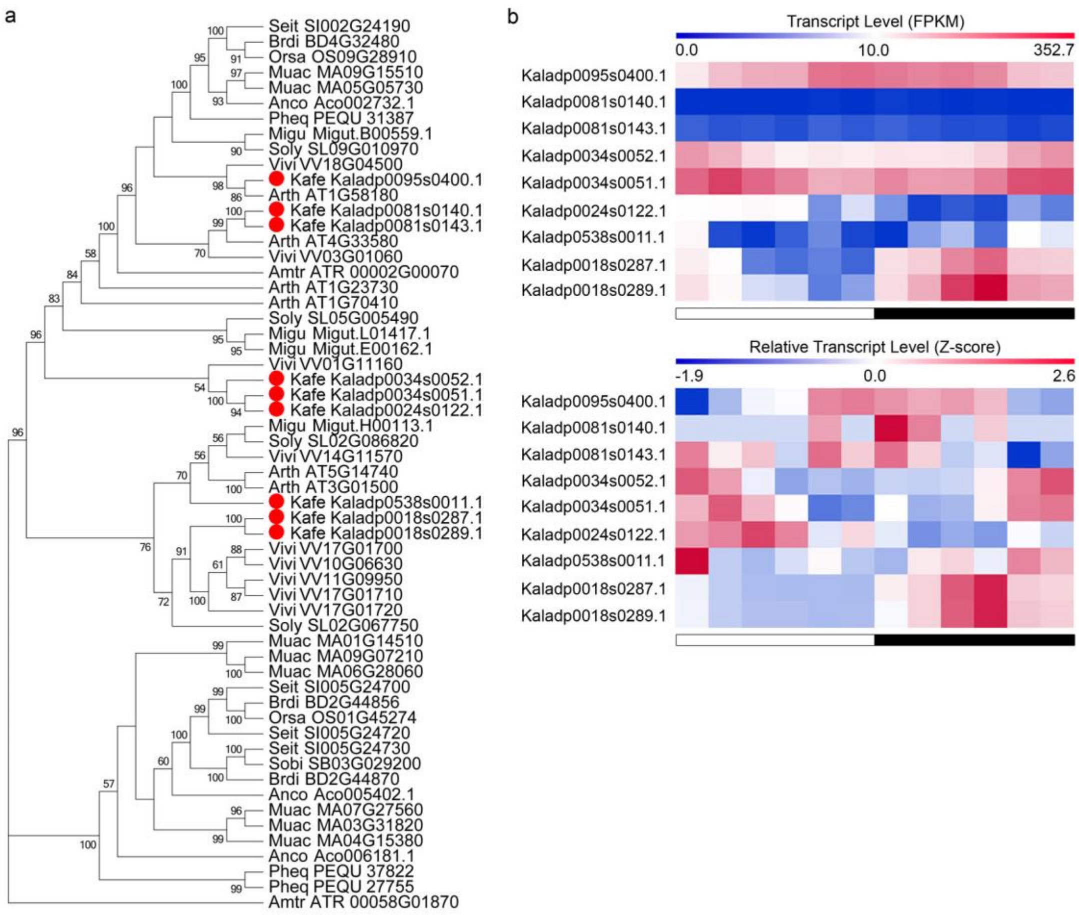

Supplementary Figure 22. Phylogeny of the beta-carbonic anhydrase (β-CA) proteins and diel transcript expression. (**a**) A maximum-likelihood phylogenetic tree constructed from protein sequences. (**b**) Diel transcript expression of the *β-CA* genes in *Kalanchoë fedtschenkoi*. The taxon names in the phylogenetic tree are listed as species abbreviation (the first four letters, see Supplementary Table 9) followed by gene/transcript name. White and black bars indicate daytime (12-hour) and nighttime (12-hour), respectively.

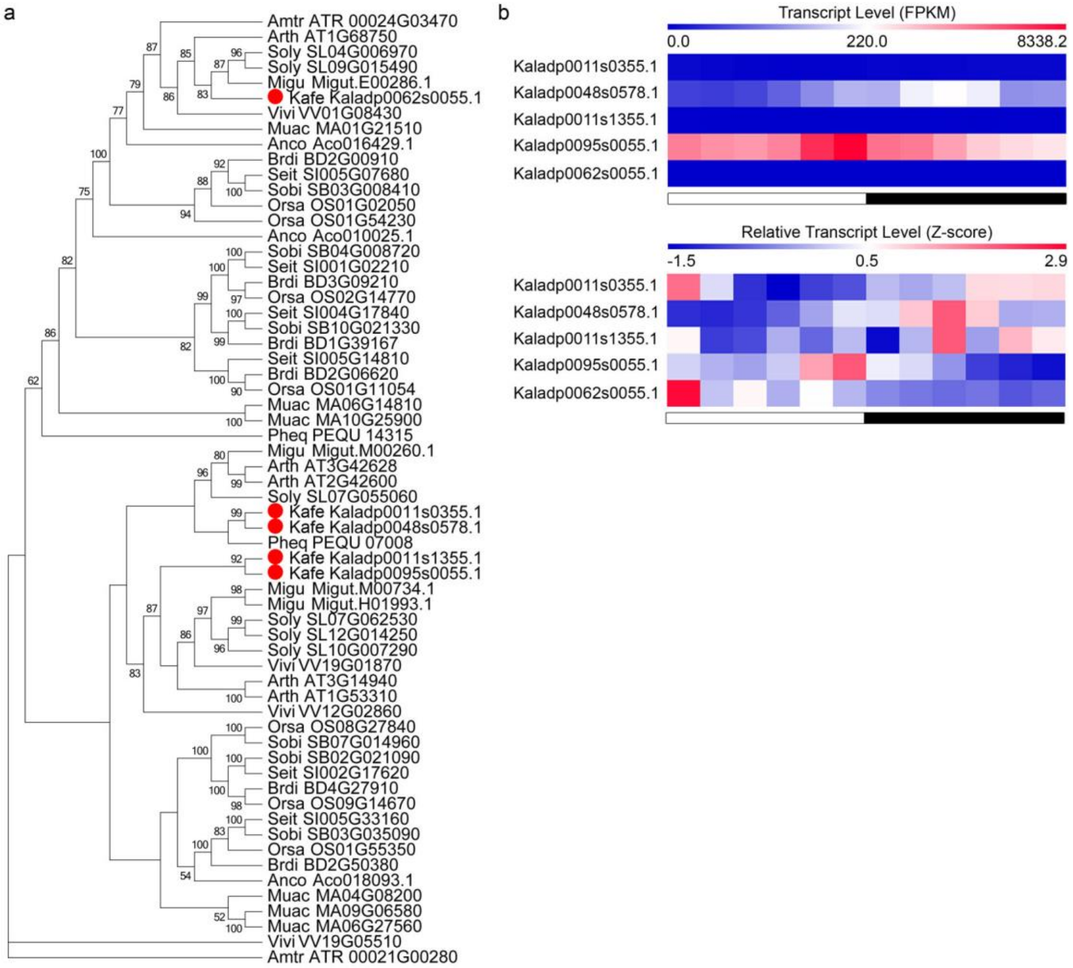

Supplementary Figure 23. Phylogeny of the phospho*enol*pyruvate carboxylase (PEPC) proteins and diel transcript expression. (**a**) A maximum-likelihood phylogenetic tree constructed from protein sequences. (**b**) Diel transcript expression of the *PEPC* genes in *Kalanchoë fedtschenkoi*. The taxon names in the phylogenetic tree are listed as species abbreviation (the first four letters, see Supplementary Table 9) followed by gene/transcript name. White and black bars indicate daytime (12-hour) and nighttime (12-hour), respectively.

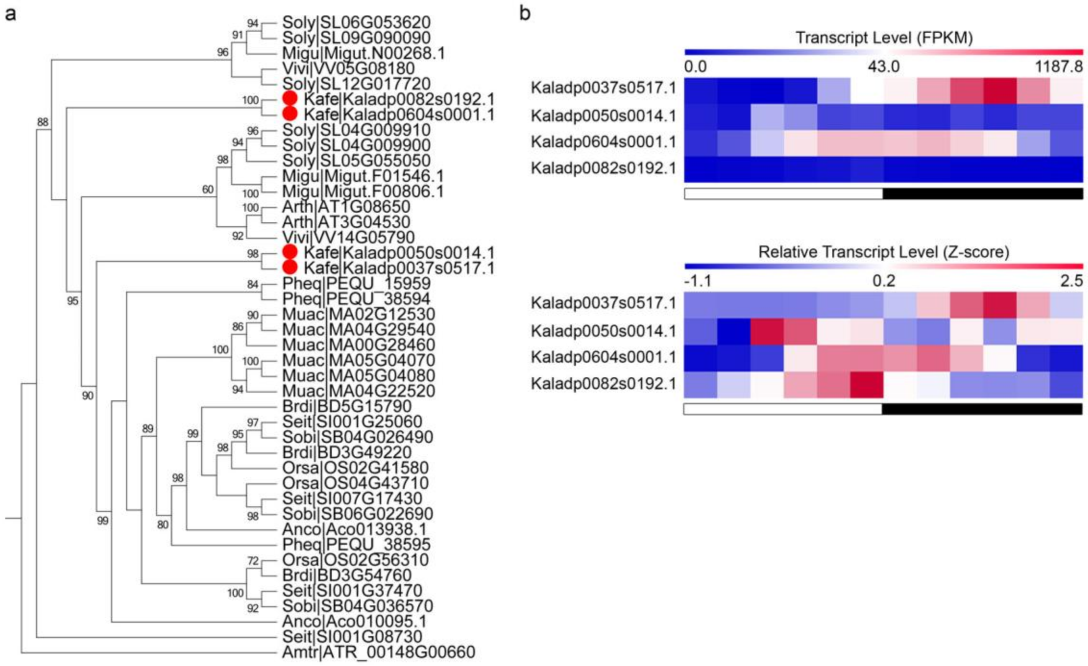

Supplementary Figure 24. Phylogeny of the phospho*enol*pyruvate carboxylase kinase (PPCK) proteins and diel transcript expression. (**a**) A maximum-likelihood phylogenetic tree constructed from protein sequences. (**b**) Diel transcript expression of the *PPCK* genes in *Kalanchoë fedtschenkoi*. The taxon names in the phylogenetic tree are listed as species abbreviation (the first four letters, see Supplementary Table 9) followed by gene/transcript name. White and black bars indicate daytime (12-hour) and nighttime (12-hour), respectively.

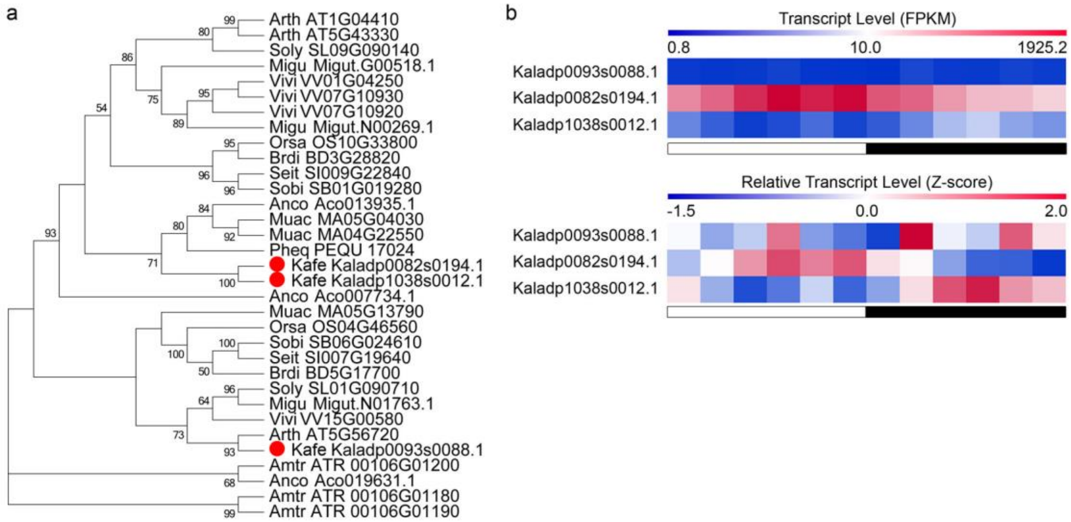

Supplementary Figure 25. Phylogeny of the NAD(P)-malate dehydrogenase (MDH) subfamily-1 proteins and diel transcript expression. (**a**) A maximum-likelihood phylogenetic tree constructed from protein sequences. (**b**) Diel transcript expression of the *MDH* subfamily-1 genes in *Kalanchoë fedtschenkoi*. The taxon names in the phylogenetic tree are listed as species abbreviation (the first four letters, see Supplementary Table 9) followed by gene/transcript name. White and black bars indicate daytime (12-hour) and nighttime (12-hour), respectively.

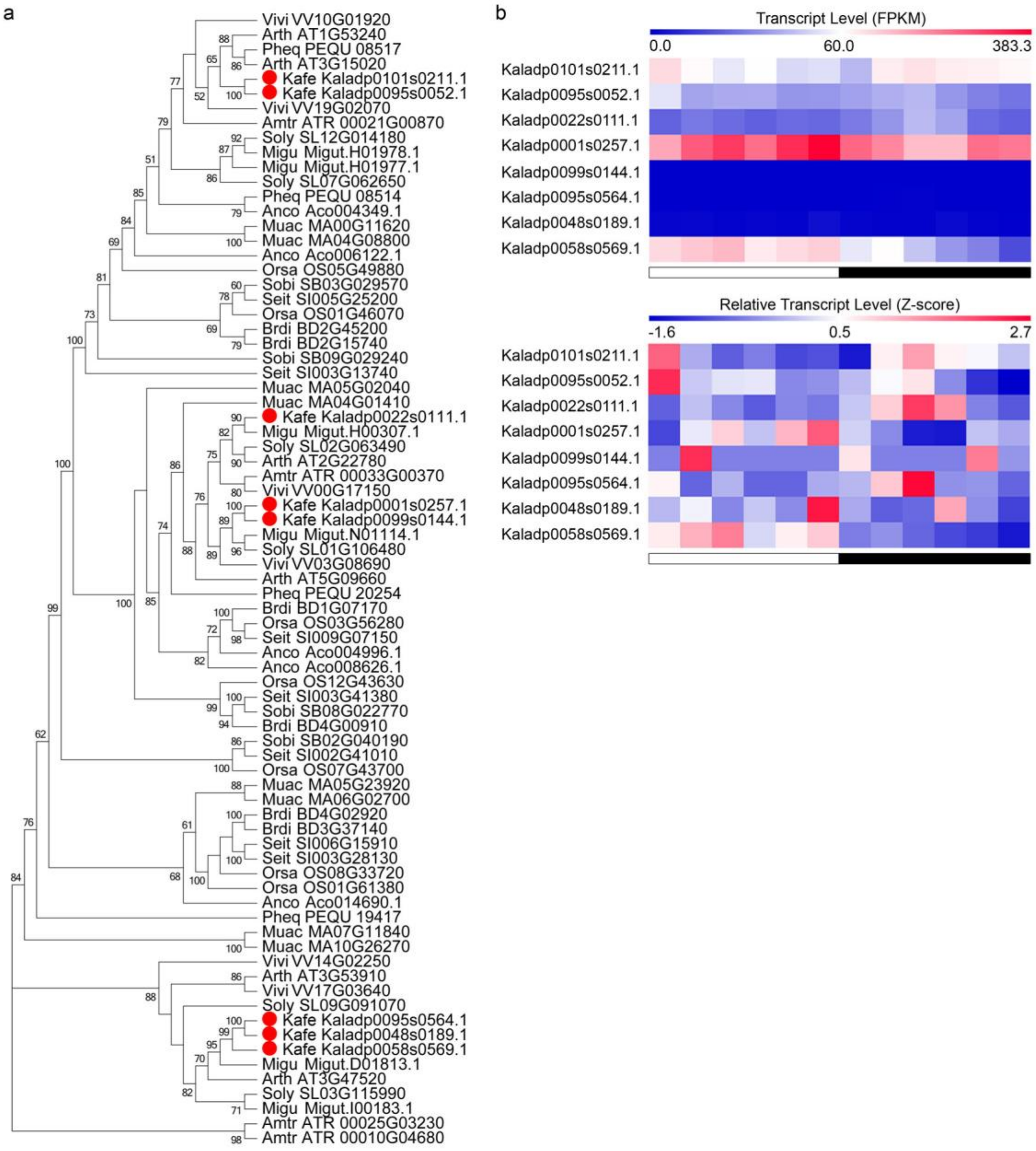

Supplementary Figure 26. Phylogeny of the NAD(P)-malate dehydrogenase (MDH) subfamily-2 proteins and diel transcript expression. (**a**) A maximum-likelihood phylogenetic tree constructed from protein sequences. (**b**) Diel transcript expression of the *MDH* subfamily-2 genes in *Kalanchoë fedtschenkoi*. The taxon names in the phylogenetic tree are listed as species abbreviation (the first four letters, see Supplementary Table 9) followed by gene/transcript name. White and black bars indicate daytime (12-hour) and nighttime (12-hour), respectively.

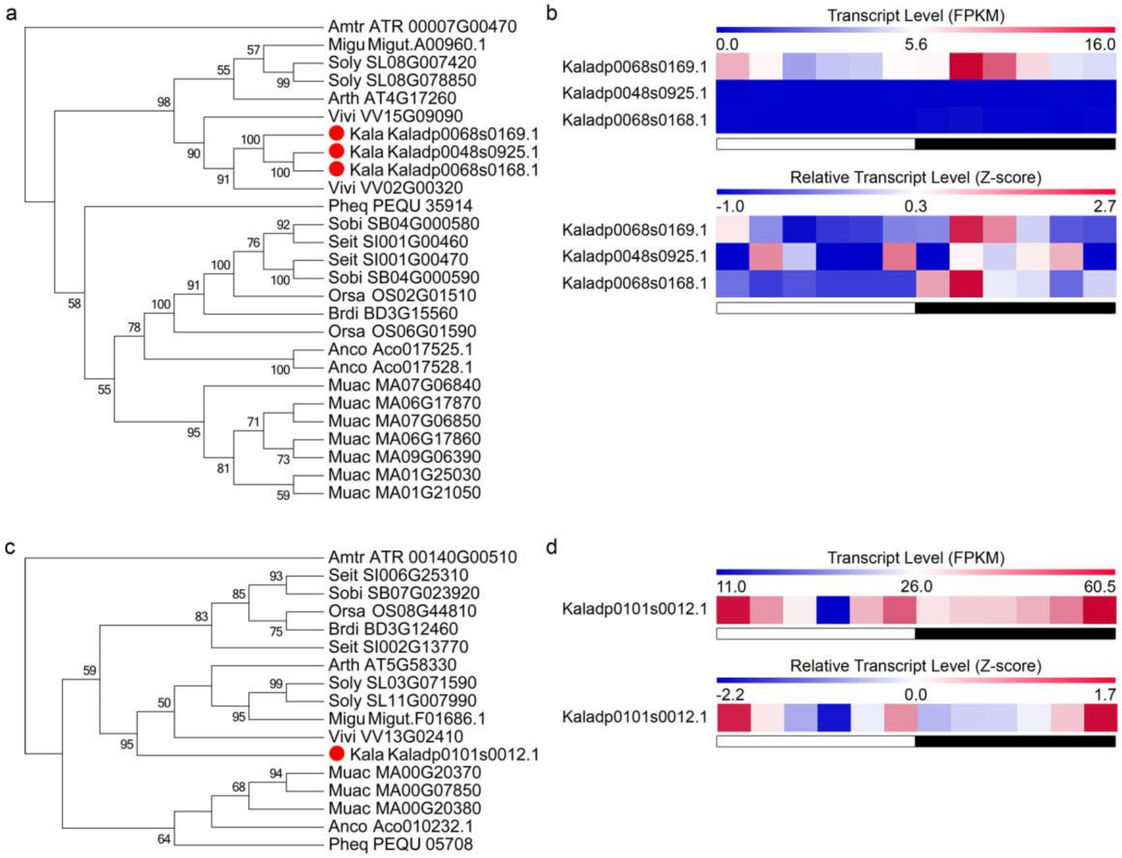

Supplementary Figure 27. Phylogeny of the NAD(P)-malate dehydrogenase (MDH) subfamily-3 and 4 proteins and diel transcript expression in *Kalanchoë fedtschenkoi*. (**a**) and (**c**) Maximum-likelihood phylogenetic trees constructed from protein sequences of MDH subfamily-3 and 4, respectively. (**b**) and (**d**) Diel transcript expression of the *MDH* subfamily-3 and 4, respectively. The taxon names in the phylogenetic tree are listed as species abbreviation (the first four letters, see Supplementary Table 9) followed by gene/transcript name. White and black bars indicate daytime (12-hour) and nighttime (12-hour), respectively.

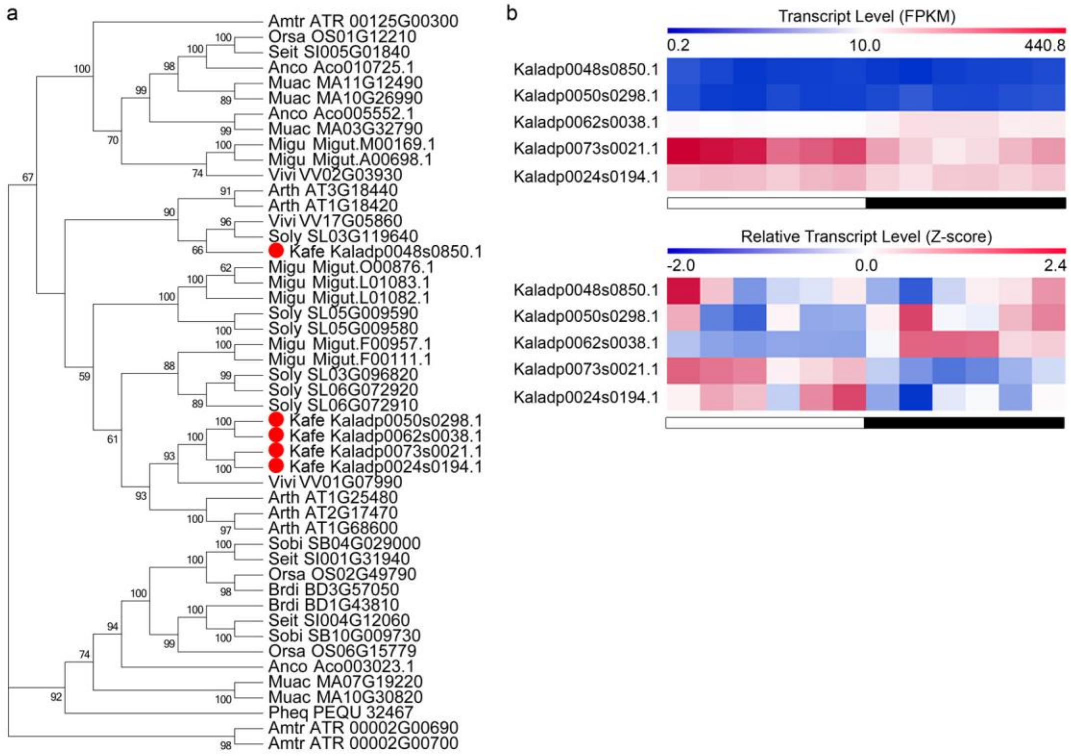

Supplementary Figure 28. Phylogeny of the aluminum-activated malate transporter (ALMT) subfamily-1 proteins and diel transcript expression. (**a**) A maximum-likelihood phylogenetic tree constructed from protein sequences. (**b**) Diel transcript expression of the *ALMT* subfamily-1 genes in *Kalanchoë fedtschenkoi*. The taxon names in the phylogenetic tree are listed as species abbreviation (the first four letters, see Supplementary Table 9) followed by gene/transcript name. White and black bars indicate daytime (12-hour) and nighttime (12-hour), respectively.

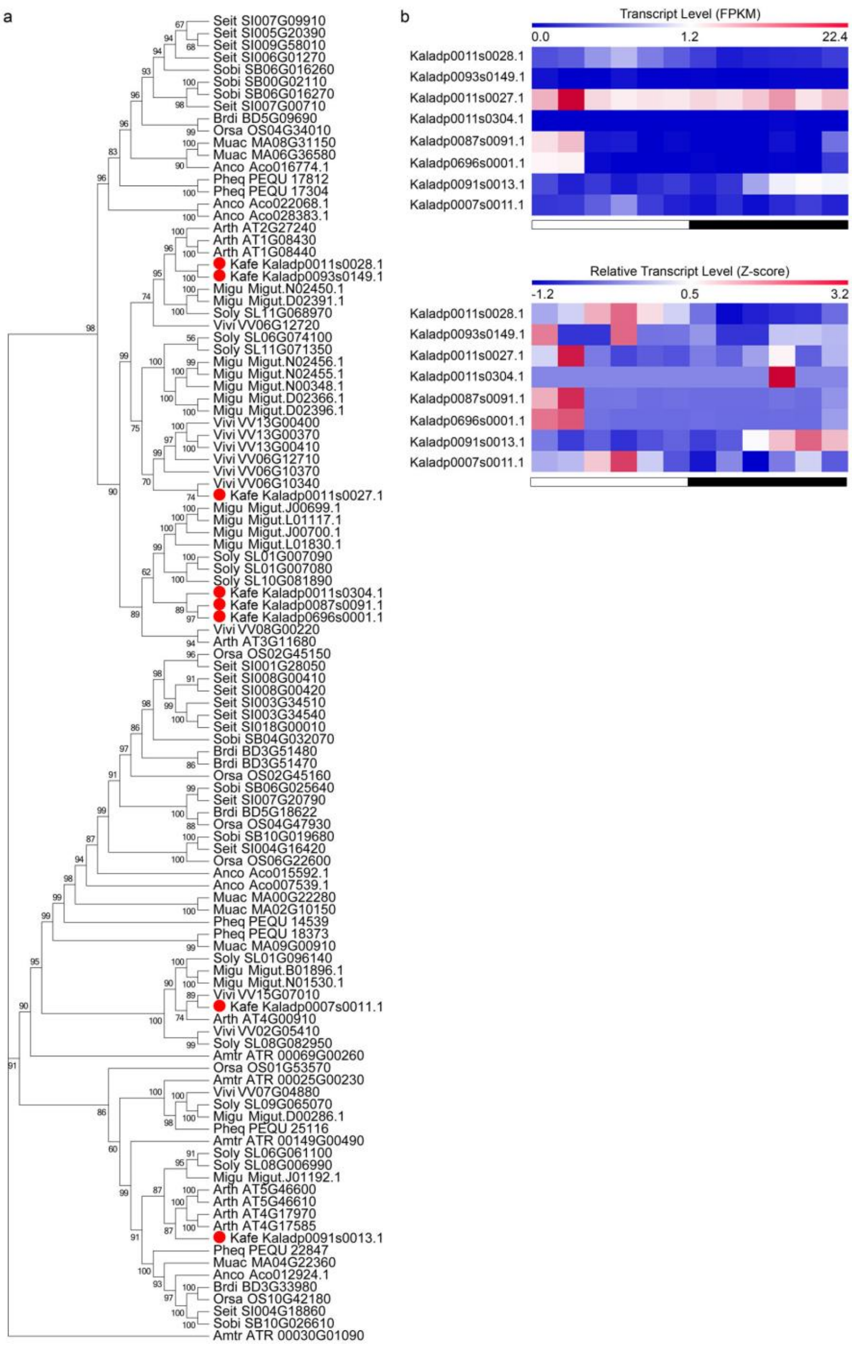

Supplementary Figure 29. Phylogeny of the aluminum-activated malate transporter (ALMT) subfamily-2 proteins and diel transcript expression. (**a**) A maximum-likelihood phylogenetic tree constructed from protein sequences. (**b**) Diel transcript expression of the *ALMT* subfamily-2 genes in *Kalanchoë fedtschenkoi*. The taxon names in the phylogenetic tree are listed as species abbreviation (the first four letters, see Supplementary Table 9) followed by gene/transcript name. White and black bars indicate daytime (12-hour) and nighttime (12-hour), respectively.

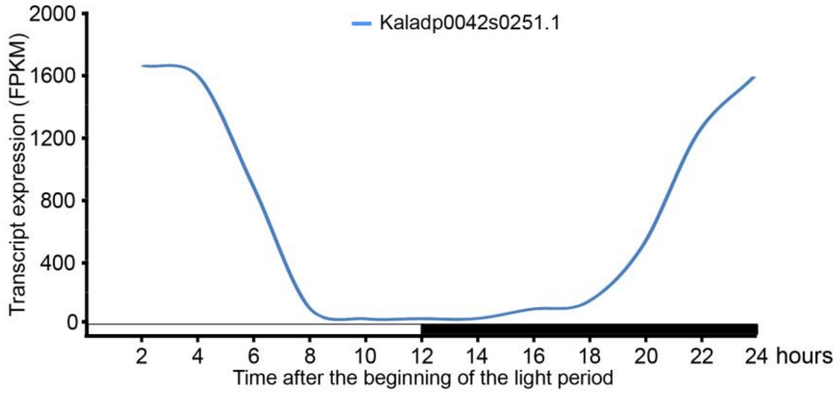

Supplementary Figure 30. Diel transcript expression of the tonoplast dicarboxylate transporter (*TDT*) gene in *Kalanchoë fedtschenkoi*. White and black bars indicate daytime (12-hour) and nighttime (12-hour), respectively.

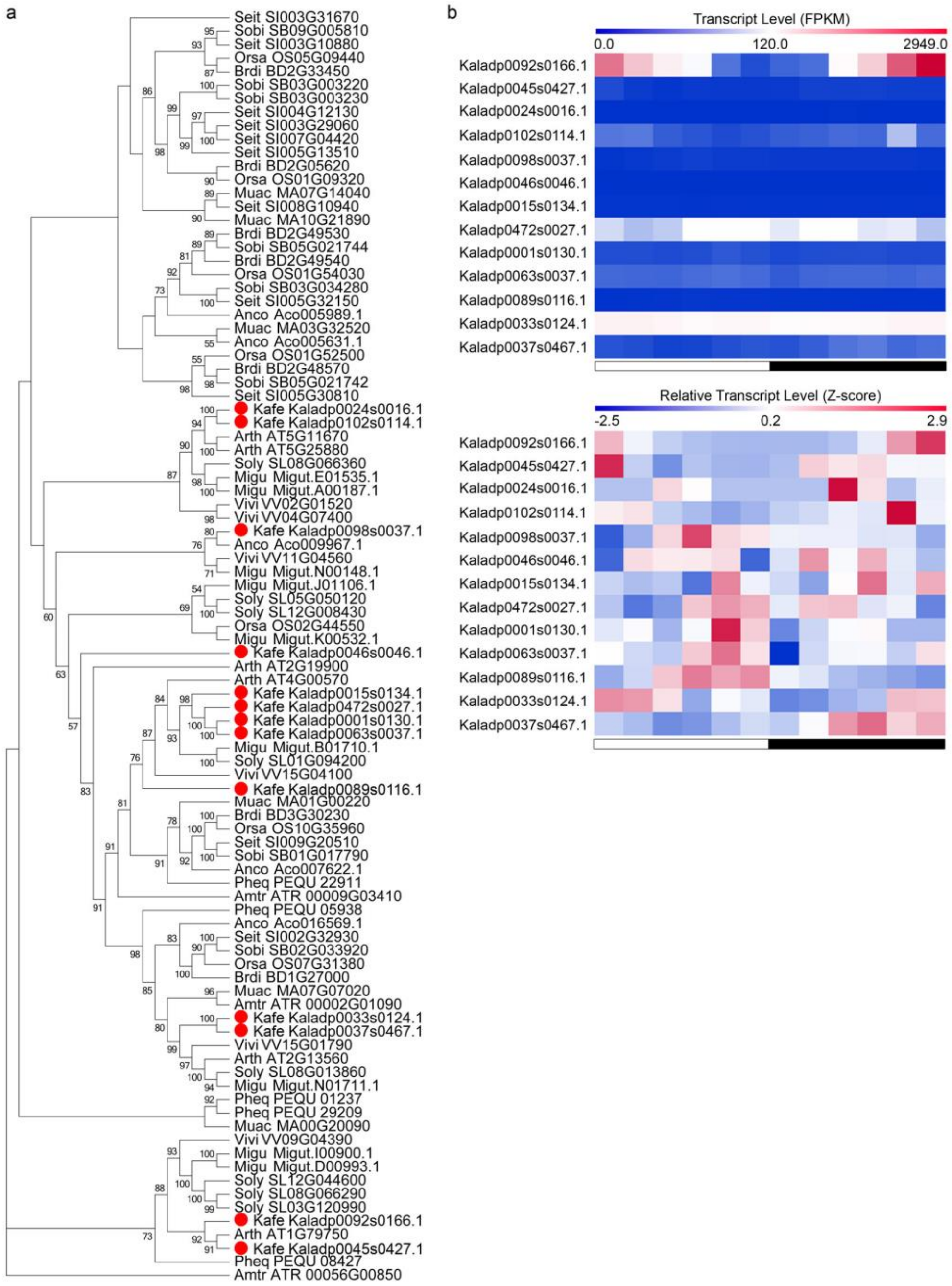

Supplementary Figure 31. Phylogeny of the NAD(P)-malic enzyme (ME) proteins and diel transcript expression. (**a**) A maximum-likelihood phylogenetic tree constructed from protein sequences. (**b**) Diel transcript expression of the *NAD(P)-ME* genes in *Kalanchoë fedtschenkoi*. The taxon names in the phylogenetic tree are listed as species abbreviation (the first four letters, see Supplementary Table 9) followed by gene/transcript name. White and black bars indicate daytime (12-hour) and nighttime (12-hour), respectively.

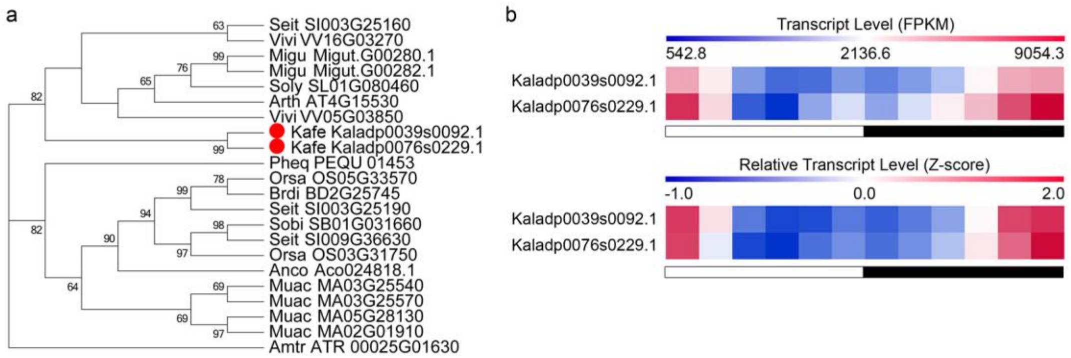

Supplementary Figure 32. Phylogeny of the pyruvate phosphate dikinase (PPDK) proteins and diel transcript expression. (**a**) A maximum-likelihood phylogenetic tree constructed from protein sequences. (**b**) Diel transcript expression of the *PPDK* genes in *Kalanchoë fedtschenkoi*. The taxon names in the phylogenetic tree are listed as species abbreviation (the first four letters, see Supplementary Table 9) followed by gene/transcript name. White and black bars indicate daytime (12-hour) and nighttime (12-hour), respectively.

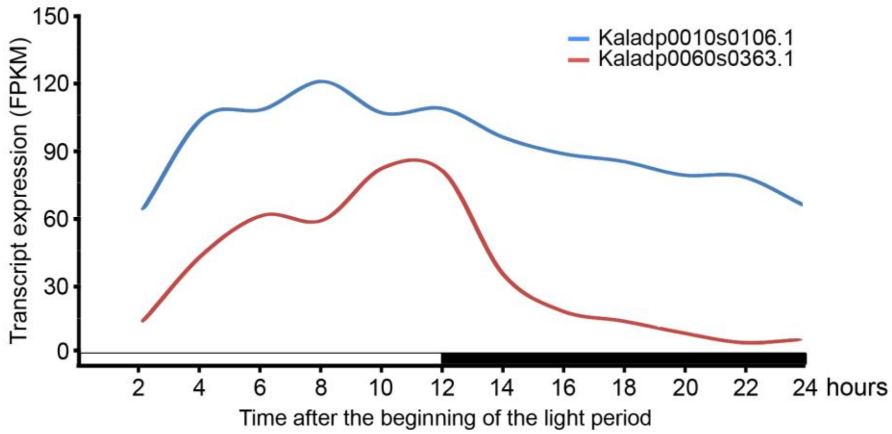

Supplementary Figure 33. Diel transcript expression of pyruvate, phosphate dikinase regulatory protein (*PPDK-RP*) genes in *Kalanchoë fedtschenkoi*. White and black bars indicate daytime (12-hour) and nighttime (12-hour), respectively.

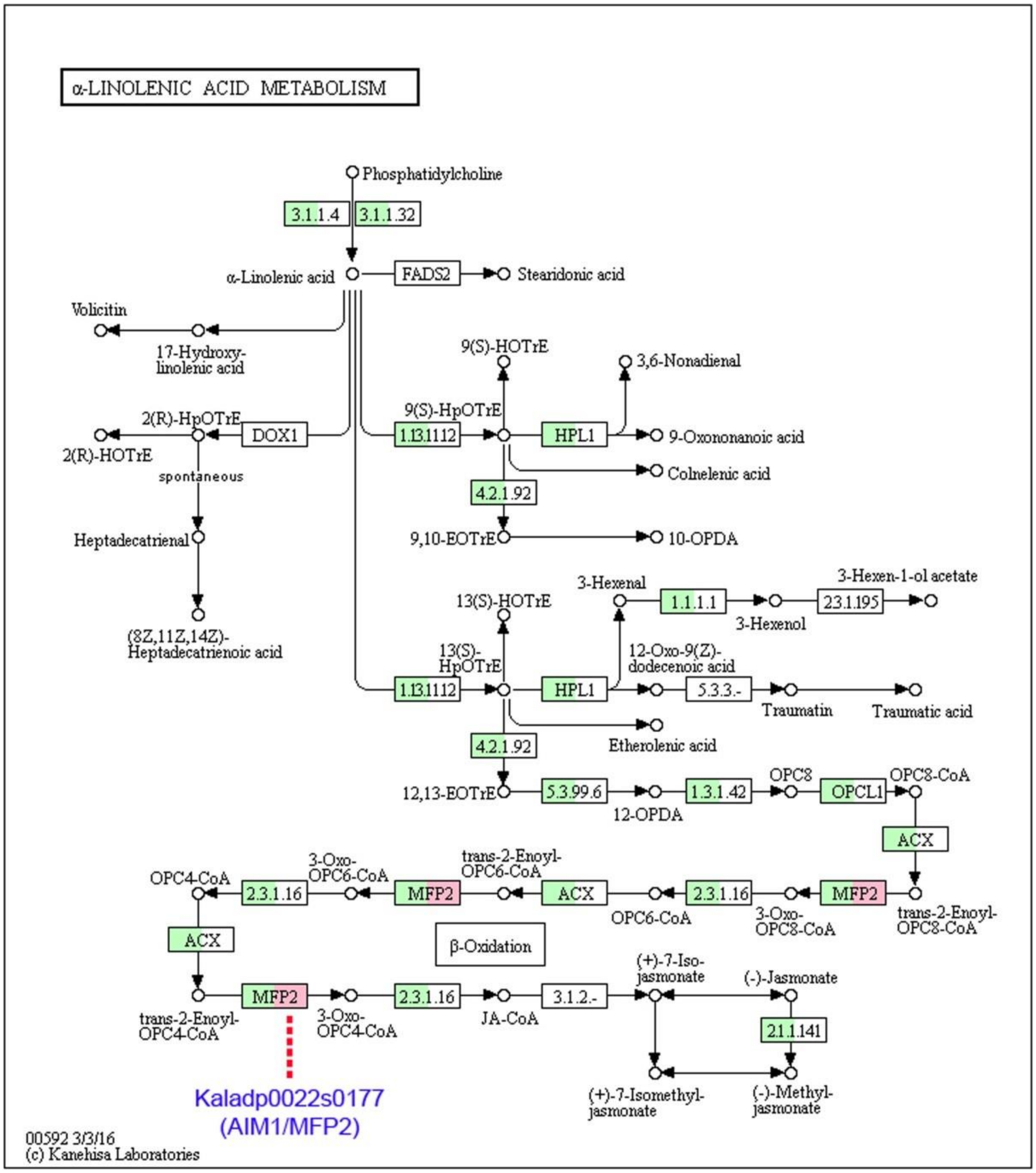

Supplementary Figure 34. An overview KEGG map of jasmonic acid biosynthesis. Green color indicates the genes identified in *Kalanchoë fedtschenkoi*. Pink color highlights the gene (Kaladp0022s0177) showing convergent evolution with *Ananas comosus* (pineapple) in diel expression pattern. AIM1: peroxisomal fatty acid beta-oxidation multifunctional protein AIM1.

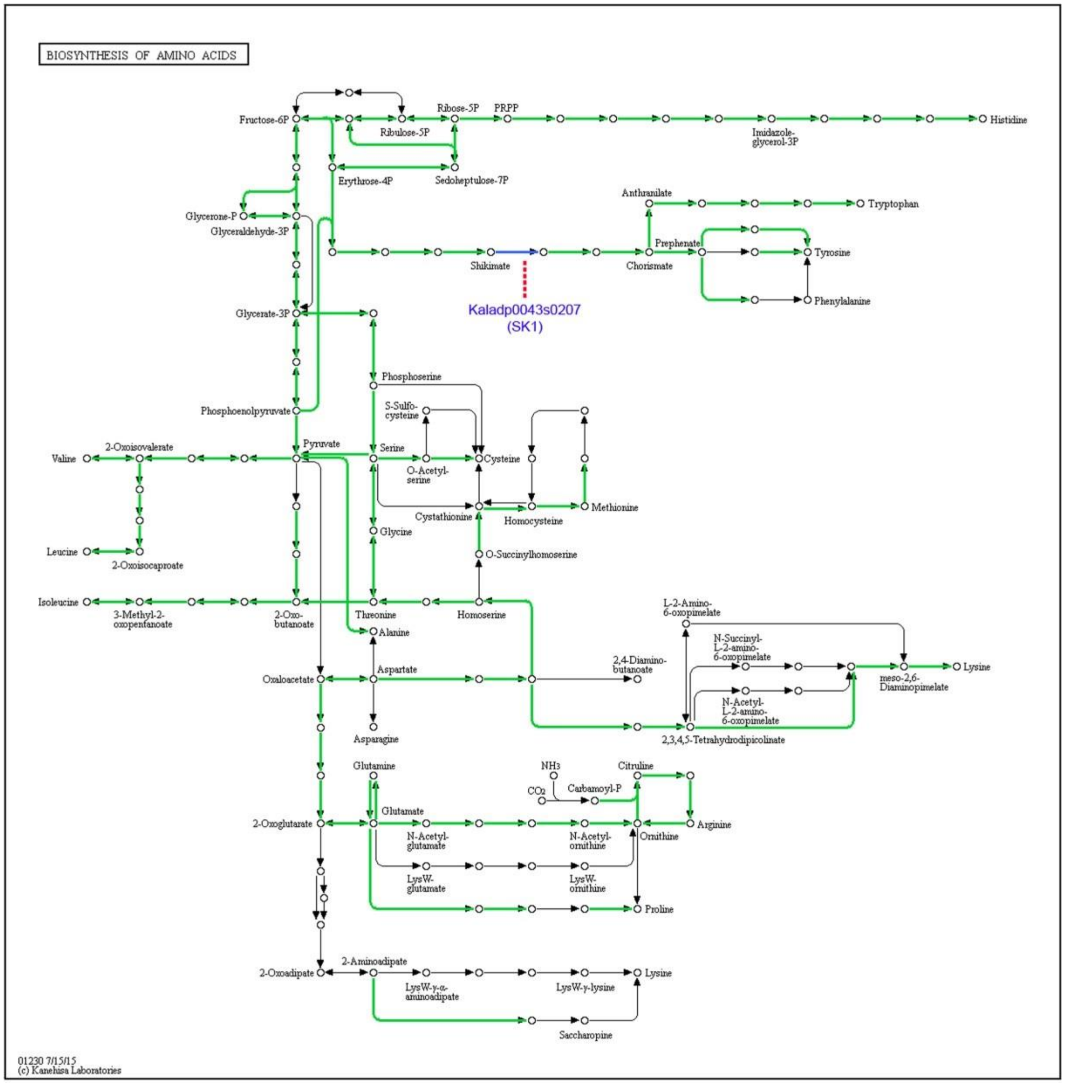

Supplementary Figure 35. An overview KEGG map of aromatic amino acid biosynthesis. Green color indicates the genes identified in *Kalanchoë fedtschenkoi*. Blue color highlights the *K. fedtschenkoi* gene (Kaladp0043s0207) showing convergent evolution with *Ananas comosus* (pineapple) in diel expression pattern. SK1: shikimate kinase 1.

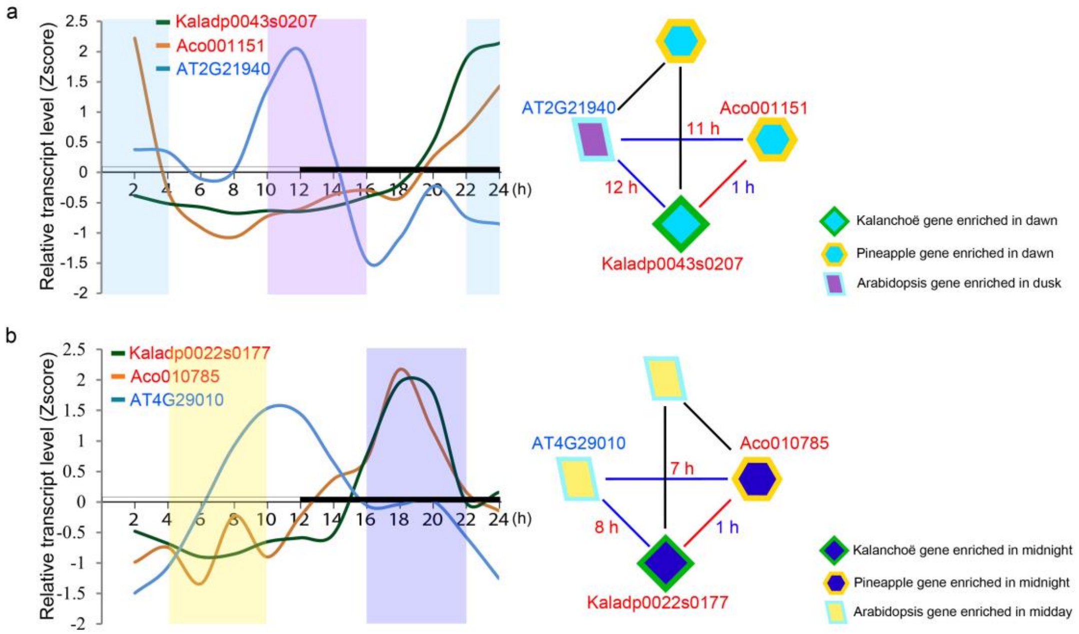

Supplementary Figure 36. Enrichment of triangle networks of genes relevant to secondary metabolism in *Kalanchoë fedtschenkoi*, pineapple and *Arabidopsis*. (**a**) The *K. fedtschenkoi* gene (Kaladp0043s0207) encodes a shikimate kinase (SS) to regulate aromatic amino acid biosynthesis. (**b**) The *K. fedtschenkoi* gene (Kaladp0022s0177) encodes AIM1/MFP2 that is involved in the biosynthesis of jasmonic acid. White and black bars indicate daytime (12-hour) and nighttime (12-hour), respectively. X-axis represents the time after the beginning of the light period.

	Dawn	Dusk
Gene i	d_{i1}	d_{i2}
¬Gene i	$\sum_{j\neq i} d_{j1}$	$\sum_{j\neq i} d_{j2}$

	Dusk	Dawn
Gene i	d_{i2}	d_{i1}
¬Gene i	$\sum_{j\neq i} d_{j2}$	$\sum_{j\neq i} d_{j1}$

	Midday	Midnight
Gene i	m_{i1}	m_{i2}
¬Gene i	$\sum_{j\neq i} m_{j1}$	$\sum_{j\neq i} m_{j2}$

	Midnight	Midday
Gene i	m_{i2}	m_{i1}
¬Gene i	$\sum_{j\neq i} m_{j2}$	$\sum_{j\neq i} m_{j1}$

Supplementary Figure 37. Contingency tables for Fisher Exact Test to determine the enrichment of gene expression between contrasting time windows. Midday: 4, 6, and 8 h from the starting of the light period; midnight: 16, 18, and 20 h from the starting of the light period; dawn: 22, 24, and 2 h from the starting of the light period; and dusk: 10, 12, and 14 h from the starting of the light period.

Supplementary Tables

Supplementary Table 1. Estimated DNA content in *Kalanchoë fedtschenkoi*.

Sample	DNA ratio with internal standard*	DNA pg/2C	DNA (Mbp/C)
KLM-1	0.342	0.52	253
KLM-2	0.351	0.53	259
KLM-3	0.345	0.52	255
**Average**	**0.346**	**0.52**	**256**

* Internal standard: *Vinca minor* (DNA= 1.51 pg/2C = 1477 Mbp/2C).

Supplementary Table 2. Sequencing reads generated for *Kalanchoë fedtschenkoi* using by Illumina MiSeq platform.

Library type	Fragment length	Reads	Coverage, X
Paired-end	540 bp	2x 300 bp	70
Mate-pair	3 Kb	2x 300 bp	14
Mate-pair	6 Kb	2x 300 bp	12
Mate-pair	11 Kb	2x 300 bp	11

Supplementary Table 3. Statistics of genome assembly (Scaffolds) for *Kalanchoë fedtschenkoi*.

Total number of sequences	1,324
Total bases	256,351,415
Min sequence length, bp	1,002
Max sequence length, bp	10,491,024
Average sequence length, bp	193,619
Median sequence length, bp	8,120
N25 length, bp	5,483,823
N50 length, bp	2,451,343
N75 length, bp	1,051,141
N90 length, bp	258,600
N95 length, bp	77,720
As	29.83%
Ts	29.86%
Gs	17.23%
Cs	17.23%
(A + T)s	59.69%
(G + C)s	34.47%
Ns	5.85%

Supplementary Table 4. Statistics of genome annotation for *Kalanchoë fedtschenkoi*.

Primary transcripts (loci)	30,964
Alternative transcripts	14,226
Total transcripts	45,190
**For primary transcripts:**	
Average number of exons	5.1
Median exon length	161
Median intron length	126
**Gene model support:**	
Any EST support	25,740
EST support over 100% of their lengths	22,751
EST support over 95% of their lengths	23,476
EST support over 90% of their lengths	23,775
EST support over 75% of their lengths	24,330
EST support over 50% of their lengths	24,870
Peptide homology coverage of 100%	1,729
Peptide homology coverage of over 95%	16,118
Peptide homology coverage of over 90%	19,939
Peptide homology coverage of over 75%	24,305
Peptide homology coverage of over 50%	27,502
Pfam annotation	21,794
Panther annotation	27,197
KOG annotation	12,093
KEGG Orthology annotation	4,656
E.C. number annotation	2,452

Supplementary Table 5. List of genes involved in CAM carboxylation process in *Kalanchoë fedtschenkoi.*

Tribe_id	Name	Gene locus	Definition	Gene expression (FPKM)[a]	Gene Co-expression module[b]	Cluster[c]	Profile-variation[d]
I50_F000796	β-CA	Kaladp0018s0287.1	Beta carbonic anhydrase	219.37	MEgreen	3	non-flat
I50_F000796	β-CA	Kaladp0018s0289.1	Beta carbonic anhydrase	352.68	MEblack	3	non-flat
I50_F000796	β-CA	Kaladp0024s0122.1	Beta carbonic anhydrase	18.35	MEpink	8	non-flat
I50_F000796	β-CA	Kaladp0034s0051.1	Beta carbonic anhydrase	277.08	MEsalmon	N/A	non-flat
I50_F000796	β-CA	Kaladp0081s0140.1	Beta carbonic anhydrase	1.14	MEmagenta	10	non-flat
I50_F000796	β-CA	Kaladp0081s0143.1	Beta carbonic anhydrase	2.39	MElightcyan	N/A	flat
I50_F000796	β-CA	Kaladp0538s0011.1	Beta carbonic anhydrase	66.8	MEtan	1	flat
I50_F000022	PPCK1	Kaladp0037s0517.1	Phosphoenolpyruvate carboxylase kinase	1187.84	MEblack	3	non-flat
I50_F000022	PPCK	Kaladp0050s0014.1	Phosphoenolpyruvate carboxylase kinase	37.58	MElightyellow	N/A	flat
I50_F000022	PPCK	Kaladp0082s0192.1	Phosphoenolpyruvate carboxylase kinase	6.62	MEpurple	7	non-flat
I50_F000022	PPCK2	Kaladp0604s0001.1	Phosphoenolpyruvate carboxylase kinase	376.3	MElightgreen	7	non-flat
I50_F000807	PEPC	Kaladp0011s0355.1	Phosphoenolpyruvate carboxylase	10.79	MEtan	6	non-flat
I50_F000807	PEPC	Kaladp0011s1355.1	Phosphoenolpyruvate carboxylase	3.29	MEgrey	N/A	flat
I50_F000807	PEPC2	Kaladp0048s0578.1	Phosphoenolpyruvate carboxylase 2	280.38	MEblack	3	non-flat
I50_F000807	PEPC	Kaladp0062s0055.1	Phosphoenolpyruvate carboxylase	18.51	MEgrey	2	non-flat
I50_F000807	PEPC1	Kaladp0095s0055.1	Phosphoenolpyruvate carboxylase 1	8338.11	MEpink	7	non-flat
I50_F000614	MDH	Kaladp0001s0257.1	Malate dehydrogenase	383.29	MEpink	11	non-flat
I50_F000614	MDH	Kaladp0022s0111.1	Malate dehydrogenase	43.82	MEblack	3	non-flat
I50_F000614	MDH	Kaladp0048s0189.1	Malate dehydrogenase	4.27	MEgrey	N/A	flat
I50_F000614	MDH	Kaladp0058s0569.1	Malate dehydrogenase	150.19	MEblue	2	non-flat
I50_F000614	MDH	Kaladp0095s0052.1	Malate dehydrogenase	107.8	MEbrown	1	non-flat
I50_F000614	MDH	Kaladp0095s0564.1	Malate dehydrogenase	1.17	MEgreenyellow	N/A	flat
I50_F000614	MDH	Kaladp0101s0211.1	Malate dehydrogenase	236.21	MEtan	1	non-flat
I50_F001882	MDH	Kaladp0082s0194.1	Malate dehydrogenase	1925.18	MEblue	10	non-flat
I50_F001882	MDH	Kaladp0093s0088.1	Malate dehydrogenase	3.08	MEturquoise	N/A	flat
I50_F001882	MDH	Kaladp1038s0012.1	Malate dehydrogenase	19.58	MEturquoise	N/A	flat
I50_F002831	MDH	Kaladp0068s0169.1	Malate dehydrogenase	226.83	MEturquoise	5	non-flat
I50_F004944	MDH	Kaladp0101s0012.1	Malate dehydrogenase	60.4	MEsalmon	N/A	flat
I50_F001112	ALMT	Kaladp0024s0194.1	Tonoplast aluminum-activated malate transporter	147.01	MEpink	N/A	flat
I50_F001112	ALMT	Kaladp0048s0850.1	Tonoplast aluminum-activated malate transporter	8.52	MEturquoise	N/A	flat
I50_F001112	ALMT	Kaladp0050s0298.1	Tonoplast aluminum-activated malate transporter	2.49	MEtan	N/A	flat
I50_F001112	ALMT6	Kaladp0062s0038.1	Tonoplast aluminum-activated malate transporter	66.11	MEblack	3	non-flat
I50_F001112	ALMT6	Kaladp0073s0021.1	Tonoplast aluminum-activated malate transporter 6	440.84	MEpink	2	non-flat
I50_F000383	ALMT	Kaladp0007s0011.1	Tonoplast aluminum-activated malate transporter	1.11	MEbrown	10	non-flat
I50_F000383	ALMT	Kaladp0011s0027.1	Tonoplast aluminum-activated malate transporter	123.96	MEturquoise	2	non-flat
I50_F000383	ALMT	Kaladp0011s0028.1	Tonoplast aluminum-activated malate transporter	2.97	MEgrey	N/A	flat
I50_F000383	ALMT	Kaladp0087s0091.1	Tonoplast aluminum-activated malate transporter	6.68	MEdarkgreen	2	non-flat
I50_F000383	ALMT	Kaladp0091s0013.1	Tonoplast aluminum-activated malate transporter	1.54	MEsalmon	4	non-flat
I50_F000383	ALMT	Kaladp0093s0149.1	Tonoplast aluminum-activated malate transporter	17.78	MEturquoise	N/A	flat

[a]The maximum expression level in the mature leaf during 24-h period, as revealed by RNA-seq analysis.
[b]The gene co-expression module listed in Supplementary Fig. 9.
[c]Gene clusters constructed, using maSigPro [5, 6], from the transcripts that showed significantly (ANOVA of glm models where H_0 = a flat line, P<0.05) "non-flat" diel expression patterns.
[d]The significance of variation of transcript abundance over a 24-h period was determined by a polynomial regression. "Non-flat" indicates that the transcript abundance varied significantly (ANOVA of glm models where H_0 = a flat line, P<0.05) across time points. "Flat" indicates that the transcript abundance did not vary significantly (ANOVA of glm models where H_0 = a flat line, P<0.05) across time points.

Supplementary Table 6. List of genes involve in CAM decarboxylation process in *Kalanchoë fedtschenkoi*.

Tribe_id	Name	Gene locus	Definition	Gene expression (FPKM)[a]	Gene Co-expression module[b]	Cluster[c]	Profile-variation[d]
I50_F004828	TDT	Kaladp0042s0251.1	Tonoplast dicarboxylate transporter	1617.63	MEsalmon	1	non-flat
I50_F003433	PPDK	Kaladp0039s0092.1	Pyruvate, orthophosphate dikinase	4744.54	MEpink	1	non-flat
I50_F003433	PPDK	Kaladp0076s0229.1	Pyruvate, orthophosphate dikinase	9054.27	MEsalmon	1	non-flat
I50_F005114	PPDK-RP	Kaladp0010s0106.1	PPDK regulatory protein	121.13	MEblue	10	non-flat
I50_F005114	PPDK-RP	Kaladp0060s0363.1	PPDK regulatory protein	81.78	MEblue	11	non-flat
I50_F001811	PEPCK	Kaladp0023s0088.1	Phosphoenolpyruvate carboxykinase	17.45	MEgrey	N/A	flat
I50_F001811	PEPCK	Kaladp0040s0194.1	Phosphoenolpyruvate carboxykinase	78.13	MEturquoise	4	non-flat
I50_F001811	PEPCK	Kaladp1116s0004.1	Phosphoenolpyruvate carboxykinase	9.04	MEgrey	3	non-flat
I50_F000472	NAD-ME	Kaladp0001s0130.1	NAD-dependent malic enzyme	21.76	MEcyan	N/A	flat
I50_F000472	NAD-ME	Kaladp0015s0134.1	NAD-dependent malic enzyme	20.74	MEgrey	N/A	flat
I50_F000472	NADP-ME	Kaladp0024s0016.1	NADP-malic enzyme	1.01	MEgrey	N/A	flat
I50_F000472	NAD-ME	Kaladp0033s0124.1	NAD-dependent malic enzyme	269.31	MEpink	1	non-flat
I50_F000472	NAD-ME	Kaladp0037s0467.1	NAD-dependent malic enzyme	83.61	MEgreen	3	non-flat
I50_F000472	NADP-ME	Kaladp0045s0427.1	NADP-malic enzyme	204.39	MEturquoise	1	non-flat
I50_F000472	NAD-ME	Kaladp0063s0037.1	NAD-dependent malic enzyme	36.49	MEcyan	1	non-flat
I50_F000472	NADP-ME	Kaladp0092s0166.1	NADP-malic enzyme	2948.96	MEsalmon	4	non-flat
I50_F000472	NAD-ME	Kaladp0472s0027.1	NAD-dependent malic enzyme	129.8	MEmidnightblue	N/A	flat

[a]The maximum expression level in the mature leaf during 24-h period, as revealed by RNA-seq analysis.
[b]The gene co-expression module listed in Supplementary Fig. 9.
[c]Gene clusters constructed, using maSigPro [5, 6], from the transcripts that showed significantly (ANOVA of glm models where H_0 = a flat line, $P<0.05$) "non-flat" diel expression patterns.
[d]The significance of variation of transcript abundance over a 24-h period was determined by a polynomial regression. "Non-flat" indicates that the transcript abundance varied significantly (ANOVA of glm models where H_0 = a flat line, $P<0.05$) across time points. "Flat" indicates that the transcript abundance did not vary significantly (ANOVA of glm models where H_0 = a flat line, $P<0.05$) across time points.

Supplementary Table 7: Number of genes shared between *Kalanchoë fedtschenkoi* co-expression modules (Supplementary Fig. 9) and genes clusters (Supplementary Fig. 11). The gene clusters were constructed, using maSigPro [5, 6], from the transcripts that showed significantly (ANOVA of glm models where H_0 = a flat line, *p*-value<0.05) "non-flat" diel expression patterns. The numbers in parentheses are number of genes in each co-expression module or gene cluster.

Module	Cluster 1 (1208)	Cluster 2 (1274)	Cluster 3 (1751)	Cluster 4 (2146)	Cluster 5 (1381)	Cluster 6 (1061)	Cluster 7 (1797)	Cluster 8 (927)	Cluster 9 (1107)	Cluster 10 (1717)	Cluster 11 (2077)
MEblack (782)	1	0	298	43	80	8	2	0	181	1	1
MEblue (1911)	0	75	0	4	3	2	285	100	12	297	717
MEbrown(1593)	6	254	9	1	70	1	136	20	17	380	247
MEcyan (1048)	17	2	100	348	4	125	11	30	8	2	3
MEdarkgreen (437)	79	100	6	15	0	5	1	81	1	5	12
MEdarkgrey (615)	24	0	63	341	1	21	0	2	1	3	0
MEdarkred (410)	2	20	1	0	20	1	113	5	19	48	57
MEdarkturquoise (369)	176	29	5	34	0	3	0	16	0	2	0
MEgreen (1031)	12	2	555	105	337	71	7	3	171	9	0
MEgreenyellow (733)	7	1	200	6	249	18	4	0	38	8	0
MEgrey (2277)	85	108	108	110	95	43	186	37	79	74	70
MEgrey60 (506)	0	42	3	0	59	9	134	1	18	35	34
MElightcyan (731)	6	90	0	2	0	13	190	31	3	2	89
MElightgreen (504)	1	1	12	1	97	12	81	0	149	13	2
MElightyellow (641)	12	56	12	5	22	1	6	42	6	171	18
MEmagenta (638)	0	1	25	3	295	16	35	1	121	8	2
MEmidnightblue (514)	2	4	8	1	77	1	73	2	76	124	9
MEpink (1353)	64	93	22	111	12	18	59	245	16	121	155
MEpurple (1509)	36	114	22	41	24	22	153	94	52	48	119
MEred (1277)	43	7	25	72	7	520	15	5	7	1	6
MEroyalblue (408)	3	1	72	86	12	2	3	26	8	36	4
MEsalmon (731)	128	0	10	403	0	7	1	15	0	0	1
MEtan (688)	196	24	39	101	6	12	3	15	3	7	2
MEturquoise (3052)	259	203	262	162	172	120	75	66	100	81	45
MEyellow (1144)	1	35	0	2	1	1	210	27	8	192	425

Supplementary Table 8: Time-shifts in diel transcript expression profiles between orthologs in *Kalanchoë fedtschenkoi* (Kafe), *Ananas comosus* (Anco) and *Arabidopsis thaliana* (Arth). Only the gene pairs containing *K. fedtschenkoi* and *A. comosus* genes that showed convergent changes in diel expression pattern were shown here. "Xcorr" is the cross correlation. "Time" is time-shift in hours.

Name	*Kalanchoë*	Pineapple	*Arabidopsis*	Kafe vs. Arth		Anco vs. Arth		Kafe vs. Anco	
				Time	Xcorr	Time	Xcorr	Time	Xcorr
PHOT2	Kaladp0033s0113.1	Aco014242.1	AT5G58140	11	0.58	9	0.63	1	0.87
PPR	Kaladp0058s0382.1	Aco019522.1	AT1G02060	12	0.67	11	0.72	0	0.91
DAG	Kaladp0130s0003.1	Aco002485.1	AT3G06790	11	0.82	11	0.90	0	0.92
TPX2	Kaladp0062s0086.1	Aco009655.1	AT3G23090	9	0.88	9	0.85	1	0.90
MRI1	Kaladp0053s0416.1	Aco007956.1	AT2G05830	11	0.87	11	0.72	0	0.78
NUTF2	Kaladp0040s0332.1	Aco015511.1	AT2G46100	7	0.84	6	0.79	1	0.88
bcrC	Kaladp0840s0040.1	Aco007440.1	AT2G34670	8	0.69	8	0.70	0	0.91
	Kaladp0045s0260.1	Aco004226.1	AT2G38820	10	0.90	11	0.94	1	0.85
PLT1	Kaladp0040s0745.1	Aco000080.1	AT2G16120	7	0.77	8	0.50	1	0.84
EXS	Kaladp0008s0539.1	Aco017367.1	AT1G55610	9	0.79	9	0.73	1	0.88
HSP70	Kaladp0060s0296.1	Aco031458.1	AT5G02500	10	0.64	9	0.71	1	0.80
CSLC12	Kaladp0037s0421.1	Aco011603.1	AT4G07960	10	0.85	9	0.89	1	0.90
PLC2	Kaladp0059s0034.1	Aco001556.1	AT3G08510	9	0.93	8	0.81	0	0.85
Lycopene cyclase protein	Kaladp0056s0132.1	Aco024665.1	AT3G10230	9	0.83	10	0.80	0	0.85
CDKI7	Kaladp0028s0063.1	Aco011817.1	AT1G49620	10	0.83	8	0.68	0	0.86
NPF	Kaladp0033s0087.1	Aco013262.1	AT2G26690	11	0.93	8	0.74	2	0.83
TIM23	Kaladp1244s0001.1	Aco007546.1	AT1G72750	6	0.92	7	0.86	1	0.83
IAA29	Kaladp0048s0752.1	Aco030721.1	AT4G32280	7	0.86	7	0.69	0	0.87
PRN12	Kaladp0026s0118.1	Aco015046.1	AT5G64760	9	0.84	12	0.66	1	0.68
PDK	Kaladp0068s0282.1	Aco017179.1	AT3G06483	10	0.91	11	0.88	1	0.76
OMP	Kaladp0040s0029.1	Aco018518.1	AT3G52420	9	0.76	12	0.86	2	0.82
TMEM245	Kaladp0048s0390.1	Aco003087.1	AT5G55960	12	0.81	12	0.73	1	0.81
TMEM184C	Kaladp1262s0005.1	Aco014396.1	AT1G11200	9	0.64	7	0.76	0	0.66
ACLB2	Kaladp0045s0074.1	Aco001770.1	AT3G06650	8	0.77	6	0.75	1	0.85
NPH3 family	Kaladp0040s0264.1	Aco016939.1	AT5G67440	10	0.95	9	0.77	0	0.78
SS	Kaladp0043s0207.1	Aco001151.1	AT2G21940	12	0.83	11	0.87	1	0.90
Ash2l	Kaladp0045s0419.1	Aco000634.1	AT1G51450	11	0.78	11	0.95	1	0.88
WRC	Kaladp0011s0273.1	Aco020634.1	AT2G42040	8	0.58	8	0.74	1	0.90
SNRPC1	Kaladp0349s0001.1	Aco016127.1	AT4G03120	8	0.87	11	0.88	2	0.89
COL2	Kaladp0039s0496.1	Aco002998.1	AT1G68520	11	0.73	11	0.94	1	0.85
GK	Kaladp0037s0359.1	Aco011530.1	AT1G80460	9	0.81	9	0.87	0	0.92
FH	Kaladp0040s0015.1	Aco002502.1	AT5G50950	8	0.93	9	0.87	1	0.87
PIP1-2	Kaladp0059s0048.1	Aco010251.1	AT4G23400	9	0.80	8	0.74	0	0.85
AIM1	Kaladp0022s0177.1	Aco010785.1	AT4G29010	8	0.86	7	0.82	1	0.92
HAD	Kaladp0071s0244.1	Aco001521.1	AT3G45740	11	0.75	12	0.76	0	0.82
SURE	Kaladp0427s0023.1	Aco023553.1	AT4G14930	7	0.52	8	0.74	0	0.86
EDR2L	Kaladp0098s0203.1	Aco027425.1	AT5G10750	9	0.87	12	0.74	2	0.83
CYB2	Kaladp0035s0007.1	Aco006777.1	AT5G38630	10	0.55	12	0.66	1	0.93
SEC14	Kaladp0067s0305.1	Aco003265.1	AT1G72160	11	0.73	11	0.64	1	0.88
Gdap2	Kaladp0098s0113.1	Aco002356.1	AT3G10210	9	0.56	9	0.62	1	0.84
FAH	Kaladp0060s0413.1	Aco015620.1	AT1G12050	9	0.60	10	0.47	0	0.82
	Kaladp0081s0088.1	Aco014627.1	AT1G04650	10	0.52	9	0.79	0	0.71
UFC1	Kaladp0095s0421.1	Aco014085.1	AT1G27530	9	0.89	9	0.85	1	0.77
ABCG15	Kaladp0045s0418.1	Aco007271.1	AT1G17840	11	0.80	10	0.60	1	0.81
	Kaladp0034s0049.1	Aco005409.1	AT1G23760	8	0.95	7	0.88	1	0.90
SAG	Kaladp0808s0026.1	Aco022311.1	AT2G44670	11	0.89	10	0.85	1	0.97
PPCK1	Kaladp0037s0517.1	Aco013938.1	AT1G08650	11	0.84	12	0.94	2	0.81
Starch synthase	Kaladp0055s0317.1	Aco010848.1	AT5G24300	9	0.76	7	0.73	3	0.85
SNRPC	Kaladp0018s0298.1	Aco007284.1	AT1G09070	10	0.88	10	0.79	0	0.89
Leucine rich repeat	Kaladp1251s0003.1	Aco008844.1	AT3G47090	7	0.90	7	0.74	2	0.80
CHR5	Kaladp0011s0810.1	Aco001980.1	AT2G13370	6	0.78	6	0.53	0	0.86
Leucine rich repeat	Kaladp0054s0031.1	Aco008313.1	AT2G07040	8	0.78	9	0.51	0	0.72
CINV1	Kaladp0550s0020.1	Aco007782.1	AT5G22510	11	0.83	11	0.88	1	0.79
TPS	Kaladp0011s0363.1	Aco012107.1	AT1G06410	6	0.70	6	0.56	0	0.84

Supplementary Table 9. The representative species used for identification of convergent change in protein sequences.

Photosynthesis	Species name	Abbreviation
C_3	*Amborella trichopoda*	Amtr
C_3	*Arabidopsis thaliana*	Arth
C_3	*Brachypodium distachyon*	Brdi
C_3	*Mimulus guttatus*	Migu
C_3	*Musa acuminata*	Muac
C_3	*Oryza sativa*	Orsa
C_3	*Solanum lycopersicum*	Soly
C_3	*Vitis vinifera*	Vivi
C_4	*Setaria italica*	Seit
C_4	*Sorghum bicolor*	Sobi
CAM	*Ananas comosus*	Anco
CAM	*Kalanchoë fedtschenkoi*	Kafe
CAM	*Phalaenopsis equestris*	Pheq

Supplementary Table 10. List of *Kalanchoë fedtschenkoi* genes showing convergent changes in protein sequences.

Tribe_id	Name	Gene locus	Definition	Gene expression (FPKM)[a]	Gene Co-expression module[b]	Cluster[c]	Profile-variation[d]
I50_F000807	PEPC2	Kaladp0048s0578.1	Phosphoenolpyruvate carboxylase 2	280.38	MEblack	3	non-flat
I50_F001102	NAP1L4	Kaladp0094s0051.1	Nucleosome assembly protein 14-like	3.4	MEturquoise	NA	flat
I50_F001653	HY5	Kaladp0060s0460.1	Transcription factor hy5-like protein	59.76	MEblack	NA	flat
I50_F003508	GPI	Kaladp0095s0394.1	Glucose-6-phosphate isomerase chloroplastic-like	38.42	MEturquoise	1	non-flat

[a]The maximum expression level in the mature leaf during 24-h period, as revealed by RNA-seq analysis.
[b]The gene co-expression module listed in Supplementary Fig. 9.
[c]Gene clusters constructed, using maSigPro [5, 6], from the transcripts that showed significantly (ANOVA of glm models where H_0 = a flat line, $P<0.05$) "non-flat" diel expression patterns.
[d]The significance of variation of transcript abundance over a 24-h period was determined by a polynomial regression. "Non-flat" indicates that the transcript abundance varied significantly (ANOVA of glm models where H_0 = a flat line, $P<0.05$) across time points. "Flat" indicates that the transcript abundance did not vary significantly (ANOVA of glm models where H_0 = a flat line, $P<0.05$) across time points.

Supplementary Table 11. A list of *Kalanchoë fedtschenkoi* genes involved in stomatal movement. The role of *K. fedtschenkoi* genes was predicted based on homology to the *Arabidopsis* genes that have been shown to be involved in regulation of stomatal movement.

**Name**	***Kalanchoë***	**Description**	**Arabidopsis**	**References**
ABI1	Kaladp0011s0443.1	ABA INSENSITIVE 1	AT4G26080	[9]
ABI2	Kaladp0048s0509.1	ABA INSENSITIVE 2	AT5G57050	[9]
AHA2	Kaladp0008s0304.1	PLASMA MEMBRANE PROTON ATPASE 2	AT4G30190	[10]
AKT1	Kaladp0055s0506.1	POTASSIUM TRANSPORTER 1	AT2G26650	[11]
ALMT9	Kaladp0062s0038.1	ALUMINUM-ACTIVATED MALATE TRANSPORTER 9	AT3G18440	[12]
BAK1	Kaladp0043s0196.1	BRI1-ASSOCIATED RECEPTOR KINASE	AT4G33430	[13]
BLUS1	Kaladp0062s0090.1	BLUE LIGHT SIGNALING1	AT4G14480	[14]
CPK23	Kaladp0040s0351.1	CALCIUM-DEPENDENT PROTEIN KINASE 23	AT4G04740	[15]
CPK3	Kaladp0042s0341.1	CALCIUM-DEPENDENT PROTEIN KINASE 3	AT4G23650	[16]
CPK6	Kaladp0055s0096.1	CALCIUM-DEPENDENT PROTEIN KINASE 6	AT2G17290	[16]
HT1	Kaladp0073s0100.1	HIGH LEAF TEMPERATURE 1	AT1G62400	[17]
KAT1	Kaladp0008s0789.1	POTASSIUM CHANNEL IN ARABIDOPSIS THALIANA 1	AT5G46240	[18]
KAT2	Kaladp0840s0007.1	POTASSIUM CHANNEL IN ARABIDOPSIS THALIANA 2	AT4G18290	[19]
OST1	Kaladp0016s0289.1	OPEN STOMATA 1	AT4G33950	[20]
OST2/AHA1	Kaladp0098s0188.1	OPEN STOMATA 2	AT2G18960	[21]
PHOT1	Kaladp0071s0248.2	PHOTOTROPIN 1	AT3G45780	[22]
PHOT2	Kaladp0033s0113.1	PHOTOTROPIN 2	AT5G58140	[22]
PYL9	Kaladp0008s0082.1	PYRABACTIN RESISTANCE 1-LIKE 9	AT1G01360	[23]
QUAC1/ALMT12	Kaladp0091s0013.1	QUICK-ACTIVATING ANION CHANNEL 1	AT4G17970	[24]
SLAC1	Kaladp0050s0214.1	SLOW ANION CHANNEL-ASSOCIATED 1	AT1G12480	[25]

Supplementary Table 12. A list of *Kalanchoë fedtschenkoi* genes involved in circadian rhythm. The role of *K. fedtschenkoi* genes was predicted based on homology to the *Arabidopsis* genes that have been shown to be involved in circadian rhythm.

**Name**	***Kalanchoë***	**Description**	***Arabidopsis***	**References**
COP1	Kaladp0011s0927.1	CONSTITUTIVE PHOTOMORPHOGENIC 1	AT2G32950	[25]
CRY1	Kaladp0071s0308.1	CRYPTOCHROME 1	AT4G08920	[26]
CRY2	Kaladp0082s0193.1	CRYPTOCHROME 2	AT1G04400	[27]
ELF3	Kaladp0039s0732.1	EARLY FLOWERING 3	AT2G25930	[28]
FKF1	Kaladp0036s0214.1	FLAVIN-BINDING, KELCH REPEAT, F BOX 1	AT1G68050	[29]
PHYA	Kaladp0034s0172.1	PHYTOCHROME A	AT1G09570	[30]
PHYB	Kaladp0039s0298.1	PHYTOCHROME B	AT2G18790	[31]
CCA1	Kaladp0496s0018.2	CIRCADIAN CLOCK ASSOCIATED 1	AT2G46830	[32]
CHE	Kaladp0032s0054.1	CCA1 HIKING EXPEDITION	AT5G08330	[33]
GI	Kaladp0040s0489.1	GIGANTEA	AT1G22770	[34]
LUX	Kaladp0033s0047.1	LUX ARRHYTHMO	AT3G46640	[35]
PIF3	Kaladp0057s0097.1	PHYTOCHROME INTERACTING FACTOR 3	AT1G09530	[36]
PRR3	Kaladp0058s0661.1	PSEUDO-RESPONSE REGULATOR 3	AT5G60100	[37]
PRR5	Kaladp0032s0115.1	PSEUDO-RESPONSE REGULATOR 5	AT5G24470	[38]
PRR7	Kaladp0101s0041.1	PSEUDO-RESPONSE REGULATOR 7	AT5G02810	[38]
PRR9	Kaladp0032s0115.1	PSEUDO-RESPONSE REGULATOR 9	AT2G46790	[38]
TOC1	Kaladp0040s0446.2	TIMING OF CAB EXPRESSION 1	AT5G61380	[39]
ZTL	Kaladp0809s0098.1	ZEITLUPE	AT5G57360	[40]
LNK1	Kaladp0607s0046.1	NIGHT LIGHT-INDUCIBLE AND CLOCK-REGULATED 1	AT5G64170	[41]
LNK2	Kaladp0099s0129.1	NIGHT LIGHT-INDUCIBLE AND CLOCK-REGULATED 2	AT3G54500	[41]
RVE1	Kaladp0574s0015.1	REVEILLE 1	AT5G17300	[42]
RVE6	Kaladp0055s0349.1	REVEILLE 6	AT5G52660	[43]
RVE8	Kaladp0577s0020.1	REVEILLE 8	AT3G09600	[44]
CKB4	Kaladp0016s0180.1	CASEIN KINASE II BETA SUBUNIT 4	AT2G44680	[45]
ELF4	Kaladp0045s0206.1	EARLY FLOWERING 4	AT2G40080	[35]
FIO1	Kaladp0089s0025.1	FIONA1	AT2G21070	[46]
HY5	Kaladp0060s0460.1	ELONGATED HYPOCOTYL 5	AT5G11260	[47]
JMJD5	Kaladp0076s0198.1	LIGHT INSENSITIVE PERIOD1	AT5G64813	[48]
LWD1	Kaladp0048s0797.1	LIGHT-REGULATED WD 1	AT1G12910	[49]
PRMT5	Kaladp0056s0075.1	PROTEIN ARGININE METHYLTRANSFERASE 5	AT4G31120	[50]
SKIP	Kaladp0040s0680.1	SNW/SKI-INTERACTING PROTEIN	AT1G77180	[51]
STIPL1	Kaladp0071s0383.1	SPLICEOSOMAL TIMEKEEPER LOCUS1	AT1G17070	[52]
TEJ	Kaladp0040s0530.1	POLY(ADP-RIBOSE) GLYCOHYDROLASE 1	AT2G31870	[53]

Supplementary Data Files

Supplementary data 1. Biological processes enriched in co-expression modules. See separate Excel file.

Supplementary data 2. The hub genes with the top 1% most connected nodes as well as putative CAM genes with at least 10 directed edges from each gene cluster shown in Supplementary Table 7 and Supplementary Fig. 11. See separate Excel file

Supplementary data 3. List of *Kalanchoë* genes showing convergent changes in diel expression pattern. See separate Excel file

Supplementary data 4. Gene count of CAZyme module genes in thirteen plant species. See separate Excel file.

Supplementary data 5. List of predicted CAZyme genes in *Kalanchoë fedtschenkoi*. See separate Excel file.

Supplementary Notes

Supplementary Note 1

CAM pathway genes in *Kalanchoë*

The CAM pathway can be divided into two temporally separated processes that bring about the photosynthetic assimilation of CO_2: the carboxylation reactions at night that initially capture atmospheric CO_2 and convert it to malic acid, and the subsequent decarboxylation reactions during the day that regenerate this CO_2 and allow its assimilation via the Calvin-Benson cycle. Our metabolite profiling revealed that malic acid accumulation from nocturnal CO_2 fixation peaked during the dark period in mature *K. fedtschenkoi* leaf tissue (Supplementary Fig. 21). *Kalanchoë* species were instrumental in early biochemical studies that established the basic pathways of carbon flow during the CAM cycle [54], and more recent studies have identified several key regulatory steps involved in controlling flux through these pathways [55]. Key components of the nocturnal carboxylation process include beta-carbonic anhydrase (β-CA), phospho*enol*pyruvate carboxylase (PEPC), phospho*enol*pyruvate carboxylase kinase (PPCK), NAD(P)-malate dehydrogenase (MDH), and the tonoplast membrane proteins responsible for the vacuolar accumulation of malic acid, which include the two energy-dependent H^+ pumps, the V-ATPase, and V-PP_iase, and the inward-rectifying malate transporters such as aluminum-activated malate transporter (ALMT). In the subsequent light period, malic acid is remobilized from the vacuole by vacuolar malate transporters such as tonoplast dicarboxylate transporter (TDT) and decarboxylated in *Kalanchoë* via NAD(P)-malic enzyme (ME) and is further converted to phospho*enol*pyruvate (PEP) by pyruvate phosphate dikinase (PPDK), which is activated in the light through dephosphorylation by PPDK regulatory protein (PPDK-RP), and CO_2 is released for assimilation by RuBisCO in the Calvin cycle (Fig. 3a). Transcriptome sequencing (RNA-seq) data was generated from mature CAM leaves of *K. fedtschenkoi* that were collected at 2 h intervals over a 12h light/12h dark cycle. Analysis of the resulting light/ dark cycles of transcript abundance profiles revealed candidate gene family members corresponding to each of the proposed core components of the CAM cycle (Supplementary Tables 5 and 6).

Gene duplication can be a major source of genetic novelty driving evolutionary innovation in plants [56]. Several CAM-related gene families in *K. fedtschenkoi*, including *PEPC*, *MDH*, *ALMT*, *NAD-ME*, and *NADP-ME* families, contain genes from recent genome duplication events, with 4DTV values ranging from 0.11 – 0.20 (Fig. 3a). Furthermore, differences in both transcript abundance and diel expression patterns between duplicated genes were identified (Fig. 3b). These results suggest that both recent WGD and functional diversification of genes by reprogramming of diel gene expression after duplication have contributed to the evolution of CAM-related gene families. This contrasts strikingly with the scenario inferred from the analysis of CAM gene evolution in the pineapple genome, where CAM-recruited genes were not found to have occurred through duplication and neofunctionalization, but instead the ancestral copy of each gene shared with grasses was simply elevated at the transcript level in CAM tissues, and in some cases also placed under light/dark and potentially circadian clock control [57].

Nine *β-CA* genes are predicted in the *K. fedtschenkoi* genome (Supplementary Fig. 22a), among which three genes (Kaladp0095s0400, Kaladp0018s0287, and Kaladp0018s0289) have high transcript abundance (Supplementary Fig. 22b). The transcript expression of Kaladp0018s0287 and Kaladp0018s0289 showed pronounced diel changes (Supplementary Table 5), reaching peak abundance towards the middle of the night (Supplementary Fig. 22b). Because carboxylation of PEP by PEPC at night depends on the initial hydration of CO_2 by β-

CA, a CAM-specific role for this isoform of β-CA in supporting nocturnal carbon fixation is implied. In addition, the peak transcript abundance of Kaladp0095s0400 occurred in the late afternoon (Supplementary Fig. 22b), indicating that this gene might also be relevant to CAM under the assumption that protein expression is delayed relative to transcript expression. The relative importance of Kaladp0018s0287, Kaladp0018s0289, and Kaladp0095s0400 in nocturnal carbon fixation needs further investigation using genetic approaches (i.e., single and double mutants) in the future.

Five genes in the *K. fedtschenkoi* genome are predicted to encode PEPC (Supplementary Fig. 23a), which is one of the most abundant soluble proteins in CAM plants [58]. Among these, Kaladp0095s0055 was by far the most abundant transcript and it displayed a clear light/dark rhythm in transcript abundance with a peak during the late afternoon just before the start of the dark period (Supplementary Fig. 23b), making this gene a strong candidate for the CAM-specific member of the PEPC gene family. Kaladp0048s0578 was the second most abundant PEPC transcript among the five PEPC genes, with transcript abundance peaking around midnight (Supplementary Fig. 23b). Therefore, both Kaladp0095s0055 and Kaladp0048s0578 might contribute to nocturnal carbon fixation in *K. fedtschenkoi*.

The *K. fedtschenkoi* genome contains four *PPCK* genes (Kaladp0037s0517, Kaladp0050s0014, Kaladp0604s0001, and Kaladp0082s0192; Supplementary Fig. 24a) that are candidates for encoding the protein kinase that catalyzes the phosphorylation of PEPC. Kaladp0037s0517 corresponds to the *K. fedtschenkoi* gene that was the first to be characterized as encoding the functional PPCK in any CAM plant [59]. This gene showed the highest transcript abundance, and displayed a transcript abundance peak in the middle of the dark period (Supplementary Fig. 24b). These results indicate that Kaladp0037s0517 is the CAM-specific, nocturnally active PPCK gene.

The 15 MDH genes predicted in the *K. fedtschenkoi* genome can be divided into four tribes: tribe MDH1 comprising three genes (Supplementary Fig. 25a), tribe MDH2 comprising eight genes (Supplementary Fig. 26a), tribe MDH3 (Supplementary Fig. 27a) comprising three genes, and tribe MDH4 (Supplementary Fig. 27c) comprising one gene from *K. fedtschenkoi*. The transcript abundance varies among these genes (Supplementary Figs. 25b, 26b, 27b and 27d). Among these MDH genes, Kaladp0082s0194 (in tribe MDH1) had the highest transcript abundance and showed a peak transcript level in the late afternoon before the onset of the dark period (Supplementary Fig. 25b), temporally similar in phase to the expression of the *PEPC* gene Kaladp0095s0055 (Supplementary Fig. 23b). Kaladp0001s0257 (in tribe MDH2) had the second-most transcript abundance of MDH and also displayed a similar diel pattern of gene expression, with transcript levels peaking before dusk (Supplementary Fig. 26b).

Malate influx into the vacuole in *Kalanchoë* occurs via an anion-selective inward-rectifying ion channel [60]. The ALMT genes predicted in the *K. fedtschenkoi* genome can be divided into two tribes (Supplementary Figs. 28a and 29a) and there are variations in transcript abundance among these genes (Supplementary Figs. 28b and 29b). Among these ALMT genes, Kaladp0073s0021 is the most highly expressed and its transcript shows a strong diel change in abundance, with peak transcript occurring in the early part of the light period (Supplementary Fig. 28b). Another ALMT gene, Kaladp0062s0038, has lower transcript abundance than Kaladp0073s0021, but it has peak expression in the middle of the dark period (Supplementary Fig. 28b). Both genes are orthologous to the *ALMT4/5/6* clade of *A. thaliana* genes, which includes *ALMT6*, a tonoplast-localized malate channel that mediates voltage-gated malate influx into the vacuole [61]. With the onset of the light period, malate efflux from the vacuole begins as

the nocturnally accumulated malic acid is remobilized as substrate for internal CO_2 release. The strong inward-rectification of the vacuolar malate channel suggests that a separate class of transport protein may be involved in malate export [62]. One candidate is the *K. fedtschenkoi* ortholog (Kaladp0042s0251) of the *Arabidopsis* TDT protein [63], which showed a clear diel oscillation in transcript abundance (Supplementary Table 6), peaking during the first 4 hours of the light period (Supplementary Fig. 30).

Upon release of malate from the vacuole back into the cytoplasm, malate decarboxylation in the light phase of CAM in *Kalanchoë* is brought about by ME [64, 65, 66]. The alternative decarboxylase PEP carboxykinase (PEPCK) showed relatively low transcript abundance (Supplementary Table 6). Among the 13 *ME* family genes predicted in the *K. fedtschenkoi* genome (Supplementary Fig. 31a), Kaladp0092s0166 (*NADP-ME*) showed the highest transcript level, with abundance peaking at dawn (Supplementary Fig. 31b). PPDK, which converts pyruvate to PEP and initiates the gluconeogenic recovery of storage carbohydrate starch, was represented by only a single full-length gene in *K. fedtschenkoi* (Kaladp0076s0229). Kaladp0076s0229 showed a strong light/dark cycle of transcript abundance, with peak transcript level phased to dawn (Supplementary Fig. 32), consistent with the predicted function of this enzyme during pyruvate recycling to starch in the light period [65, 66]. Two genes (Kaladp0010s0106 and Kaladp0060s0363) in the *K. fedtschenkoi* genome are predicted for PPDK-RP, both of which showed higher transcript levels during the light period relative to the dark, and Kaladp0060s0363, in particular, displayed a peak transcript abundance phased to dusk (Supplementary Fig. 33), consistent with the regulatory phosphorylation of PPDK in the dark to inactivate it [65].

Supplementary Note 2
Convergent evolution of genes in secondary metabolism

Secondary metabolism plays an important role in plant-environmental interactions. Plants synthesize various types of secondary metabolites, such as phenylpropanoids, glucosinolates, terpenoids, and phytoalexins/alkaloids [67]. Among the genes showing convergent changes in patterns of diel expression in two CAM species (*K. fedtschenkoi* and *A. comosus*) compared with a C_3 species (*A. thaliana*) (Supplementary Data 3), two (Kaladp0022s0177 and Kaladp0043s0207) were predicted to be involved in multiple secondary metabolic processes, including jasmonic acid biosynthesis (Supplementary Fig. 34), and aromatic amino acid biosynthesis via the shikimate pathway (Supplementary Fig. 35). Kaladp0043s0207 was classified into the co-expression module MEdarkgrey (Supplementary Data 3), which was associated with the leaf samples collected during the last two hours of the dark period (Supplementary Fig. 9), with higher transcript abundance than other diel time periods (Supplementary Fig. 10). Kaladp0022s0177 was classified into the co-expression module MEblack (Supplementary Data 3), which was associated with leaf samples collected 2-6 h after the beginning of the dark period (Supplementary Fig. 9), with higher transcript abundance than other diel time periods (Supplementary Fig. 10). The diel transcript abundance profiles of Kaladp0043s0207 and Kaladp0022s0177 and their corresponding *A. comosus* orthologs clustered together, distinct from the diel transcript abundance of their *Arabidopsis* ortholog (Supplementary Fig. 36). There was only a 1-h shift in diel transcript abundance pattern of Kaladp0043s0207 relative to its *A. comosus* ortholog Aco001151, whereas Kaladp0043s0207 and Aco001151 showed 12- and 11-h shifts relative to their best-matched *A. thaliana* ortholog AT2G21940, respectively (Supplementary Fig. 36a). The diel abundance of transcript of

Kaladp0022s0177 displayed only 1-h shift relative to its *A. comosus* ortholog Aco010785, whereas Kaladp0022s0177 and Aco010785 showed 8- and 7-h shifts relative to their best-matched *A. thaliana* ortholog AT4G29010, respectively (Supplementary Fig. 36b). Kaladp0043s0207 encodes shikimate kinase, an enzyme in the shikimate pathway that phosphorylates the C_3 hydroxyl group of shikimate to yield shikimate-3-phosphate, possibly providing a regulatory link between the energy-requiring shikimate pathway and cellular energy balance in plants [68]. Kaladp0022s0177 encodes the fatty acid beta-oxidation multifunctional protein AIM1/MFP2, which is involved in the biosynthesis of jasmonic acid (JA), an important regulator of plant development and stress response [69]. Because methyl jasmonate (MeJA), derived from JA, has a role in the induction of stomatal closure in various plant species [70], this gene might influence the regulation of stomatal movement in CAM species.

Supplementary Methods

Supplementary Method 1

Analysis of positive selection in genes in CAM species

The protein-coding sequences of 24 plant species including *Amborella trichopoda* (PLAZA 3.0 [71]; available at http://bioinformatics.psb.ugent.be/plaza/), *Ananas comosus* [72], *Arabidopsis thaliana* (PLAZA 3.0), *Beta vulgaris* (PLAZA 3.0), *Brachypodium distachyon* (PLAZA 3.0), *Carica papaya* (PLAZA 3.0), *Citrus sinensis* (PLAZA 3.0), *Eucalyptus grandis* (PLAZA 3.0), *Fragaria vesca* (PLAZA 3.0), *Kalanchoë fedtschenkoi* (v1.1; available at https://phytozome.jgi.doe.gov), *Medicago truncatula* (PLAZA 3.0), *Mimulus guttatus* (PLAZA 3.0), *Musa acuminata* (PLAZA 3.0), *Oryza sativa* (PLAZA 3.0), *Phalaenopsis equestris* [72], *Populus trichocarpa* (PLAZA 3.0), *Prunus persica* (PLAZA 3.0), *Setaria italica* (PLAZA 3.0), *Solanum lycopersicum* (PLAZA 3.0), *Solanum tuberosum* (PLAZA 3.0), *Sorghum bicolor* (PLAZA 3.0), *Theobroma cacao* (PLAZA 3.0), *Vitis vinifera* (PLAZA 3.0), *Zea may* (PLAZA 3.0) were used for positive selection analysis. The longest protein-coding sequence was selected in case of multiple transcripts annotated for one gene locus. The protein-coding sequences were used for discovery of genes under positive selection in each of the CAM species in comparison with the non-CAM species using PosiGene, which is an automated pipeline for genome-wide detection of positively selected genes [8], with default settings. In this positive selection analysis through the PosiGene pipeline, *K. fedtschenkoi* was used as the reference species for ortholog assignment and each of the three CAM species (i.e., *K. fedtschenkoi*, *A. comosus*, *P. equestris*) was used as anchor species.

Supplementary Method 2

Plant material

The plants were grown in soil under a mixture of fluorescent incandescent lamps providing a photon flux density of 250 µmol m^{-2} s^{-1} on a 12h light (25°C)/12h dark (18°C) cycle, and day/night relative humidities of 45/75%. In order to capture mRNA abundance changes responsive to diel conditions, samples were collected in triplicate from leaves every 2h over a 24h time course under 12h light/12h dark (L/D; Diel time). Additional tissues were sampled in triplicate including roots, flowers, shoot tips plus young leaves, and stems and frozen in liquid nitrogen until RNA isolation.

Supplementary Method 3

Chromosome counting

Kalanchoë plantlets were grown on MS30 media (MS Modified Basal Medium supplemented with Gamborg's B5 vitamins pH 5.8, 3% (w/v) sucrose, 0.7% (w/v) Phytoagar) under a 16 h light/8 h dark cycle at 22°C, resulting in root production for mitotic chromosome preparation. Root tips were excised and treated in ice-cold distilled water for 24 h to induce mitotic arrest. Root tips were fixed in 3:1 ethanol and glacial acetic acid (v/v) for 24 h at room temperature and stored at 4 °C until used. After rinsing root tips in distilled water, root tips were transferred to microfuge tubes containing 50 µl of enzyme solution containing 1% (w/v) Pectolyase Y-23 (MP) and 2% (w/v) Cellulase (BioWorld, Dublin, OH, USA) in citrate buffer (10 mM sodium citrate, 10 mM sodium EDTA, pH 5.5) for 1 h at 37 °C. Tubes were transferred

to ice and washed three times with 70% ethanol. Root tips were macerated in the residual approximately 100 µl of liquid using a dissecting probe and centrifuged at 2000 x g for 5 sec or less. The supernatant was decanted and the tubes were inverted on a paper towel to dry. Each cell pellet was resuspended in 35 µl glacial acetic acid at room temperature and 10 µl of the cell suspension was applied on glass microscope slides and dried in a humidified chamber. Once the solvent had evaporated, one drop of Fluoroshield with DAPI histology mounting medium (Sigma-Aldrich, St. Louis, MO, USA) was applied per slide to dye chromosomes.

Supplementary Method 4

Illumina sequencing of genome

To isolate nuclear DNA for genome sequencing, fresh tissues of young leaves were collected, ground in liquid nitrogen, and stored at −80°C until use. Intact nuclei from ground tissues were recovered in Sucrose-based Extraction Buffer as described [73]. The genomic DNA was extracted by a DNeasy plant maxi-kit (Qiagen, Valencia, CA, USA). TruSeq DNA PCR-Free Library Prep kit (Illumina, CA, USA) was used to generate the paired-end sequencing libraries with an average insert size around 540 bp. The mate-pair libraries with an average insert size of 3, 6, and 11 kb, respectively, were made using the Nextera Mate Pair Sample Prep Kit (Epicentre, WI, USA).

Supplementary Method 5

Transcriptome sequencing

A total of 48 samples including 36 mature leaf tissue samples (= 12 time-points × 3 biological replicates) and 12 other tissue samples (= 4 tissue types × 3 biological replicates) were collected. The plant samples were frozen in liquid nitrogen, ground using a mortar and pestle, and frozen at −80°C until use. For total RNA isolation, approximately 100mg of frozen ground tissue was incubated in 850 µl of CTAB buffer (1.0% β-Mercaptoethanol) at 56°C for 5 minutes, 600 µl chloroform:isoamylalcohol (24:1) was added and samples were spun at full speed for 8 min. The supernatant (~730 µl) was removed from the top layer and applied to a filter column provided in the Spectrum™ Plant Total RNA Kit (Sigma, Cat. No. STRN250-1KT). RNA was precipitated in 500 µl of 100% ethanol and applied to a Spectrum kit binding column. The protocol provided by the Spectrum kit was followed from that point on. An on-column DNase treatment was done to rid the samples of residual genomic DNA. RNA quantity and quality was quantified using a NanoDrop 1000 spectrophotometer (Thermo Scientific, Wilmington, DE, USA) and 3 µg of each sample was aliquoted for transcriptomics analysis. Total RNA samples were sent to HudsonAlpha Institute for Biotechnology (Huntsville, AL, USA) for library prep and sequencing. After passing quality checks, libraries were constructed using the Illumina TruSeq Stranded mRNA library prep kit (Illumina Inc., San Diego, CA). Each library was quantified using a QuBit, average fragment size was determined using an Agilent High Sensitivity Chip and qPCR was used to approximate optimal loading concentrations. Libraries were loaded onto 8 channels of an Illumina HiSeq V4 flowcell and paired end sequencing (2×150 bp) was used to generate reads of ~20 million bp for each sample on an Illumina HiSeq 2500 instrument (Illumina, CA, USA). Raw fastq files were provided for downstream analysis.

Supplementary Method 6

Protein-coding gene annotation

Repeat library was generated by RepeatModeler [74] from *K. fedtschenkoi* assembly. 50,414 transcript assemblies (TAs) were made from ~414 M paired-end reads by PERTRAN (Shu, unpublished). The TAs and ESTs were further assembled into PASA transcript assemblies (46,382), and 185,671 TAs and ESTs into sibling PASA transcript assemblies (62,501) by PASA [75]. Loci were determined by PASA transcript assembly alignments and/or EXONERATE alignments of proteins from Arabidopsis thaliana, rice, sorghum, mimulus, grape, orchid (*Phalaenopsis equestris*) and Swiss-Prot proteomes to repeat-soft-masked *K. fedtschenkoi* genome using RepeatMasker [76] with up to 2 K bp extension on both ends unless extending into another locus on the same strand. Gene models were predicted by homology-based predictors, FGENESH+ [77], FGENESH_EST (similar to FGENESH+, EST as splice site and intron input instead of protein/translated ORF), and GenomeScan [78]. The best scored predictions for each locus are selected using multiple positive factors including EST and protein support, and one negative factor: overlap with repeats. The selected gene predictions were improved by PASA. Improvement includes adding UTRs, splicing correction, and adding alternative transcripts. PASA-improved gene model proteins were subject to protein homology analysis to above mentioned proteomes to obtain Cscore and protein coverage. Cscore is a protein BLASTP score ratio to MBH (mutual best hit) BLASTP score and protein coverage is highest percentage of protein aligned to the best of homologs. PASA-improved transcripts were selected based on Cscore, protein coverage, EST coverage, and its CDS overlapping with repeats. The transcripts were selected if its Cscore is larger than or equal to 0.5 and protein coverage larger than or equal to 0.5, or it has EST coverage, but its CDS overlapping with repeats is less than 20%. For gene models whose CDS overlaps with repeats for more than 20%, its Cscore must be at least 0.9 and homology coverage at least 70% to be selected. The selected gene models were subject to Pfam and Panther analysis and gene models whose protein is more than 30% in Pfam/Panther TE domains were removed.

Supplementary Method 7

Construction of orthologous groups

The protein sequences of 26 plant species including *Amborella trichopoda* (PLAZA 3.0 [71]; available at http://bioinformatics.psb.ugent.be/plaza/), *Ananas comosus* [72], *Aquilegia coerulea* (v1.1; available at https://phytozome.jgi.doe.gov), *Arabidopsis thaliana* (PLAZA 3.0), *Beta vulgaris* (PLAZA 3.0), *Brachypodium distachyon* (PLAZA 3.0), *Carica papaya* (PLAZA 3.0), *Citrus sinensis* (PLAZA 3.0), *Eucalyptus grandis* (PLAZA 3.0), *Fragaria vesca* (PLAZA 3.0), *Kalanchoë fedtschenkoi* (v1.1; available at https://phytozome.jgi.doe.gov), *Medicago truncatula* (PLAZA 3.0), *Mimulus guttatus* (PLAZA 3.0), *Musa acuminata* (PLAZA 3.0), *Oryza sativa* (PLAZA 3.0), *Phalaenopsis equestris* [72], *Physcomitrella patens* (PLAZA 3.0), *Populus trichocarpa* (PLAZA 3.0), *Prunus persica* (PLAZA 3.0), *Setaria italica* (PLAZA 3.0), *Solanum lycopersicum* (PLAZA 3.0), *Solanum tuberosum* (PLAZA 3.0), *Sorghum bicolor* (PLAZA 3.0), *Theobroma cacao* (PLAZA 3.0), *Vitis vinifera* (PLAZA 3.0), *Zea may* (PLAZA 3.0) were used for ortholog group construction. The longest protein sequence was selected in case of multiple transcripts annotated for one gene locus. The ortholog groups (OGs) were constructed using FastOrtho (http://enews.patricbrc.org/fastortho/), a reimplementation of the OrthoMCLprogram

[79], with default parameters (except a BLASTp E-value cutoff of 1e-5 and an inflation value of 1.3).

Supplementary Method 8

Construction of species phylogeny

Specifically, for each ortholog group of the single-copy genes, the protein sequences were aligned using MAFFT software version 7.221 [80], using an accurate option (L-INS-i). The coding sequences (CDS) of each ortholog group were aligned using PAL2NAL [81], guided by their corresponding protein sequence alignments. The species phylogeny was reconstructed from the protein or CDS alignments using the following three strategies:

Concatenated supermatrix strategy: The protein sequence alignments of these 210 single-copy-gene ortholog groups were then concatenated into one supermatrix file using FASconCAT [82]. The concatenated supermatrix file was used to infer maximum-likelihood phylogenetic tree using IQ-TREE version 1.4.3 [1], with automatic model selection (-m TEST) and ultrafast bootstrap (-bb 1000) [83].

Partitioned analysis of multi-gene alignment strategy: A concatenated protein sequence alignment with gene partition was generated for the 210 single-copy-gene ortholog groups using FASconCAT-G_v1.02 [84], with output in two different formats: the NEXUS format that was imbedded with MrBayes commands for Bayesian inference of species tree) and the FASTA format along with a partition file containing the best-fit models of amino acid replacement selected by using ProtTest version 3.4.2 [85]. The partitioned protein sequence alignment in NEXUS format was used for Bayesian inference of species tree using MrBayes version 3.2.6 [2], with nst=6, rates=invgamma, mcmc ngen=10,000,000, printfreq=1000, samplefreq=1000. The run was stopped at 4,530,000 MCMC steps when the max standard deviation of split frequencies was 0.004577. Also, the partitioned protein sequence alignment in FASTA format along with a partition file containing the best-fit models of amino acid replacement was used for maximum likelihood inference of species tree using IQ-TREE version 1.4.4 [1], with ultrafast bootstrap (-bb 1000) [83].

Coalescent-based species tree strategy: The CDS alignments for each of the 210 single-copy-gene ortholog groups were used to reconstruct the multispecies coalescent tree using starBEAST2 [3] based on Bayesian inference of individual gene trees. Specifically, the site, clock and tree substitution models of these gene sets at all loci were unlinked. The uncorrelated lognormal relaxed clock model was applied in the simulations with the clock rate setting as 1.0, gene ploidies of 2.0 for all genes, and a constant population IO model. The population shape was set as 3 with an estimated mean of 1.0. The HKY+Γ site model with four gamma categories and estimated substitute rate and population invariant was chosen. The Markov chain Monte Carlo (MCMC) method was applied for 12 million states, and trees of the simulation were stored every five thousand states, resulting 2,400 trees at each gene locus. The tree evaluation was performed using ASTRAL-II [4] on the combination of the last 1,000 trees at all gene loci with 400 bootstrap replicates.

Also, individual maximum-likelihood gene trees were reconstructed from the CDS alignments for each of the 210 single-copy-gene ortholog groups using RAxML [86] with 500 bootstrap replicates. The gene trees were used to create a species tree using ASTRAL-II [4] analysis. Specifically, a coalescent species tree was first estimated by providing the best trees output from RAxML as well as all the bootstrap replicates. The ASTRAL-II analysis was run for 400 bootstraps, and the final "Astral" tree was treated as the species tree estimate. To estimate

quartet frequency support at each node and posterior probabilities, ASTRAL-II was re-run using a correctly rooted tree (at *Physcomitrella patens*) using the -q and -t 2 options.

Supplementary Method 9

Phylogenetic analysis of protein tribes

For phylogenetic analysis, the protein tribes that contain protein sequences from the 13 plant species listed in Supplementary Table 9 were used for analysis of convergent changes in protein sequences in CAM species. Specifically, the protein sequences of the 13 plant species in each tribe were aligned using MAFFT software version 7.221 [80], using an accurate option (L-INS-i). The protein sequence alignment was used to infer maximum-likelihood phylogenetic tree using IQ-TREE [1], with automatic model selection (-m TEST) and ultrafast bootstrap (-bb 1000) [83].

Supplementary Method 10

Analysis of convergence in protein sequences in CAM species

The tribes that contain at least one "CAM-convergence" clade in phylogenetic tree were used to identify convergent changes in protein sequence. Specifically, representative protein sequence (one sequence per species) was selected for each tribe containing "CAM-convergence" clade. The representative protein sequence for each tribe were aligned using MAFFT software version 7.221 [80], using an accurate option (L-INS-i). The protein sequence alignments and the species tree were used as input for the Grand-Convergence software [87, 88, 89], which estimates the posterior numbers of convergent and divergent substitutions shared between all pairs of branches in the given phylogeny, to identify the convergent amino acid changes. The convergent sites (with probability > 0.99) predicted by the Grand-Convergence software were manual examined to identify amino acids mutations shared by a *K. fedtschenkoi* gene and its ortholog in at least one of the two monocot CAM species, as compared with C_3 and C_4 species. To check whether the mutations exist in any non-CAM species in the public databases, the *K. fedtschenkoi* protein sequences containing the mutation sites were used to search against the NCBI protein blast database (https://blast.ncbi.nlm.nih.gov/Blast.cgi), the Phytozome protein blast database (https://phytozome.jgi.doe.gov), and the 1KP project protein blast database (https://www.bioinfodata.org/Blast4OneKP/) using a blastp e-value of 1e-5 and a maximum target number cutoff of 20,000. Then the protein sequences of the blast hits were searched for mutation sites using blastp with the *K. fedtschenkoi* mutation-containing sub-sequences (9-19 amino acids in length with the mutation sites in the middle) as queries and a blastp e-value of 10. As such, a *K. fedtschenkoi* gene is defined to be involved in convergent evolution in protein sequence if meeting the following three criteria: 1) the *K. fedtschenkoi* gene is placed together with gene(s) from at least one of the two monocot CAM species (*A. comosus* and *P. equestris*) in a phylogenetic clade that does not contain any genes from C_3 or C_4 species; 2) convergent amino-acid changes were detected (with probability > 0.99) by the Grand-Convergence software [87, 88] between the *K. fedtschenkoi* gene with gene(s) from at least one of the two monocot CAM species (*A. comosus* and *P. equestris*); 3) the *K. fedtschenkoi* gene shares at least one amino acid mutation with its ortholog in at least one of the two monocot CAM species, as compared with C_3 and C_4 species.

Supplementary Method 11

Gene Ontology analysis and pathway annotation

The enrichment of GO biological process was performed using ClueGO [90], with the right-sided hypergeometric enrichment test at medium network specificity selection and *p*-value (hypergeometric enrichment test) correction was performed using the Holm-Bonferroni step-down method [91]. GO-term fusion and grouping settings were selected to minimize GO-term redundancy and the term enriched at the highest level of significance was used as the representative term for each GO-term group. The GO-terms with corrected $P<0.05$ (hypergeometric enrichment test) were considered significantly enriched. Pathway annotation for the protein sequences was performed on the KEGG Automatic Annotation Server KAAS [92], using the BBH (bi-directional best hit) method to assign orthologs.

Supplementary Method 12

Analysis of co-expression network in *Kalanchoë*

The primary transcripts with an average abundance (calculated from three biological replicates) of greater than 1 FPKM in at least one of the 16 samples were utilized to construct a weighted gene co-expression network using the R package WGCNA [93]. The gene expression data were log2 transformed. Modules were constructed using the following parameters: maxBlockSize=10000, power=9, networkType="unsigned", mergeCutHeight=0.25, minModuleSize=30, corType = "bicor". "bicor" represents the biweight midcorrelation, which is a robust correlation measure for co-expression network analysis [94].

Supplementary Method 13

Cluster analysis of gene expression in *Kalanchoë*

Polynomial regressions of degree=4 were calculated for all transcripts using all 12 time points (i.e., 2, 4, 6, …, 24 hours after the beginning of the light period) using counts=TRUE flag and a Bonferroni-Holm correction with $P<0.05$ (ANOVA of glm models where H_0 = a flat line). Genes marked as influential by the T.fit() function were removed, and regressions were recalculated; only genes that were significantly "non-flat," or genes that could be explained via a polynomial regression, were used for subsequent analyses. Flat genes, or those that did not vary significantly across time points, were ignored. Clustering was done using the "mfuzz" option in maSigPro [5, 6], with the number of clusters being chosen by minimizing the within group sum of squares for *k*=1:20. A *k* of 11 coincided a maximum reduction of within group sum of squares, with no further reductions for *k*>11. Mfuzz clustering also requires a user-defined *m* "fuzzification parameter," which was chosen using the mestimate() function in the Mfuzz R package [95]. An *m* of 1.06 was used for the *K. fedtschenkoi* expression data.

Networks for each cluster were constructed via ARACNE [7]. Transcripts were annotated as transcription factors using PlantTFcat [96], and a list of these TFs were given to ARACNE for each cluster. ARACNE networks are built based on mutual information (MI) indices between genes – how much does one gene explain another. P-values are calculated based on the distribution of MI values in a network. Networks were therefore filtered for only interactions that had a p-value of < 0.01 (as calculated by ARACNE). Networks were displayed in Cytoscape [97], and analyzed using the built-in Network Analysis feature. The size of each node was set to indicate the number of directed edges, with a minimum of 10 edges required for the node to be

visualized. We highlighted the top 1% most connected nodes, as well as canonical CAM genes with at least 10 directed edges.

Supplementary Method 14
Comparative analysis of gene expression

To calculate the time-delay between time-course gene expression profiles, the diurnal expression data with two-hour intervals were further adjusted to one-hour interval time series by interpolation using the cubic spline function in MATLAB (Mathworks, Inc.). The expression data were then normalized by Z-score transformation. Pairwise correlation was calculated between each pair of orthologous genes for all possible time delays using the circular cross correlation function (cxcorr) in MATLAB (Mathworks, Inc.). The cxcorr function produces a correlation coefficient between gene 1 and shifted, or lagged, copies of gene 2 as a function of the lag. With each correlation coefficient, a lag value was given. The lag values were then converted into hours, giving an estimate on time delay. The time delay at which the correlation was maximum was selected as the estimated delay between the two genes.

To compare transcript expression between different time-windows, enrichment triangles were constructed to identify cross-species triangles within each ortholog group, in which a *K. fedtschenkoi* gene and a pineapple ortholog had significantly enriched expression in the same time-window, and an *Arabidopsis* ortholog of these genes had significantly enriched expression in the opposite time-window. Specifically, two types of comparison were performed: midday (4, 6 and 8 h from the starting of the light period) vs. midnight (16, 18 and 20 h from the starting of the light period) and dawn (22, 24 and 2 h from the starting of the light period) vs. dusk (10, 12 and 14 h from the starting of the light period), as illustrated in Figure 4a. Any genes that, after preprocessing, had any negative expression values were removed from further analysis. Each gene expression vector X consisted of gene expression measurements at 12 time points (2h, 4h, 6h, 8h, 10h, 12h, 14h, 16h, 18h, 20h, 22h, 24h). Each entry x_i of each gene expression vector X was then transformed as follows:

$$x_i^* = int\left(\left(\frac{x_i}{\sum_j x_j}\right) \times 1000\right)$$

Essentially, this normalizes each entry of the vector by dividing each entry by the sum of the vector, multiplying the normalized entry by 1000 and taking the integer of the result. This ensures that the values of the transformed vector have whole number values between 0 and 1000. For each species, we then create two-column matrices, namely a midday-midnight matrix and a dawn-dusk matrix for each species. The midday-midnight matrix is constructed such that rows represent genes from a particular species and columns represent light states. One column represents the sum of all transformed midday time points for a given gene and the other column represents the sum of all transformed midnight time points for a given gene. Similarly, we construct a dawn-dusk matrix for each species, in which rows represent genes from the particular species and columns represent either the sum of all dawn time points or the sum of all dusk time points.

For each gene i, we used the right-tailed Fisher Exact Test to determine if that gene's expression was enriched in midday or midnight (or neither) and dusk or dawn (or neither) according to the contingency tables in Supplementary Figure 37, where d_{xy} refers to element xy

in the dawn-dusk matrix D, and m_{xy} refers to element xy in the midday-midnight matrix M. This made use of the Text::NSP::Measures::2D::Fisher Perl module [98] available from the Comprehensive Perl Archive Network (http://www.cpan.org/). The False Discovery Rate [99] was controlled per species and light comparison (midday/midnight or dawn/dusk) at a level less than 0.01. For a given comparison (midday *vs*. midnight or dawn *vs*. dusk), triangles were identified within families as groups of 3 orthologous genes, (a *Kalanchoë* gene, a pineapple gene and an *Arabidopsis* gene) where the *Kalanchoë* gene and the pineapple genes were enriched in the same light state (e.g., midday) and the *Arabidopsis* gene was enriched in the opposite light state (e.g., midnight). The resulting triangles were visualized as networks in Cytoscape [97].

Supplementary Method 15

Genome synteny analysis

For each pairwise alignments, the coding sequences of predicted gene models are compared to each other using LAST [100]. Our synteny search pipeline defines syntenic blocks by chaining the LAST hits with a distance cutoff of 20 genes apart, also requiring at least four gene pairs per synteny block. For homologs inferred based on the syntenic alignments, we aligned the protein sequences using CLUSTALW [101] and used the protein alignments to guide coding sequence alignments by PAL2NAL [81]. To calculate *Ks*, we used the Nei-Gojobori method implemented in *yn00* program in PAML package [102]. In-house Python scripts (https://github.com/tanghaibao/bio-pipeline/tree/master/synonymous_calculation) were used to pipeline all the calculations. MCScanX [103] were used to detect gene synteny and collinearity for seven dicot species including *Arabidopsis thaliana* (PLAZA 3.0), *Carica papaya* (PLAZA 3.0), *K. fedtschenkoi* (v1.1; https://phytozome.jgi.doe.gov), *Populus trichocarpa* (PLAZA 3.0), *Solanum lycopersicum* (PLAZA 3.0), *Theobroma cacao* (PLAZA 3.0), and *Vitis vinifera* (PLAZA 3.0). Four-fold transversion substitutions (4dtv) between syntentic gene pairs were calculated as described previously [104].

Supplementary Method 16

Gas chromatography-mass spectrometry metabolite profiling

The plant samples were frozen in liquid nitrogen and ground using a mortar and pestle, and frozen at −80°C until analyzed. Approximately 100 mg FW of each sample was subsequently twice extracted with 2.5 mL 80% ethanol overnight and then combined prior to drying a 1.0 mL aliquot in a nitrogen stream. Sorbitol was added before extraction as an internal standard to correct for differences in extraction efficiency, subsequent differences in derivatization efficiency and changes in sample volume during heating. Dried extracts were silylated for 1 h at 70ºC to generate trimethylsilyl (TMS) derivatives, which were analyzed after 2 d with an Agilent Technologies Inc. (Santa Clara, CA, USA) 5975C inert XL gas chromatograph-mass spectrometer as described elsewhere [105]. Metabolite peak extraction, identification, and quantification were as described previously [58, 105], and unidentified metabolites were denoted by their retention time as well as key mass-to-charge (m/z) ratios. TCA cycle organic acids, sugars, and abundant secondary metabolites are known or thought to be under diurnal regulation were the focus of this study.

Supplementary Method 17

***In vitro* protein expression and analysis of enzyme activity**

The full-length coding sequences of phosphoenolpyruvate carboxylase (PEPC) genes were synthesized by IDT (https://www.idtdna.com/site), and were then inserted into the bacterial expression vector pGEX6P1 with restriction digestion *Bam*H *I*. The bacterial BL21strains (Novagen BL21 (DE3) pLysS Singles), which carry the plasmids (including wild-type and mutants), were used for target protein induction with IPTG, and the expressed proteins were then purified using glutathione sepharose 4B beads (GE Healthcare Life Sciences, Pittsburgh, PA, USA). After cleavage of GST with PreScission Protease (GE Healthcare Life Sciences, Pittsburgh, PA, USA), the final target protein concentrations were determined by using Qubit fluorometer 2.0 (Invitrogen, Carlsbad, CA, USA). The protein quality was checked via Western blot. Approximately 1 µg purified proteins were used for running with 10% SDS-Polyacrylamide gel electrophoresis (PAGE) gel and transferred onto PVDF transfer membrane. After blocking with 5% BSA to reduce non-specific binding, the membrane was then incubated with primary anti-PEPC antibody (Agrisera, Sweden) for 1- to 2-h and followed incubation with alkaline phosphatase conjugated secondary anti-IgG antibodies (Sigma-Aldrich, St. Louis, MO, USA) for another 1 h. The signal of target proteins was detected by using 1-Step NBT/BCIP Solution (Thermo Scientific, Wilmington, DE, USA). The PEPC activity was determined using a NADH-mediated assay system according to protocols of Shi *et al.* [106] and Gregory *et al.* [107]. The reactions were performed at 25°C and final PEPC enzyme activity was defined as oxidation of 1 mM NADH (optical density at 340 nm) per minute using SpectraMax Plus 384 (Molecular Devices, Sunnyvale, CA, USA). Approximately 1 µg purified proteins were applied in 1 mL reaction buffer, containing 50 mM HEPES-KOH (pH 8), 15% (v/v) glycerol, 2 mM PEP, 2 mM $KHCO_3$, 5 mM $MgCl_2$, 2 mM dithiothreitol, 0.15 mM NADH, and 5 units/mL malate dehydrogenase.

Supplementary References

1. Nguyen LT, Schmidt HA, von Haeseler A, Minh BQ. IQ-TREE: a fast and effective stochastic algorithm for estimating maximum-likelihood phylogenies. *Molecular Biology and Evolution* **32**, 268-274 (2015).
2. Ronquist F*, et al.* MrBayes 3.2: efficient Bayesian phylogenetic inference and model choice across a large model space. *Systematic Biology* **61**, 539-542 (2012).
3. Ogilvie HA, Bouckaert RR, Drummond AJ. StarBEAST2 brings faster species tree inference and accurate estimates of substitution rates. *Molecular Biology and Evolution* **DOI:** https://doi.org/10.1093/molbev/msx126, (2017).
4. Mirarab S, Warnow T. ASTRAL-II: coalescent-based species tree estimation with many hundreds of taxa and thousands of genes. *Bioinformatics* **31**, 44-52 (2015).
5. Conesa A, Nueda MJ, Ferrer A, Talón M. maSigPro: a method to identify significantly differential expression profiles in time-course microarray experiments. *Bioinformatics* **22**, 1096-1102 (2006).
6. Nueda MJ, Tarazona S, Conesa A. Next maSigPro: updating maSigPro bioconductor package for RNA-seq time series. *Bioinformatics* **30**, 2598-2602 (2014).
7. Margolin AA*, et al.* ARACNE: an algorithm for the reconstruction of gene regulatory networks in a mammalian cellular context. *BMC bioinformatics* **7 Suppl 1**, S7 (2006).
8. Sahm A, Bens M, Platzer M, Szafranski K. PosiGene: automated and easy-to-use pipeline for genome-wide detection of positively selected genes. *Nucleic Acids Research*, (2017).
9. Ma Y*, et al.* Regulators of PP2C phosphatase activity function as abscisic acid sensors. *Science* **324**, 1064-1068 (2009).
10. Wang Y, Noguchi K, Ono N, Inoue S-i, Terashima I, Kinoshita T. Overexpression of plasma membrane H^+-ATPase in guard cells promotes light-induced stomatal opening and enhances plant growth. *Proceedings of the National Academy of Sciences of the United States of America* **111**, 533-538 (2014).
11. Nieves-Cordones M, Caballero F, Martínez V, Rubio F. Disruption of the *Arabidopsis thaliana* inward-rectifier K^+ channel AKT1 improves plant responses to water stress. *Plant and Cell Physiology* **53**, 423-432 (2012).
12. De Angeli A, Zhang J, Meyer S, Martinoia E. AtALMT9 is a malate-activated vacuolar chloride channel required for stomatal opening in *Arabidopsis*. *Nature Communications* **4**, 1804 (2013).
13. Shang Y, Dai C, Lee MM, Kwak JM, Nam KH. BRI1-Associated Receptor Kinase 1 regulates guard cell ABA signaling mediated by Open Stomata 1 in *Arabidopsis*. *Molecular Plant* **9**, 447-460 (2016).
14. Takemiya A*, et al.* Phosphorylation of BLUS1 kinase by phototropins is a primary step in stomatal opening. *Nature Communications* **4**, (2013).
15. Geiger D*, et al.* Guard cell anion channel SLAC1 is regulated by CDPK protein kinases with distinct Ca^{2+} affinities. *Proceedings of the National Academy of Sciences of the United States of America* **107**, 8023-8028 (2010).
16. Mori IC*, et al.* CDPKs CPK6 and CPK3 function in ABA regulation of guard cell S-type anion-and Ca^{2+}-permeable channels and stomatal closure. *PLoS Biology* **4**, e327 (2006).
17. Hashimoto M, Negi J, Young J, Israelsson M, Schroeder JI, Iba K. Arabidopsis HT1 kinase controls stomatal movements in response to CO_2. *Nature Cell Biology* **8**, 391-397 (2006).
18. Szyroki A*, et al.* KAT1 is not essential for stomatal opening. *Proceedings of the National Academy of Sciences of the United States of America* **98**, 2917-2921 (2001).
19. Lebaudy A*, et al.* Plant adaptation to fluctuating environment and biomass production are strongly dependent on guard cell potassium channels. *Proceedings of the National Academy of Sciences of the United States of America* **105**, 5271-5276 (2008).

20. Mustilli A-C, Merlot S, Vavasseur A, Fenzi F, Giraudat J. *Arabidopsis* OST1 protein kinase mediates the regulation of stomatal aperture by abscisic acid and acts upstream of reactive oxygen species production. *Plant Cell* **14**, 3089-3099 (2002).
21. Merlot S*, et al.* Constitutive activation of a plasma membrane H^+ ‐ ATPase prevents abscisic acid ‐ mediated stomatal closure. *EMBO Journal* **26**, 3216-3226 (2007).
22. Kinoshita T, Doi M, Suetsugu N, Kagawa T, Wada M, Shimazaki K-i. Phot1 and phot2 mediate blue light regulation of stomatal opening. *Nature* **414**, 656-660 (2001).
23. Park S-Y*, et al.* Abscisic acid inhibits type 2C protein phosphatases via the PYR/PYL family of START proteins. *Science* **324**, 1068-1071 (2009).
24. Meyer S*, et al.* AtALMT12 represents an R ‐ type anion channel required for stomatal movement in *Arabidopsis* guard cells. *Plant Journal* **63**, 1054-1062 (2010).
25. Vahisalu T*, et al.* SLAC1 is required for plant guard cell S-type anion channel function in stomatal signalling. *Nature* **452**, 487-491 (2008).
26. Exner V*, et al.* A gain-of-function mutation of *Arabidopsis* cryptochrome1 promotes flowering. *Plant Physiology* **154**, 1633-1645 (2010).
27. El-Assal SE-D, Alonso-Blanco C, Peeters AJ, Wagemaker C, Weller JL, Koornneef M. The role of cryptochrome 2 in flowering in *Arabidopsis*. *Plant Physiology* **133**, 1504-1516 (2003).
28. Dixon LE, Knox K, Kozma-Bognar L, Southern MM, Pokhilko A, Millar AJ. Temporal repression of core circadian genes is mediated through EARLY FLOWERING 3 in *Arabidopsis*. *Current Biology* **21**, 120-125 (2011).
29. Baudry A*, et al.* F-box proteins FKF1 and LKP2 act in concert with ZEITLUPE to control *Arabidopsis* clock progression. *Plant Cell* **22**, 606-622 (2010).
30. Shen Y*, et al.* Phytochrome A mediates rapid red light–induced phosphorylation of *Arabidopsis* FAR-RED ELONGATED HYPOCOTYL1 in a low fluence response. *Plant Cell* **21**, 494-506 (2009).
31. Jarillo JA*, et al.* An *Arabidopsis* circadian clock component interacts with both CRY1 and phyB. *Nature* **410**, 487-490 (2001).
32. Alabadí D, Oyama T, Yanovsky MJ, Harmon FG, Más P, Kay SA. Reciprocal regulation between *TOC1* and *LHY/CCA1* within the *Arabidopsis* circadian clock. *Science* **293**, 880-883 (2001).
33. Pruneda-Paz JL, Breton G, Para A, Kay SA. A functional genomics approach reveals CHE as a component of the *Arabidopsis* circadian clock. *Science* **323**, 1481-1485 (2009).
34. Dalchau N*, et al.* The circadian oscillator gene *GIGANTEA* mediates a long-term response of the *Arabidopsis thaliana* circadian clock to sucrose. *Proceedings of the National Academy of Sciences of the United States of America* **108**, 5104-5109 (2011).
35. Nusinow DA*, et al.* The ELF4-ELF3-LUX complex links the circadian clock to diurnal control of hypocotyl growth. *Nature* **475**, 398-402 (2011).
36. Bauer D*, et al.* Constitutive photomorphogenesis 1 and multiple photoreceptors control degradation of phytochrome interacting factor 3, a transcription factor required for light signaling in *Arabidopsis*. *Plant Cell* **16**, 1433-1445 (2004).
37. Malapeira J, Khaitova LC, Mas P. Ordered changes in histone modifications at the core of the *Arabidopsis* circadian clock. *Proceedings of the National Academy of Sciences of the United States of America* **109**, 21540-21545 (2012).
38. Nakamichi N, Kiba T, Henriques R, Mizuno T, Chua N-H, Sakakibara H. PSEUDO-RESPONSE REGULATORS 9, 7, and 5 are transcriptional repressors in the *Arabidopsis* circadian clock. *Plant Cell* **22**, 594-605 (2010).
39. Huang W*, et al.* Mapping the core of the *Arabidopsis* circadian clock defines the network structure of the oscillator. *Science* **336**, 75-79 (2012).
40. Más P, Kim W-Y, Somers DE, Kay SA. Targeted degradation of TOC1 by ZTL modulates circadian function in *Arabidopsis thaliana*. *Nature* **426**, 567-570 (2003).

41. Rugnone ML*, et al.* LNK genes integrate light and clock signaling networks at the core of the *Arabidopsis* oscillator. *Proceedings of the National Academy of Sciences of the United States of America* **110**, 12120-12125 (2013).
42. Rawat R*, et al.* REVEILLE1, a Myb-like transcription factor, integrates the circadian clock and auxin pathways. *Proceedings of the National Academy of Sciences of the United States of America* **106**, 16883-16888 (2009).
43. Hsu PY, Devisetty UK, Harmer SL. Accurate timekeeping is controlled by a cycling activator in *Arabidopsis*. *eLife* **2**, e00473 (2013).
44. Rawat R*, et al.* REVEILLE8 and PSEUDO-REPONSE REGULATOR5 form a negative feedback loop within the *Arabidopsis* circadian clock. *PLoS Genetics* **7**, e1001350 (2011).
45. Perales M, Portolés S, Más P. The proteasome ‐ dependent degradation of CKB4 is regulated by the *Arabidopsis* biological clock. *Plant Journal* **46**, 849-860 (2006).
46. Kim J, Kim Y, Yeom M, Kim J-H, Nam HG. FIONA1 is essential for regulating period length in the *Arabidopsis* circadian clock. *Plant Cell* **20**, 307-319 (2008).
47. Toledo-Ortiz G*, et al.* The HY5-PIF regulatory module coordinates light and temperature control of photosynthetic gene transcription. *PLoS Genetics* **10**, e1004416 (2014).
48. Jones MA, Covington MF, DiTacchio L, Vollmers C, Panda S, Harmer SL. Jumonji domain protein JMJD5 functions in both the plant and human circadian systems. *Proceedings of the National Academy of Sciences of the United States of America* **107**, 21623-21628 (2010).
49. Wang Y, Wu J-F, Nakamichi N, Sakakibara H, Nam H-G, Wu S-H. LIGHT-REGULATED WD1 and PSEUDO-RESPONSE REGULATOR9 form a positive feedback regulatory loop in the *Arabidopsis* circadian clock. *Plant Cell* **23**, 486-498 (2011).
50. Hong S, Song H-R, Lutz K, Kerstetter RA, Michael TP, McClung CR. Type II protein arginine methyltransferase 5 (PRMT5) is required for circadian period determination in *Arabidopsis thaliana*. *Proceedings of the National Academy of Sciences of the United States of America* **107**, 21211-21216 (2010).
51. Wang X*, et al.* SKIP is a component of the spliceosome linking alternative splicing and the circadian clock in *Arabidopsis*. *Plant Cell* **24**, 3278-3295 (2012).
52. Jones MA, Williams BA, McNicol J, Simpson CG, Brown JW, Harmer SL. Mutation of *Arabidopsis SPLICEOSOMAL TIMEKEEPER LOCUS1* causes circadian clock defects. *Plant Cell* **24**, 4066-4082 (2012).
53. Panda S, Poirier GG, Kay SA. *tej* defines a role for poly (ADP-ribosyl) ation in establishing period length of the *Arabidopsis* circadian oscillator. *Developmental Cell* **3**, 51-61 (2002).
54. Osmond C, Holtum J. Crassulacean acid metabolism. In: *The Biochemistry of Plants, Volume 8: Photosynthesis* (ed Hatch M, Boardman N). Academic Press (1981).
55. Borland AM*, et al.* Engineering crassulacean acid metabolism to improve water-use efficiency. *Trends in Plant Science* **19**, 327-338 (2014).
56. Qian W, Zhang J. Genomic evidence for adaptation by gene duplication. *Genome Research* **24**, 1356-1362 (2014).
57. Ming R*, et al.* The pineapple genome and the evolution of CAM photosynthesis. *Nature Genetics* **47**, 1435-1442 (2015).
58. Abraham PE*, et al.* Transcript, protein and metabolite temporal dynamics in the CAM plant *Agave*. *Nature Plants* **2**, Article number: 16178 (2016).
59. Hartwell J, Nimmo G, Wilkins M, Jenkins G, Nimmo H. Phospho*enol*pyruvate carboxylase kinase is a novel protein kinase is a novel protein kinase regulated at the level of gene expression. *Plant Journal* **20**, 333-342 (1999).
60. Hafke JB, Hafke Y, Smith JAC, Lüttge U, Thiel G. Vacuolar malate uptake is mediated by an anion ‐ selective inward rectifier. *Plant Journal* **35**, 116-128 (2003).

61. Meyer S, *et al.* Malate transport by the vacuolar AtALMT6 channel in guard cells is subject to multiple regulation. *Plant Journal* **67**, 247-257 (2011).
62. Smith JAC, *et al.* Transport across the vacuolar membrane in CAM plants. In: *Crassulacean Acid Metabolism* (ed Winter K, Smith J). Springer-Verlag (1996).
63. Emmerlich V, *et al.* The plant homolog to the human sodium/dicarboxylic cotransporter is the vacuolar malate carrier. *Proceedings of the National Academy of Sciences of the United States of America* **100**, 11122-11126 (2003).
64. Dittrich P, Campbell WH, Black CC. Phosphoenolpyruvate carboxykinase in plants exhibiting crassulacean acid metabolism. *Plant Physiology* **52**, 357-361 (1973).
65. Dever LV, Boxall SF, Kneřová J, Hartwell J. Transgenic perturbation of the decarboxylation phase of crassulacean acid metabolism alters physiology and metabolism but has only a small effect on growth. *Plant Physiology* **167**, 44-59 (2015).
66. Hartwell J, Dever LV, Boxall SF. Emerging model systems for functional genomics analysis of crassulacean acid metabolism. *Current Opinion in Plant Biology* **31**, 100-108 (2016).
67. Kliebenstein D. Secondary metabolites and plant/environment interactions: a view through *Arabidopsis thaliana* tinged glasses. *Plant, Cell & Environment* **27**, 675-684 (2004).
68. Maeda H, Dudareva N. The shikimate pathway and aromatic amino acid biosynthesis in plants. *Annual Review of Plant Biology* **63**, 73-105 (2012).
69. Delker C, Zolman BK, Miersch O, Wasternack C. Jasmonate biosynthesis in *Arabidopsis thaliana* requires peroxisomal β-oxidation enzymes–Additional proof by properties of *pex6* and *aim1*. *Phytochemistry* **68**, 1642-1650 (2007).
70. Munemasa S, Hossain MA, Nakamura Y, Mori IC, Murata Y. The *Arabidopsis* calcium-dependent protein kinase, CPK6, functions as a positive regulator of methyl jasmonate signaling in guard cells. *Plant Physiology* **155**, 553-561 (2011).
71. Proost S, *et al.* PLAZA 3.0: an access point for plant comparative genomics. *Nucleic Acids Research* **43**, D974-981 (2015).
72. Cai J, *et al.* The genome sequence of the orchid *Phalaenopsis equestris*. *Nature Genetics* **47**, 65-72 (2015).
73. Carrier G, *et al.* An efficient and rapid protocol for plant nuclear DNA preparation suitable for next generation sequencing methods. *American Journal of Botany* **98**, e13-15 (2011).
74. Smit AFA, Hubley R. RepeatModeler Open-1.0. http://wwwrepeatmaskerorg, (2008-2015).
75. Haas BJ, *et al.* Improving the *Arabidopsis* genome annotation using maximal transcript alignment assemblies. *Nucleic Acids Research* **31**, 5654-5666 (2003).
76. Smit AFA, Hubley R, Green P. RepeatMasker Open-3.0. http://wwwrepeatmaskerorg, (1996-2012).
77. Salamov AA, Solovyev VV. Ab initio gene finding in *Drosophila* genomic DNA. *Genome Research* **10**, 516-522 (2000).
78. Yeh RF, Lim LP, Burge CB. Computational inference of homologous gene structures in the human genome. *Genome Research* **11**, 803-816 (2001).
79. Li L, Stoeckert CJ, Jr., Roos DS. OrthoMCL: identification of ortholog groups for eukaryotic genomes. *Genome Research* **13**, 2178-2189 (2003).
80. Katoh K, Standley DM. MAFFT multiple sequence alignment software version 7: improvements in performance and usability. *Molecular Biology and Evolution* **30**, 772-780 (2013).
81. Suyama M, Torrents D, Bork P. PAL2NAL: robust conversion of protein sequence alignments into the corresponding codon alignments. *Nucleic Acids Research* **34**, W609-612 (2006).
82. Kuck P, Meusemann K. FASconCAT: Convenient handling of data matrices. *Molecular Phylogenetics and Evolution* **56**, 1115-1118 (2010).

83. Minh BQ, Nguyen MA, von Haeseler A. Ultrafast approximation for phylogenetic bootstrap. *Molecular Biology and Evolution* **30**, 1188-1195 (2013).
84. Kück P, Longo GC. FASconCAT-G: extensive functions for multiple sequence alignment preparations concerning phylogenetic studies. *Frontiers in Zoology* **11**, (2014).
85. Darriba D, Taboada GL, Doallo R, Posada D. ProtTest 3: fast selection of best-fit models of protein evolution. *Bioinformatics* **27**, 1164-1165 (2011).
86. Stamatakis A. RAxML-VI-HPC: maximum likelihood-based phylogenetic analyses with thousands of taxa and mixed models. *Bioinformatics* **22**, 2688-2690 (2006).
87. Castoe TA*, et al.* Evidence for an ancient adaptive episode of convergent molecular evolution. *Proceedings of the National Academy of Sciences of the United States of America* **106**, 8986-8991 (2009).
88. Qian C, Bryans N, Kruykov I, de Koning APJ. Visualization and analysis of statistical signatures of convergent molecular evolution. *University of Calgary* http://labjasondkio, (2015).
89. Qian C, de Koning APJ. Rapid discovery of convergent molecular evolution across entire phylogenies. *University of Calgary* http://labjasondkio, (2015).
90. Bindea G*, et al.* ClueGO: a Cytoscape plug-in to decipher functionally grouped gene ontology and pathway annotation networks. *Bioinformatics* **25**, 1091-1093 (2009).
91. Holm S. A simple sequentially rejective multiple test procedure. *Scandinavian Journal of Statistics* **6**, 65-70 (1979).
92. Moriya Y, Itoh M, Okuda S, Yoshizawa AC, Kanehisa M. KAAS: an automatic genome annotation and pathway reconstruction server. *Nucleic Acids Research* **35**, W182-W185 (2007).
93. Langfelder P, Horvath S. WGCNA: an R package for weighted correlation network analysis. *BMC Bioinformatics* **9**, 559 (2008).
94. Langfelder P, Horvath S. Fast R functions for robust correlations and hierarchical clustering. *Journal of Statistical Software* **46**, i11 (2012).
95. Kumar L, E. Futschik M. Mfuzz: A software package for soft clustering of microarray data. *Bioinformation* **2**, 5-7 (2007).
96. Dai X, Sinharoy S, Udvardi M, Zhao PX. PlantTFcat: an online plant transcription factor and transcriptional regulator categorization and analysis tool. *BMC bioinformatics* **14**, 321 (2013).
97. Shannon P*, et al.* Cytoscape: a software environment for integrated models of biomolecular interaction networks. *Genome Research* **13**, 2498-2504 (2003).
98. Banerjee S, Pedersen T. The design, implementation, and use of the Ngram statistics package. In: *Computational Linguistics and Intelligent Text Processing. CICLing 2003. Lecture Notes in Computer Science, vol 2588* (ed Gelbukh A). Springer (2003).
99. Benjamini Y, Hochberg Y. Controlling the false discovery rate: a practical and powerful approach to multiple testing. *Journal of the Royal Statistical Society Series B* **57**, 289-300 (1995).
100. Kielbasa SM, Wan R, Sato K, Horton P, Frith MC. Adaptive seeds tame genomic sequence comparison. *Genome Research* **21**, 487-493 (2011).
101. Larkin MA*, et al.* Clustal W and Clustal X version 2.0. *Bioinformatics* **23**, 2947-2948 (2007).
102. Yang Z. PAML 4: phylogenetic analysis by maximum likelihood. *Molecular Biology and Evolution* **24**, 1586-1591 (2007).
103. Wang Y*, et al.* MCScanX: a toolkit for detection and evolutionary analysis of gene synteny and collinearity. *Nucleic Acids Research* **40**, e49 (2012).
104. Tuskan GA*, et al.* The genome of black cottonwood, *Populus trichocarpa* (Torr. & Gray). *Science* **313**, 1596-1604 (2006).
105. Tschaplinski TJ*, et al.* Down-regulation of the caffeic acid *O*-methyltransferase gene in switchgrass reveals a novel monolignol analog. *Biotechnology for Biofuels* **5**, 71 (2012).

106. Shi J*, et al.* Phosphoenolpyruvate carboxylase in *Arabidopsis* leaves plays a crucial role in carbon and nitrogen metabolism. *Plant Physiology* **167**, 671-681 (2015).
107. Gregory AL*, et al. In vivo* regulatory phosphorylation of the phosphoenolpyruvate carboxylase AtPPC1 in phosphate-starved *Arabidopsis thaliana*. *Biochemical Journal* **420**, 57-65 (2009).
