# Supplementary material for: The Kalanchoë genome provides insights into convergent evolution and building blocks of crassulacean acid metabolism
Source: Nat Commun. 2017 Dec 1;8:1899. doi: 10.1038/s41467-017-01491-7 (PMC5711932; doi:10.1038/s41467-017-01491-7)
Supplement: Supplementary file 2 — Description of Additional Supplementary Files [file 41467_2017_1491_MOESM2_ESM.pdf]

Description of Additional Supplementary Files

File Name: Supplementary Dataset 1
Description: Biological processes enriched in co-expression modules. See separate Excel file.

File Name: Supplementary Dataset 2
Description: The hub genes with the top 1% most connected nodes as well as putative CAM genes with at least 10 directed edges from each gene cluster shown in Supplementary Table 7 and Supplementary Fig. 11. See separate Excel file

File Name: Supplementary Dataset 3
Description: List of Kalanchoë genes showing convergent changes in diel expression pattern. See separate Excel file

File Name: Supplementary Dataset 4
Description: Gene count of CAZyme module genes in thirteen plant species. See separate Excel file.

File Name: Supplementary Data 5
Description: List of predicted CAZyme genes in Kalanchoë fedtschenkoi. See separate Excel file.
